# Macrophages modulate fibrosis during newt lens regeneration

**DOI:** 10.1186/s13287-024-03740-1

**Published:** 2024-05-14

**Authors:** Georgios Tsissios, Anthony Sallese, J. Raul Perez-Estrada, Jared A. Tangeman, Weihao Chen, Byran Smucker, Sophia C. Ratvasky, Erika Grajales-Esquivel, Arielle Martinez, Kimberly J. Visser, Alberto Joven Araus, Hui Wang, András Simon, Maximina H. Yun, Katia Del Rio-Tsonis

**Affiliations:** 1 Department of Biology, Miami University, Oxford, OH USA; 2 Center for Visual Sciences at, Miami University, Oxford, OH USA; 3 Cellular Molecular and Structural Biology Program, Miami University, Oxford, OH USA; 4 Department of Chemical, Paper and Biomedical Engineering, Miami University, Oxford, OH USA; 5 Department of Statistics, Miami University, Oxford, OH USA; 6 Department of Cell and Molecular Biology, Karolinska Institute, Stockholm, Sweden; 7 CRTD/ Center for Regenerative Therapies Dresden, Technische Universität Dresden, Dresden, Germany; 8 Max Planck Institute of Molecular Cell Biology and Genetics, Dresden, Germany; 9 Cluster of Excellence Physics of Life, Technische Universität Dresden, Dresden, Germany

**Keywords:** Lens regeneration, Macrophage, Tissue repair, Inflammation

## Abstract

**Background:**

Previous studies have suggested that macrophages are present during lens regeneration in newts, but their role in the process is yet to be elucidated.

**Methods:**

Here we generated a transgenic reporter line using the newt, *Pleurodeles waltl*, that traces macrophages during lens regeneration. Furthermore, we assessed early changes in gene expression during lens regeneration using two newt species, *Notophthalmus viridescens* and *Pleurodeles waltl*. Finally, we used clodronate liposomes to deplete macrophages during lens regeneration in both species and tested the effect of a subsequent secondary injury after macrophage recovery.

**Results:**

Macrophage depletion abrogated lens regeneration, induced the formation of scar-like tissue, led to inflammation, decreased iris pigment epithelial cell (iPEC) proliferation, and increased rates of apoptosis in the eye. Some of these phenotypes persisted throughout the last observation period of 100 days and could be attenuated by exogenous FGF2 administration. A distinct transcript profile encoding acute inflammatory effectors was established for the dorsal iris. Reinjury of the newt eye alleviated the effects of macrophage depletion, including the resolution of scar-like tissue, and re-initiated the regeneration process.

**Conclusions:**

Together, our findings highlight the importance of macrophages for facilitating a pro-regenerative environment in the newt eye by regulating fibrotic responses, modulating the overall inflammatory landscape, and maintaining the proper balance of early proliferation and late apoptosis of the iPECs.

**Supplementary Information:**

The online version contains supplementary material available at 10.1186/s13287-024-03740-1.

## Background

A popular hypothesis in regenerative biology suggests that adult mammals have developed a more robust adaptive immune response at the expense of regenerative capabilities [1]. However, NOD/SCID mice that exhibit T cell deficiency but retain macrophage numbers fail to regenerate their hearts at neonatal stages and demonstrate signs of severe fibrosis, suggesting that modulating adaptive immunity alone is not sufficient for successful regeneration [2]. Furthermore, the theory of inverse relationship between regeneration and immune proficiency fails to explain why several mammals are capable of epimorphic-like regeneration as adults [3–5]. An alternative theory suggests that the interaction between immune cells and the local microenvironment influences the capacity for regeneration, rather than the absence or presence of special immune and regenerative cells [6].

Macrophage involvement during tissue regeneration has been a subject of intense discussion in recent years [7–11]. There is now increasing evidence that macrophages play a necessary role during limb, fin, tail, spinal cord, and heart regeneration in a variety of species [12–27]. The regenerative processes in these tissues and organs are complex, require the integration of multiple cell populations, and are not easily accessible for experimentation and visualization [28–31]. This added level of complexity makes it difficult to extrapolate immune mechanisms in a regenerative context. On the other hand, the case of lens regeneration in newts involves the transdifferentiation of a single cell type, the iris pigment epithelial cells (iPECs), into lens cells [32–37]. The elegant simplicity of this process serves as a unique platform to uncover the mechanisms by which macrophages promote or interfere with scar-free healing and regeneration.

In the past, electron microscopy studies recognized that macrophages, which were first called “special amoeboid cells” due to their morphology, migrated inside the iris epithelium at 3 days post-lentectomy (dpl) and phagocytosed melanosomes that were discharged from iPECs during the dedifferentiation process [38–42]. In our study, we present and characterize a new *Pleurodeles waltl* (*P. waltl*) transgenic line that enables the study of macrophages during the regeneration process. Using bulk RNA sequencing, we characterize the immune landscape of the iris after injury and investigate the role of macrophages in directing the early and late stages of lens regeneration. We show how macrophage depletion modulates the wound healing response and affects the regenerative outcome. In addition, we demonstrate that macrophages returning into the deleterious wound lesion were unable to resolve the inflammatory and fibrotic environment. Nevertheless, a secondary injury alone or the addition of fibroblast growth factor 2 (FGF2) is sufficient to initiate scar resolution and restart the regeneration process.

## Methods

### Animal husbandry and ethical statement

Iberian ribbed newts, *P. waltl*, and red spotted eastern newts, Notophthalmus viridescens (*N. viridescens*) were used in this study. Adult *P. waltl* wildtype newts born and raised in captivity for generations were used for transgenesis in the aquatic facilities of Karolinska Institutet, Stockholm. Handling, breeding, transgenesis and the other experimental procedures performed in Stockholm were done according to both Swedish and European regulations. All animals were raised according to previously established husbandry guidelines [43]. A group of Iberian ribbed newts were transferred to the newt colony at Miami University, where they were further bred and grown to pursue this study. The red spotted eastern newts were wild caught. Handling and surgical procedures were performed following guidelines by the Institutional Animal Care and Use Committee at Miami University.

It is important to note that the lens growth rates and speed of regeneration can vary between different newt species and between different ages of the same species [35, 44, 45]. For this reason, we refer to lens regeneration stages (as defined in [44]), in addition to days post- lentectomy, throughout the manuscript.

### Generation of Tol2-mpeg1:eGFP-polyA plasmid

A vector containing a 1.86 kb fragment of the zebrafish *mpeg1* promoter driving eGFP, as previously described [46], was a kind gift from Enrique Amaya. The mpeg1 fragment was amplified by PCR (*mpeg1 F* 5′-TTGGAGCACATCTGAC-3′; *mpeg1 R* 5′-TTTTGCTGTCTCCTGCAC-3′) and subcloned into pBSII-SK-mTol2 upstream of the coding region of eGFP. SV40 polyA was used as a termination signal. The resulting plasmid was purified using cesium chloride preparation to avoid potential contaminants that may negatively impact the transgenesis efficiency or newt survival. The plasmid was subsequently purified using Qiagen Maxiprep according to manufacturer’s instructions and then resuspended in ultrapure distilled water.

### ***Pleurodeles waltl*** transgenesis: tgTol2(Dre.***mpeg1:eGFP***)^MHY/SIMON^

**Fig. 1:**
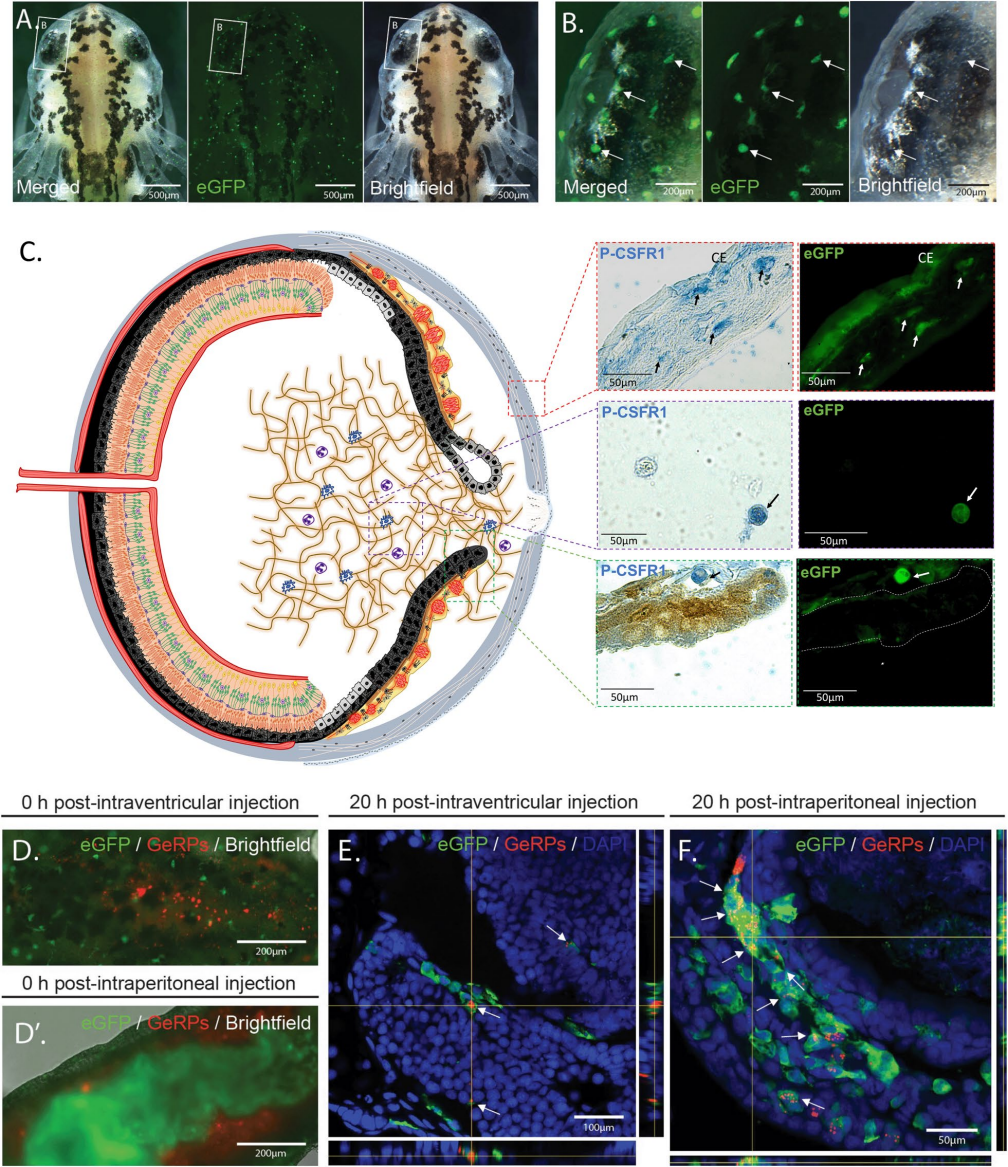
*mpeg1:GFP* transgenic newts enable the in vivo labeling of macrophages. **A** Representative images of an mpeg1:GFP + initial larva (Developmental Stage 34) showing the widespread distribution of eGFP + cells. Scale bar: 500 µm; n = 3. **B** Image of the initial larval eye showing the region indicated in (**A**) at higher magnification. Arrows point to mpeg1:GFP + cells showing spherical, amoeboid and dendritic morphology. Scale bar: 200 µm. **C** (Right) Double staining (paraffin embedded tissue) for eGFP and the macrophage marker P-CSFR1 in eye tissues such as cornea, iris stroma and vitreous chamber. Arrows highlight cells positive for both markers. Scale bars: 50 µm; n = 4; CE= cornea epithelium. **C** (Left) Schematic drawing of the newt eye at 4 dpl (Stage 0–I). **D** Image of the dorsal view of the brain area of an mpeg1:GFP larva showing successful intraventricular injection of glucan-encapsulated siRNA particles (GeRPs). Scale bar: 200 µm. **D'** Image of the ventral view of the abdominal region of an mpeg1:GFP larva showing successful intraperitoneal injection of GeRPs. Scale bar: 200 µm. **E** The vast majority of GeRPs were found inside the eGFP + cells (arrows) in the brain 20 h post-intraventricular injection (O.C.T./Cryo embedded tissue). Scale bar: 100 µm; n = 3. **F** Similarly, the eGFP + cells located in the intraperitoneal cavity were able to engulf most of the GeRPs (arrows) 20 h after intraperitoneal injection (O.C.T./Cryo embedded tissue). Scale bar: 50 µm; n = 2

Approximately 5 nl of a mix composed of 1 µl of Tol2-mpeg1:eGFP-polyA plasmid (100 ng/ml) and 3 µl of Tol2 transposase (300 ng/ml) was injected into single-cell eggs to generate transgenic founders (F_0_), adapting previously described procedures to *P. waltl* [47]. The founder generation of mpeg1:GFP newts were bred with albino newts (Tyr^−/−^) to establish the transgenic line [48]. Every clutch from the first generation (F_1_) onwards was screened with a fluorescence microscope to select for the positive offspring (Fig. 1A). Only animals from generation F_1_ onwards were used in this study.

### Phagocytosis assay: glucan-encapsulated siRNA particles (GeRPs) injections

GeRPs were a kind gift of Myriam Aouadi [49–51]. 1 mg of GeRPs containing Rhodamine were resuspended in 1 ml of PBS and sonicated just before injection. The sonication protocol was: 40%, 10 s; 35%, 10 s; 30%, 10 s; 25%, 10 s; 20%, 10 s; 30%, 30 s. GeRPs were injected using pulled borosilicate glass capillaries (Harvard Apparatus, GC100F-10) in transgenic mpeg1:GFP *Pleurodeles* [F_1_: tgTol2(Dre.mpeg1:eGFP)MHY/Simon] (n = 5), either intraperitoneally (n = 2) or intraventricularly (n = 3), according to a previous established protocol [52]. Live imaging of larvae and time lapse movies were created with Zeiss Axiovert 200 M inverted microscope. For histological observations, larvae were fixed at 20 h post-injection and processed for immunofluorescence according to Joven et al., 2018 [53].

### Lentectomy, iridectomy and EdU injections

Animals were anesthetized by whole body submersion in 0.1% ethyl 3-aminobenzoate-methane Sulfonic acid solution (MilliporeSigma) diluted in amphibian phosphate-buffered saline (APBS:1 × PBS plus 25% dH2O). Once animals were anesthetized, a scalpel was used to make a slit in the cornea and the entire lens was carefully removed with fine tweezers [54]. Clodronate or PBS liposomes (Encapsula Nano Science, # CLD-8901) were injected intraocularly into the vitreous cavity of 40 newt eyes using a pre-pulled glass needle (20 μm tip diameter) attached to a microinjector (MicroJect 1000A, BTX, Harvard Apparatus) set at 10.5psi. For iridectomy experiments, the apical region of the dorsal iris (and regenerating lens from PBS-liposomes treated eyes) was surgically removed at 60 days post-lentectomy (dpl) by re-opening the cornea with a scalpel and removing a piece of the iris using scissors and tweezers (n = 8 for clodronate treated eyes, n = 8 for PBS treated eyes). After all surgical procedures, the animals were allowed to recover from anesthesia and return to appropriate housing containers where they were monitored carefully for the duration of the experiments. For cell proliferation studies, EdU (Invitrogen, #C10338) was injected intraperitoneally 24 h prior to collection at 10 µg/g of body weight. All animals used for these experiments were post-metamorphotic juveniles at 6–8 months old.

### FGF2 experiments

Heparin-coated polyacrylamide beads (Sigma, #H-5263) were washed in APBS and incubated either with 0.25 μg/μl bFGF2 (n = 12) (R&D Systems, #133-FB) or APBS (vehicle control) (n = 12) overnight at 4 °C. Heparin beads were carefully inserted with tweezers into the vitreous chamber of the eye, between the dorsal and ventral iris, following lens removal. All animals used for these experiments were post-metamorphotic juveniles at 6–8 months old.

### *Notophthalmus viridescens* RNAseq, transcriptome assembly, and differential expression analysis

Dorsal iris tissues were collected from adult *N. viridescens* from intact animals (no lentectomy), as well as animals 6 h post-lentecomy (hpl), 1 dpl, and 4 dpl. Three biological replicates were used per time point, with each biological replicate containing bilateral dorsal irises from 3 or 4 animals pooled together. The iris tissues were placed in 500μL of cold TRI Reagent (Zymo, R2050-1-50), vortexed, and stored at − 80 °C until RNA extraction. RNA isolation was performed via extraction with 0.2 volumes chloroform followed by processing of the aqueous phase with the Direct-zol RNA Microprep Kit (Zymo, R2060) according to manufacturer’s instructions, including an in-column DNase I treatment. RNA integrity was assessed with the Agilent RNA 6000 Pico Kit (Agilent, 5067-1513) and quantification performed with the Qubit RNA HS Assay Kit (ThermoFisher, Q32852). Reverse transcription and library preparation were carried out with the Zymo-Seq RiboFree total RNA library Kit (Zymo, R3000) using 108 ng input RNA per reaction. RiboFree depletion was applied for 4 h to deplete overrepresented transcripts, and final libraries were amplified with dual indexes (Zymo, D3096) for 13 PCR cycles. Sequencing was performed at the Novogene sequencing core on the Illumina NovaSeq 6000 to approx. 100 million paired-end reads per sample.

The first 10 base pairs of sequence read2 were hard trimmed to remove low-complexity bridge sequences introduced during library preparation. The reads were then quality and adapter trimmed with Trim Galore using parameters -*q 5 --length 36 --stringency 1 -e 0.1* [55, 56]. rRNA reads were depleted by aligning trimmed reads against the Silva rRNA database using Bowtie2 [57, 58]. Cleaned reads were prepared for transcriptome assembly using the Trinity assembler pre-processing script insilico_read_normalization.pl with parameters --*seqType fq *--*JM 1450G *--*max_cov 30 *--*pairs_together *--*SS_lib_type RF -CPU 24 *--*PARALLEL_STATS *--*KMER_SIZE 25 *--*max_CV 10000 *--*min_cov 2 *[59]. Trinity assembly was performed on each condition individually with Trinity using *parameters *--*seqType fq *--*max_memory 1450G *--*SS_lib_type RF *--*CPU 24 *--*min_contig_length 200 *--*monitoring *--*min_kmer_cov 2 *--*no_normalize_reads*. Individual assemblies were merged into a final transcriptome with the DRAP pipeline runMeta tool and parameters --*strand RF *--*mapper bwa *--*length 200 *--*type contig *--*coverage 0,1,10 *--*write* [60]. Assembled transcripts were annotated against the NCBI non-redundant database using an implementation of blastx and the functional annotation module in the OmicsBox software environment [61]. Final transcript abundance was estimated with the Salmon alignment tool using the parameters *-l ISR *--*numGibbsSamples 20 *--*seqBias *--*gcBias *--*reduceGCMemory -d* [62]. Differential transcript abundance testing was performed in the R environment using the SWISH Fishpond workflow [63]. Expression values displayed in manuscript are the log-transformed Transcript Per Million (TPM) values, averaged across inferential replicates. In the case of transcripts with multiple detected isoforms, the transcripts displayed in the heatmap represent the transcript assigned the lowest E-value determined by blastx. KEGG pathway enrichment analysis was performed using the Combined Pathway Module inside the OmicsBox environment, with significance values calculated using maSigPro time-course analysis [64, 65]. For combined pathway analysis, parameters were set as *Keep most specific pathways* = TRUE and *Blast expectation value* = *0.*001, and KEGG orthologs were linked through the EggNog mapper [66]. A two tailed Fisher’s test was applied to pathways and FDR cut off was applied at 0.05.

### *Pleurodeles waltl* RNAseq and differential expression analysis

Dorsal iris tissue was dissected from 13-month-old newts using intact or lentectomized animals (1 and 4 dpl). Tissue from 4 animals were pooled for each biological replicate, collected in triplicate. Samples were collected into cold TRI Reagent (Zymo, R2050-1-50), vortexed, and stored at − 80 °C until RNA extraction, as described above. RNAseq libraries were prepared using 72–130 ng of isolated RNA, using NEBNext® Ultra™ II Directional RNA Library Prep with Sample Purification Beads (NEB, E7765S) and NEBNext® Poly(A) mRNA Magnetic Isolation Module (E7490S). Indexing was performed using NEBNext® Multiplex Oligos for Illumina® (NEB, E7500/7710/7730). Samples were pooled and sequenced on a lane of NovaSeq 6000 at the Novogene Sequencing Core using paired-end, 150 base pair reads to a minimum depth of 29 million read pairs per sample.

Reads were quality trimmed using Trim Galore with parameters --*stringency 3 *--*paired *--*length 36* [55, 56]. Cleaned reads were aligned to the *P. waltl* genome using the STAR aligner with two-pass alignment to insert splice junctions [67, 68]. Aligned reads were assembled into transcripts using Stringtie and spliced transcripts were extracted using gffread [69, 70]. Assembled transcripts were indexed using the Salmon quantification tool, using the parameter *-k 31* and providing genomic sequence as decoy [62]. Transcript expression was quantified using salmon with parameters *-l ISR --validateMappings -p 12 --numGibbsSamples 20 --seqBias --gcBias -d*. Samples with alignment rates > 80% were included in downstream analysis, resulting in the exclusion of one sample (intact replicate 3). Expression values displayed in manuscript are the log-transformed Transcript Per Million (TPM) values, averaged across inferential replicates. Assembled transcripts were annotated against the NCBI non-redundant database using an implementation of blastx and the functional annotation module in the OmicsBox software environment [61].

### RT-qPCR design and analysis

Whole eyes were enucleated at the indicated time points, placed in 500μL of TRIzol, and stored at − 20 °C. The tissues were then homogenized mechanically using pellet pestles and centrifuged to remove debris. Total RNA was isolated using Direct-zol RNA Microprep (Zymo Research, #R2061) following manufacturer’s instructions. RNA yield and quality were analyzed using Nanodrop ND-2000 Spectrophotometer (Thermo Scientific) and Agilent 2100 Bioanalyzer (Agilent Technologies), respectively. cDNA was synthesized using 200 ng of total RNA as a template with QuantiTect Reverse Transcription kit (Qiagene, #205,313) according to manufacturer’s instructions. The synthesized cDNA was diluted at 1:10 ratio with pure water. 2 ul of the cDNA dilution were used for quantitative PCR (qPCR) reactions. The final qPCR reactions contained: 2 ul of diluted cDNA, 10 ul of TB Green® Advantage® qPCR Premix (Takara, #639,676) and 50 nM of each primer, adjusted to 20 ul with water. qPCR reactions were set up in duplicate in the Rotor-Gene Q thermocycler 5 plex (Qiagene, Germantown, MD, USA) using annealing temperature set at 60 °C. Primers reported here were designed using primer blast (https://www.ncbi.nlm.nih.gov/tools/primer-blast/) and obtained from IDT Technologies. The gene coding sequences were obtained from iNewt [71]. Primer and target sequences are shown in Additional file 2: Table S1. TAII70, was used as a housekeeping gene using primers published previously [72]. The comparative Ct method was used to determine relative gene expression levels compared to the housekeeping gene. The primers and qPCR reactions were validated following qPCR MIQE guidelines [73]. Four to eight biological samples were used per condition.

To improve data conformance to statistical modeling assumptions, a natural log transformation was applied to relative mRNA values. For time course analysis of intact eyes through 30 dpl eyes (Fig. 4A), a two-way block ANOVA was conducted, with treatment and time as the factors along with their interaction. The blocking factor was included to account for batch effects, as animals were collected in two groups. The analysis was done using the aov function in the R stats package [74]. Treatment differences were estimated for each time point using the emmeans function in R, and since five comparisons were made, the *p*-values were corrected by controlling the false discovery rate [75–77]. For experiments in which transcript expression was assayed at two time points (Fig. 5D), a two-way ANOVA with interaction (factors Treatment and Time) was performed using the lm function in R, part of the stats package. For each gene, analysis of treatment means and FDR multiple comparisons correction was performed for the treatments vs. control at both 4 and 10 dpl. For the ANOVAs, residuals were examined to check for severe assumption violations, including constant error variance and normality. For experiments with a single time point (Fig. 6E), a Welch's two-sample t-test was applied using the R function t.test, part of the stats package. All animals used for these experiments were post-metamorphotic juveniles at 6–8 months old. For more information on statistical analysis, see Additional file 1: Appendix 1.

### Optical coherence tomography

The anterior chamber of each eye was non-invasively monitored via spectral domain optical coherence tomography (SD-OCT), as previously described [78]. The animals were anesthetized prior to imaging, and 100 µL of water was applied to the cornea surface with a pipette to reduce reflection artifacts caused by dehydration during live imaging (n = 10 clodronate treated eyes, n = 10 PBS liposome treated eyes). A broadband light source centered at 850nm was employed to generate OCT images with an axial resolution of 2 µm and lateral resolution of 8 µm. The scanning area of the eye was 1.2 mm by 1.2 mm, where 500 B-Scans were collected across this range. Each B-Scan consisted of 2000 A-Scan, where each A-Scan had 2048 pixels. The final C-Scans were rescaled and reconstructed as 500 × 500x 500 voxels. All animals used for these experiments were post-metamorphotic juveniles at 6–8 months old.

### Tissue embedding and sectioning

Collected tissues were washed 2 to 3 times in PBS and fixed overnight in 4% PFA at 4 °C. For cryoprotection, tissues were transferred to new tubes with 30% sucrose (in 0.1 M PBS) overnight at 4 °C. Tissues were placed in embedding molds, correctly positioned, and tissue tek embedding media (yellow Shandon Cryochrome, Thermo Fisher Scientific, Waltham, USA) was gently added. The molds were then placed at − 80 °C for 15 min and transferred to − 20 °C where they were stored until sectioning. For sectioning, a cryostat was used with an object temperature of − 21 °C and knife temperature of − 19 °C. For experiments involving whole animals, 12 µm sections were made. Sections were collected on Superfrost Plus slides and stored at − 20 °C. Tissue processing for the phagocytic experiments depicted in Fig. 1E, F were done according to Joven et al., 2018 [53]: in brief, gelatin-embedding and colder temperatures (− 30 °C) were used for sectioning.

For paraffin embedding, whole eyes were washed 3 times in PBS and fixed overnight in 10% formalin at 4 °C. For intact lenses or later time points (100 dpl) when the lens is large, eyes were fixed in methanol: acetic acid (3:1 ratio) overnight at 4 °C to preserve lens morphology. Paraffin embedded tissues were sectioned at 10 μm thickness using a microtome.

### Immunofluorescent staining

Slides with cryo-sectioned eyes were air-dried for 30 min at room temperature (RT) and thereafter washed 3 times with PBST (0.2% Triton in 1 L PBS) for 10 min. To make the tissue more accessible to antibodies, a retrieval procedure was used. Plastic containers were filled with a citrate buffer (120 µL antigen unmasking solution (Vector, Peterborough, UK) in 11.88 mL PBS and preheated in a water bath to 86 °C. Slides were incubated for 10 min. Thereafter, slides were placed in a glass container with PBS at RT to cool down. Blocking buffer (10% goat or donkey serum in 0.2% PBST) was added to the slides for 1 h at RT. Subsequently, primary antibodies were added to the slides and incubated overnight at 4 °C. List of primary antibodies and concentrations used can be found below. Slides were washed 3 times with PBST for 10 min. Appropriate secondary antibodies (AlexaFluor 488 or 594 conjugates, ThermoFisher) were added to the slides and incubated for 2–4 h at RT protected from light. Slides were washed 3 times with PBST for 10 min. Hoechst (1:10,000 in PBS) was added for 15 min at RT and protected from light. Slides were washed 3 times with PBST for 10 min and mounted with Fluorescent Mounting Medium (Sigma, #F-4680). Immunofluorescent staining procedures for the phagocytic experiments were done in larvae according to Joven et al., 2018 [53]. The same protocol was followed for paraffin embedded tissues, with additional deparaffinization steps. The deparaffination steps involved two xylene washes for 5 min, and gradually rehydration of the tissue by 1-min washes with 100%,95%,80%70%,50%,30% ethanol followed by three PBS washes. All animals used for these experiments were post-metamorphotic juveniles at 6–8 months of age, with the exception of the MPEG transgenic animals, which were 3 years old.

#### Antibodies

⍺-A-Crystallin (Gift by G. Eguchi, no dilution), Phospho-Histone H3 (Millipore-Sigma, #06-570, 1:200, RRID:AB_310177), eGFP (Abcam, #183734, 1:500, RRID:AB_2732027), F4/80 (BioRad-Cl:A3-1 MCA497, 1:100, RRID:AB_2098196), goat anti-GFP (Abcam, ab6673, 1:500, RRID:AB_305643), L-Plastin (LSBio, #LS-C344622, 1:100), P-CSF1R (Cell Signaling, #3154S,1:100, RRID:AB_2085231), ⍺-SMA (Abcam, #5694, 1:100, RRID:AB_2223021).

### Histology and cytochemistry

Following deparaffinization, hematoxylin and eosin or picrosirius red staining (Polysciences, #24,901) were performed following manufacturer protocols. Prior to EdU and TUNEL assays, sections were deparaffinized, and incubated with 0.01 M Sodium Citrate (pH = 6) for 15 min at 95 °C for antigen retrieval. A permeabilization step was followed in which the tissue sections were washed with 1% saponin and APBST wash buffer (APBS supplemented with 0.1% Triton-X100). Slides were then processed for EdU (Invitrogen, #C10337) or TUNEL (Roche #11,684,795,910 or #12,156,792,910) assays according to manufacturer guidelines. Slides were then washed 3 times in PBST for 10 min and nuclear counterstain was achieved by incubating slides with Hoechst 33,342 (Invitrogen, #H3570) or DAPI (Life technologies, #D1306) at 1:1000 dilution. Slides were then washed in PBST 3 times and mounted with fluorescent mounting media (Sigma, #F-4680).

### Microscopy and imaging analysis

Live imaging of larvae, screenings, and time-lapse movies were performed using either a Zeiss Axiovert 200 M inverted microscope or a Leica M205 FCA stereo fluorescence microscope equipped with a Leica DMC 6200 camera. Figure 1A, B images were produced by processing a Z-stack acquisition using the function”Extended Depth of Focus” of LAS X version 3.7.4 software. Confocal images were obtained using Zeiss 700 and 710 Laser Scanning Confocal System (Carl Zeiss, Gottingen, Germany). Z-stack configurations (Fig. 1E: 26 images at 1 µm intervals and 2048 × 2048 size Fig. 1F: 10 images at 1 µm intervals and 1024 × 1024 size) were used to obtain high resolution images using ZEN 2012 Browser (Carl Zeiss, Gottingen, Germany). Fluorescent imaging was performed using a Zeiss Fluorescence Stereomicroscope Axio Zoom.V16 (Carl Zeiss, Oberkochen, Germany). ImageJ was used for image analysis.

### EdU + cell quantification and statistical analysis

To determine whether the number of EdU + cells in proportion to the total number of Hoechst + cells give evidence of a difference between groups, we used Negative Binomial regression on EdU + cell count with the two-level treatment as the predictor (PBS Liposome vs. Clodronate Liposome, Fig. 3E; Clodronate/PBS bead vs. Clodronate/FGF2 bead, Fig. 5B) and the number of Hoechst + cells as the offset [79–81]. This model estimates the ratio of the mean EdU count to the total number of Hoechst + cells for treatment versus control. Note that we used Negative Binomial regression because the measurement of interest is the number of EdU + cells in proportion to the total number of Hoechst + cells. These are counts rather than continuous numeric measurements, so an analysis based upon a count distribution is more appropriate than the more commonly used normal distribution-based procedures like the t-test. We used Negative Binomial regression rather than Poisson regression because there was evidence of overdispersion in the datasets. Because there were 4 eyes assigned to each treatment, we used a total of 8 EdU counts (and the associated Hoechst counts) to fit the model. Note that the Negative Binomial procedure is only approximate, since we have small sample sizes, and that by using the number of Hoechst + cells as an offset, we are conditioning on their value. All animals used for these experiments were post-metamorphotic juveniles at 6–8 months old. For more information on statistical analysis see Additional file 1: Appendix 1.

## Results

### mpeg1:GFP: a new transgenic newt reporter line for macrophages

Macrophage expressed gene 1 (*mpeg1*) has been previously employed for driving fluorescent reporters to label macrophages in several species [46, 82]. Seeking to employ a similar approach for labeling macrophages in *P. waltl*, we created a transgenic line in which the orthologous *mpeg1* promoter drives the expression of an eGFP-encoding sequence. We selected the positive offspring of the founder animals to obtain our first generation (F_1_) of the *P. waltl* transgenic line tgTol2(Dre.*mpeg1:eGFP*)^MHY/SIMON^ (referred as mpeg1:GFP from now on). To test the stability of our transgenic line, we screened all the mpeg1:GFP embryos from generation F_1_ onwards for fluorescent signals. In the positive offspring, eGFP + cells were found spread throughout the body of the animals, likely representing both resident and circulating macrophages, as well as microglia cells in the brain (Fig. 1A, Additional file 3: Video S1). Like macrophages and microglia in other species, morphologically, the eGFP + cells showed a variety of shapes, including spherical, amoeboid, and dendritic (Fig. 1B). Macrophages in many species are notoriously auto fluorescent [83–85]. To ensure that the endogenous eGFP signal was not a result of autofluorescence, we performed immunostainings against GFP. We observed 100% colocalization between endogenous GFP and antibody-derived GFP signal, indicating that the endogenous fluorescence indeed corresponds to the expression of the transgene (Additional file 2: Fig. S1A–C, P). To determine whether macrophages are specifically labelled in different tissues in our mpeg1:GFP transgenic line, we tested the colocalization of eGFP + cells with an established macrophage marker, colony-stimulating factor 1 receptor (CSF1R), in eye tissues. We observed eGFP + cells and CSF1R colocalization in the cornea, vitreous chamber, and iris stroma of newt eyes (Fig. 1C). We then performed immunostainings against two other well-established markers of macrophage populations, F4/80 and L-plastin. F4/80 is a glycoprotein expressed on the cell surface of several subtypes of mature macrophages such as microglia, Langerhans cells, and resident populations in the heart, kidney, and connective tissue [86]. This marker is not expressed on the cell surface of all macrophages and the levels of antigen expression differ depending on the environment where the macrophage is found in mice [86]. In agreement with these studies [86], we observed that 41.4% of eGFP + cells from several tissues in the newt body (tail, trunk, head) were F4/80 + , indicating that this model allows identification of mature macrophages (Additional file 2: Fig. S1 D-I, P). L-plastin is an actin-bundling protein also expressed by macrophages [87]. Notably, we also observed that a significant fraction of the eGFP + cells (21.6%) co-express L-plastin, further indicating that this transgenic model enables labeling of macrophage populations (Additional file 2: Fig. 1J-O, P). Furthermore, we tested in vivo the phagocytic nature of the eGFP + cells by examining the ability of eGFP + cells to phagocytize glucan-encapsulated siRNA particles (GeRPs). GeRPs have been shown to be selectively incorporated by macrophages in several animal models and tissues [49–51]. We first injected GeRPs containing rhodamine into cerebrospinal fluid through intraventricular injection. Right after the injection, we noticed the first particles being approached and encapsulated by eGFP + cells in the central nervous system (Fig. 1D, Additional file 4: Video S2, Additional file 5: Video S3). The tissue analysis 20 h post-injection showed that GeRPs had been phagocytosed by microglia and border associated macrophages along the central nervous system (Fig. 1E). When we injected GeRPs intraperitoneally (Fig. 1D’), we found that, consistent with the previous experiment, GeRPs had been phagocytosed locally by eGFP + cells situated in the intraperitoneal cavity (Fig. 1F). Altogether, these results show that the new mpeg1:GFP *P. waltl* transgenic line labels phagocytic macrophages in vivo.

### Macrophages accumulate transiently in the newt eye after lentectomy

**Fig. 2:**
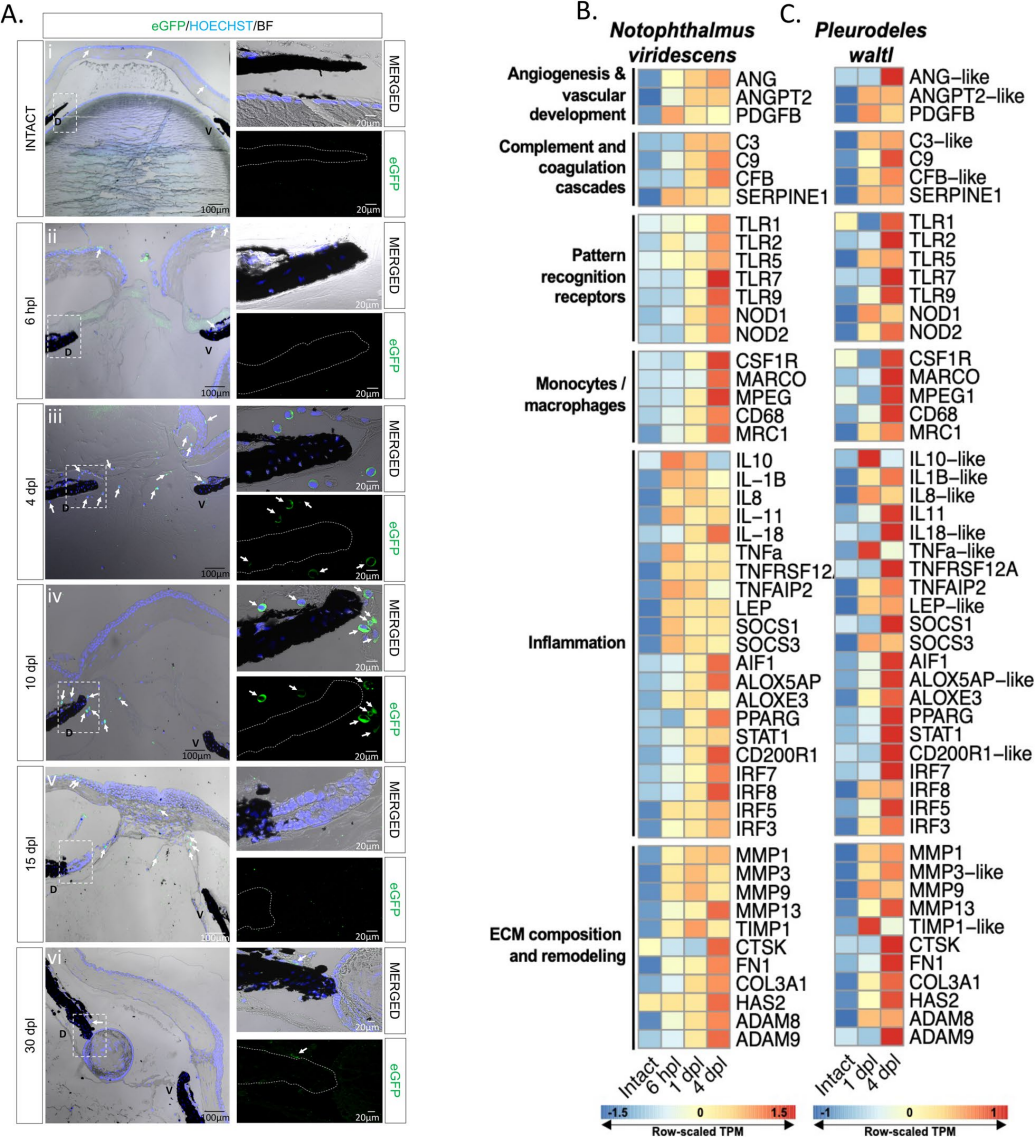
Visualization of macrophage recruitment and identification of highly enriched immune transcripts during lens regeneration. **A** Spatiotemporal view of macrophage recruitment in the newt eye during different stages of lens regeneration. eGFP immunohistochemistry (paraffin embedded tissue) was performed on eyes collected from mpeg1:GFP transgenic newts from intact eyes, 6 hpl (Stage 0) and 4 (Stage 0–I), 10 (Stage I–II), 15 (Stage III–IV) and 30 (Stage VIII) dpl. Macrophages were detected in the cornea of intact eyes and around the dorsal (D) and ventral (V) iris during the early stages of lens regeneration (arrows); n = 6. Scale bars: 100 µm (overviews, left) and 20 µm (insets, right). **B**, **C** Heatmap displays the row-normalized expression levels of select immune-related transcripts in the dorsal iris of *N. viridescens* (**B**) and of *P. waltl* (**C**) following lens removal. The shown transcript identities were assigned by blastx annotation

To characterize the spatiotemporal recruitment of macrophages after lens removal, we analyzed eyes from mpeg1:GFP transgenic animals. Samples were collected prior to lentectomy, at 6 h post-lentectomy (hpl), 4-, 10-, 15-, and 30-dpl. Few eGFP + cells were observed in the intact eyes, with most of them located at the corneal epithelium (Fig. 2A i). At 6 hpl, eGFP + cells were found in the cornea epithelium and inside the blood vessels of the iris (Fig. 2A ii). At this time, the slit that was made in the cornea during the surgical removal of the lens had not closed. By 4 dpl, most macrophages were found around the wound area of the cornea and in the anterior eye chamber near the dorsal and ventral irises (Fig. 2A iii). By 10 dpl, the corneal incisions were closed, and most macrophages were located near the regeneration-competent dorsal iris (Fig. 2A iv). At 15 dpl, once the lens vesicle had formed, macrophages were located in the aqueous chamber (Fig. 2A v). By 30 dpl, very few macrophages were detected in the anterior eye chamber (Fig. 2A vi). In summary, we found that macrophages transiently populate the different anatomical structures of the eye following lens removal, and vanish once the lens is formed, suggesting that macrophages play a role during the early transdifferentiation stages.

### Lens removal triggers a complex early response with a strong immune signature

Wound healing involves distinct phases that are well characterized at the molecular level in mammals. To characterize this highly complex response during newt lens regeneration, we performed bulk RNA sequencing on two newt species (*P. waltl* and *N. viridescens*). We investigated dorsal irises from uninjured eyes, as well as irises following lentectomy at early time points, corresponding to key phases of lens regeneration. Under homeostatic conditions, the vertebrate eye is considered to be an immune-privileged organ due to structural features and immunomodulator mechanisms that act together to limit inflammation [88–90]. As anticipated, prior to lens removal, we observed relatively low expression of immune-related transcripts in both newt species (Fig. 2B, C). Lentectomy triggered a complex immune response, indicated by the upregulation of transcripts involved in inflammation, ECM remodeling, pattern recognition, macrophages/monocytes, vascular development, complement activation and angiogenesis (Fig. 2B, C). Consistent with the macrophage dynamics we observed from the mpeg1:GFP line, RNA sequencing revealed a marked upregulation of macrophage related transcripts at 4 dpl (Fig. 2B, C). A time course of differential expression in *N. viridescens* identified time-dependent regulation of homologs associated with the following KEGG pathways: TNF Signaling Pathway, Toll-Like Receptor Signaling Pathway, Inflammatory Mediator Regulation of TRP Channels, and ECM-Receptor Interaction (Additional file 2: Figure S2). We observed a marked up-regulation of several transcripts exhibiting homology to well-known pro-inflammatory cytokines, such as interleukin-1 beta (IL-1β) and tumor necrosis factor alpha (TNF-a), as well as anti-inflammatory cytokines, such as interleukin-10 (IL-10), between 6 and 24 hpl in both species (Fig. 2B, C, Additional file 2: Figure S2A,C). These observations are consistent with previous reports that show an early upregulation of anti- and pro-inflammatory transcripts in tissues undergoing regeneration [13, 16, 17]. Transcripts encoding proteins that can function as inflammatory mediators, such as CD200R, AIF1, ALOX5AP, ALOXE3, and PPARG, were up-regulated in both species. Transcripts encoding protein products resembling ECM components, such as collagens (COL3A1), fibronectin (FN1), and hyaluronic acid (HAS2), were also upregulated following lens removal (Fig. 2B, C, Additional file 2: Fig. S2D). In addition, we observed the up-regulation of transcripts that exhibited high homology to effectors of ECM remodeling, such as *MMPs*, *ADAMs*, and *TIMP1* (Fig. 2B, C). Our bulk RNA sequencing data paves the way to a better understanding of a genetic response to lentectomy that triggers lens regeneration in newts.

### Macrophage depletion inhibits lens regeneration and affects cell cycle re-entry of iPECs

**Fig. 3:**
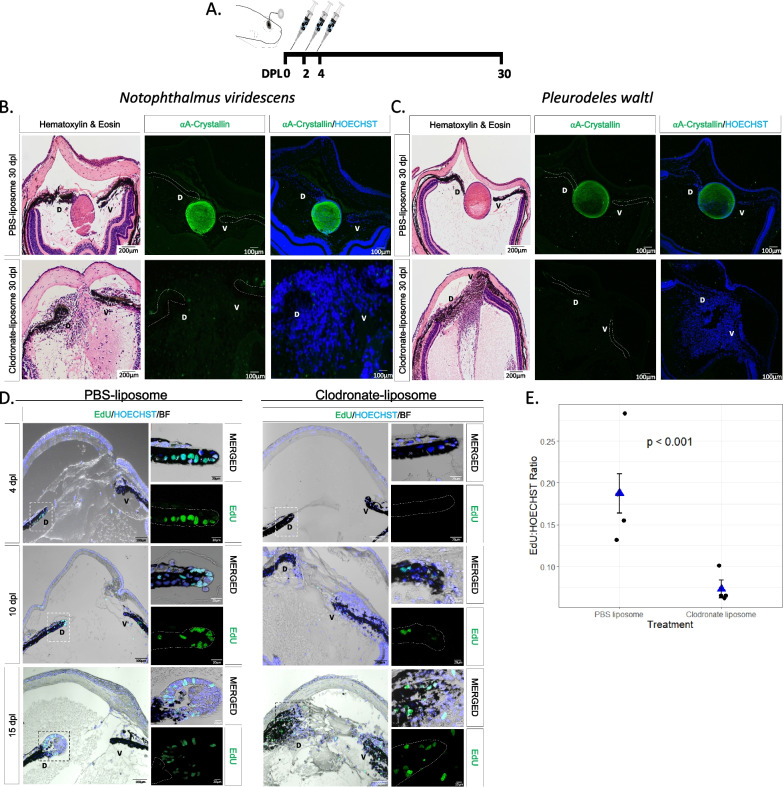
Early macrophage depletion inhibits lens regeneration and inhibits cell cycle re-entry of iPECs. **A** Schematic representation of experimental design. PBS or clodronate liposomes were administered intraocularly following lentectomy at 0, 2 and 4 dpl. **B** Lens regeneration in *N. viridescens* was inhibited following early macrophage depletion indicated by the absence of ⍺A-Crystallin staining (paraffin embedded tissue). In addition, cellular accumulation was observed in the eye cavity at 30 dpl (Stage VIII); n = 40. **C** *P. waltl* displayed a similar phenotype following intraocular administration of clodronate liposomes; n = 40. Scale bars: 200 µm (Hematoxylin & Eosin, left) and 100 µm (immunostainings, right) (paraffin embedded tissue); the dorsal (D) and ventral (V) iris epithelium are marked. **D** EdU assay was performed on eyes collected at 4 (Stage I), 10 (Stage II–III) and 15 (Stage IV–V) dpl following PBS and clodronate treatment (paraffin embedded tissue). The dorsal (D) and ventral (V) iris epithelium are marked. Inset images of the dorsal iPECs highlight the effects of macrophage depletion on cell cycle re-entry; n = 4 per time point. Scale bars: 100 µm (overviews, left) and 20 µm (insets, right). **E** Quantification of differences in the ratio of cells entering the S phase between clodronate and PBS treatment at 4 dpl; n = 4. We estimated that the ratio of EdU to Hoechst cells was 0.187 (standard error 0.023) for the PBS liposome condition, while the ratio was 0.073 (standard error 0.011) for the clodronate liposome condition. Negative binomial regression, *p* < 0.001

To test the role of macrophages during the early stages of lens regeneration, we injected control or clodronate liposomes into the eyes of the two newt species (Fig. 3A). Liposomes are phagocytized by macrophages and, if carrying clodronate, ultimately induce apoptosis [91, 92]. Lens regeneration was evident by 30 dpl in control-treated animals, but not in macrophage-depleted eyes, as indicated by the absence of a lens and ⍺A-Crystallin (a lens-specific marker) in both species (Fig. 3B,C). In addition, histological assessment revealed several morphological and cellular abnormalities in the eye and near the dorsal and ventral aspects of the iris. Furthermore, an unusual cellular accumulation in the anterior and posterior eye chamber was observed, resembling the formation of scar-like tissue (Fig. 3B, C). The inhibition of lens regeneration, morphological alterations, and cellular accumulation phenotypes were observed in 100% of the cases tested (n = 40 per species). These data suggest that macrophages are essential to achieve lens regeneration in newts, as their depletion leads to the formation of scar-like tissue instead of the formation of a new lens.

Next, we sought to explore potential mechanisms by which macrophage depletion could inhibit lens regeneration. Previous work suggested that macrophage depletion affects the survival of progenitor cells during fin regeneration in zebrafish [93]. We tested if similar mechanisms take place during newt lens regeneration by TUNEL staining. We found that macrophage depletion using clodronate liposomes did not lead to apoptosis of iPECs in the early stages of lens regeneration (up to 10 dpl; Additional file 2: Figure S3). As reported previously, apoptotic nuclei were observed at basal levels in transdifferentiating lens epithelial cells (LECs) in control-treated animals once a lens vesicle is formed (Additional file 2: Figure S3) [94].

Since apoptosis of iPECs was not evident at the early stage, we next investigated whether cell cycle re-entry is affected upon macrophage depletion by analyzing EdU incorporation in the lentectomized irises in *P. waltl*. Cell cycle re-entry of the terminally differentiated iPECs is one of the most prominent events after lentectomy [45]. We observed that macrophage depletion changed the dynamics of cell cycle re-entry of iPECs. As expected in control treated eyes, the iPECs of the dorsal iris re-entered the cell cycle at 4dpl as indicated by EdU + staining (Fig. 3D). On the other hand, upon macrophage depletion, iPECs failed to re-enter the cell cycle as indicated by the lack of EdU staining inside the iris epithelium (Fig. 3D). During later stages in control eyes, lens epithelial and lens fiber cells located inside the lens vesicle were EdU + at 10 dpl and 15 dpl respectively, whereas in clodronate liposome treated eyes, a lens vesicle failed to form, and EdU + cells were detected inside the iris and inside the vitreous and aqueous chambers (Fig. 3D). Based on a Negative Binomial Regression analysis, we found strong evidence of differences (*p* < 0.05) in the log ratio of mean EdU + cells to HOECHST + cells at 4 dpl (Fig. 3E). Note that we provide a justification in the Methodology section for use of Negative Binomial regression instead of a t-test; the t-test based analysis also yields *p* < 0.05. Furthermore, we noticed that the regenerating lens appears bigger and more developed at 15 dpl in control-treated animals compared with the mpeg1:GFP animals at the same time (compare PBS-liposome group, 15dpl in Fig. 3D, where animals were 6 months old, with 15dpl in Fig. 2A, where animals were 3 years old). This observation is consistent with our previous study showing that lens regeneration is delayed in older *P. waltl* [44]. Our data support that, while macrophage depletion does not cause an apoptotic response during the early timepoints evaluated in this study, macrophages do play a role in the induction of cell cycle re-entry of iPECs.

### Macrophage inhibition prolongs inflammation, alters ECM remodeling, and causes a fibrotic-like response

**Fig. 4:**
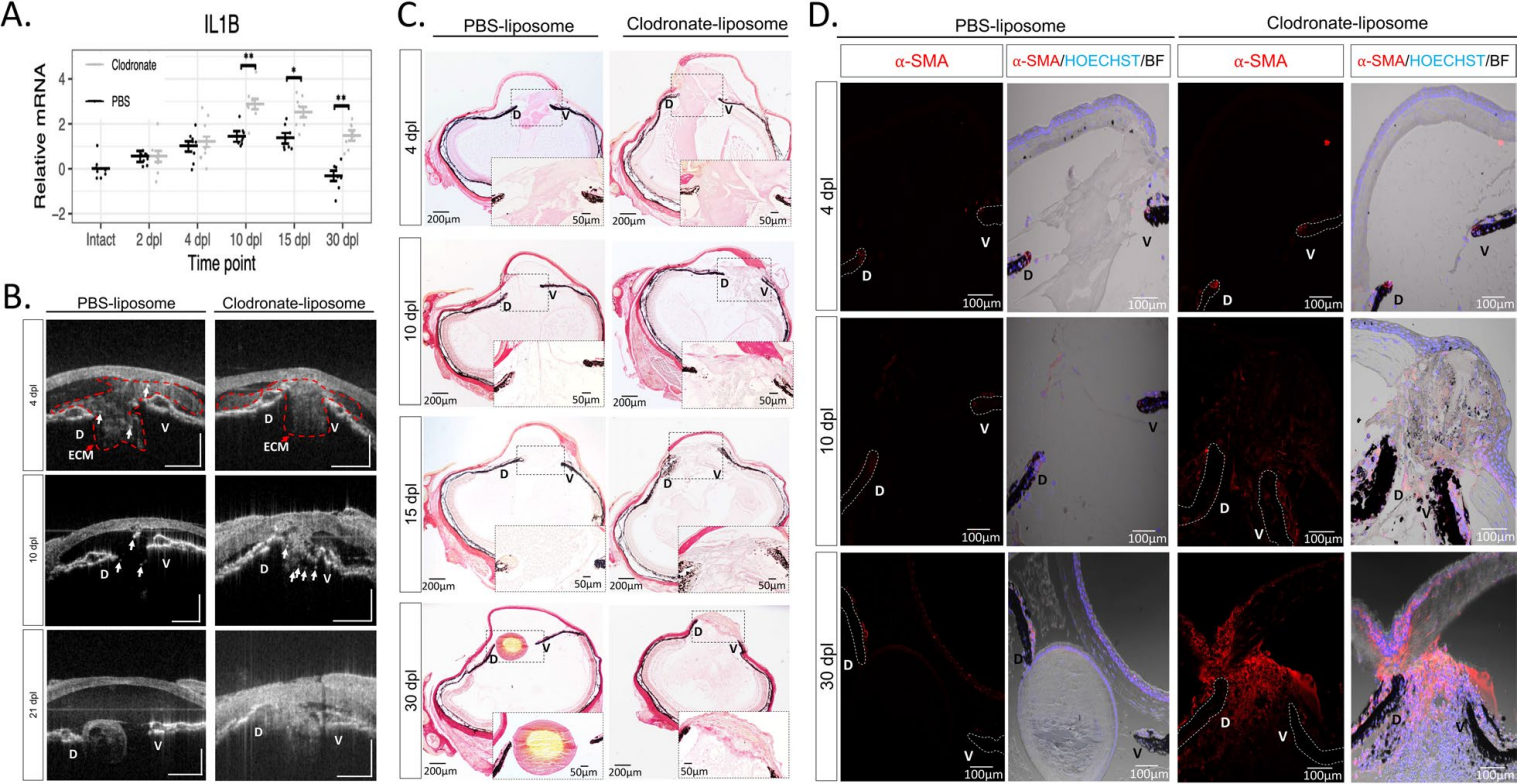
Early macrophage inhibition prolongs inflammation and disrupts ECM remodeling. **A** RT-qPCR analysis revealed an upregulation of IL-1β expression in clodronate-treated eyes at 10, 15, and 30 dpl; 8 eyes per treatment at each time point, *adjusted *p* < 0.005, **adjusted *p* < 0.0001. Error bars in plots represent standard error of mean estimate. Estimates were determined as described in methods to account for batch effects. Transcript abundance in the intact eye is shown for reference but was not included in the statistical analysis (see methods, Additional file 1: Appendix 1). **B** In vivo imaging of lens regeneration with SD-OCT shows the kinetics of ECM clearing in PBS liposome treated eyes. Following macrophage depletion, ECM remodeling was altered, and regeneration was inhibited; n = 10. Arrows point to cloudy opacity in the SD-OCT image that is interpreted as ECM accumulation. **C** Deposition of collagen fibers were visualized with picrosirius red staining (Collagen stain red/pink in brightfield images) (paraffin embedded tissue). Collagen fibers were detected in the vitreous and aqueous chambers of PBS treated eyes at 4 dpl (Stage I) but cleared out by 10 dpl (Stage II-III); n = 6. A progressive increase in collagen staining intensity was observed in clodronate treated eyes. Inset images show a higher magnification of the aqueous chamber. **D** The absence of macrophages triggers a fibrotic-like response. Myofibroblast presence was noted in the vitreous and aqueous chambers of macrophage depleted eyes at 10 (Stage II-III) and 30 dpl (Stage VIII), as indicated by ⍺-SMA staining; n = 6. Scale bars: 100 µm (paraffin embedded tissue)

To characterize the effects of macrophage depletion on the ensuing inflammatory response after lentectomy, we performed RT-qPCR to measure changes in the expression of the pro-inflammatory cytokine, IL-1β. Consistent with our bulk RNAseq experiments (Fig. 2B, C), *IL-1β* expression was upregulated after lentectomy and returned to basal levels by 30dpl in control eyes (Fig. 4A). However, in clodronate-treated eyes, IL-1β expression was significantly higher than controls at 10 dpl and remained higher than controls even at 30 dpl (Fig. 4A).

We then used SD-OCT to image the morphological changes that accompany macrophage depletion. The non-invasive nature of SD-OCT allowed us to monitor the lens regeneration process from the same newt in real time [78]. Using SD-OCT, we recently demonstrated that ECM remodeling is dynamic and highly orchestrated during lens regeneration [44]. At 4 dpl, a cloudy opacity was observed in the anterior eye chamber between the dorsal and ventral iris of both PBS- and clodronate-treated eyes, indicating an accumulation of ECM (Fig. 4B, Additional file 6: Video S4). By 10 dpl, the opacity was cleared from the anterior chamber of control eyes, and a newly formed lens vesicle was observed at the dorsal iris by 21 dpl (Fig. 4B, Additional file 7: Video S5 and Additional file 8: Video S6). In contrast, by 10 dpl, ECM not only failed to clear, but progressively worsened by 21 dpl from macrophage-depleted eyes (Fig. 4B, Additional file 9: Video S7, Additional file 10: Video S8 and Additional file 11: Video S9). Several other morphological abnormalities were detected in the newt eye by 21 dpl. The aqueous humor that fills the area between the cornea and iris failed to reform in clodronate-treated eyes, and bright spots were observed in the vitreous and around the dorsal and ventral irises (Fig. 4B). We interpreted the bright spots as the cellular accumulation shown in Fig. 3B, C. To confirm our interpretation of the SD-OCT findings, we used histology and picrosirius red staining to detect collagen fibers, a major component of the ECM [44]. Collagen accumulation appeared to be larger and denser in the anterior chamber of clodronate-treated eyes compared to control eyes at 4 dpl (Fig. 4C). In agreement with our SD-OCT interpretations, we observed that collagen fibers started to clear out at 10 dpl in control eyes and, by 30 dpl, very few collagen fibers were detected in the eye chambers. In contrast, collagen staining remained at 30 dpl in clodronate-treated eyes (Fig. 4C). Furthermore, we found that at least some of these cells are myofibroblasts via alpha smooth muscle actin (⍺-SMA) staining (Fig. 4D). Myofibroblasts are known to be involved in ECM remodeling, immune modulation, and angiogenesis [95]. To evaluate the kinetics of myofibroblast activation and accumulation in the anterior eye chamber, we performed immunostaining with ⍺-SMA at 4, 10, and 30 dpl in *P. waltl*. While at 4 dpl we detected very few ⍺-SMA + cells in both experimental conditions, by 10 dpl, the accumulation of ⍺-SMA + cells was evident only in the anterior chamber of macrophage-depleted eyes, and this phenomenon was exacerbated by 30 dpl. In contrast, newts treated with control liposomes displayed substantially lower ⍺-SMA reactivity at 30 dpl relative to clodronate treatment (Fig. 4D). Collectively, SD-OCT and histology demonstrate significant abnormalities following macrophage depletion in the newt eye, including cellular accumulation and lack of ECM clearance, which result in the formation of scar-like tissue instead of lens regeneration.

### Exogenous FGF2 can rescue lens regeneration processes in macrophage-depleted eyes

**Fig. 5:**
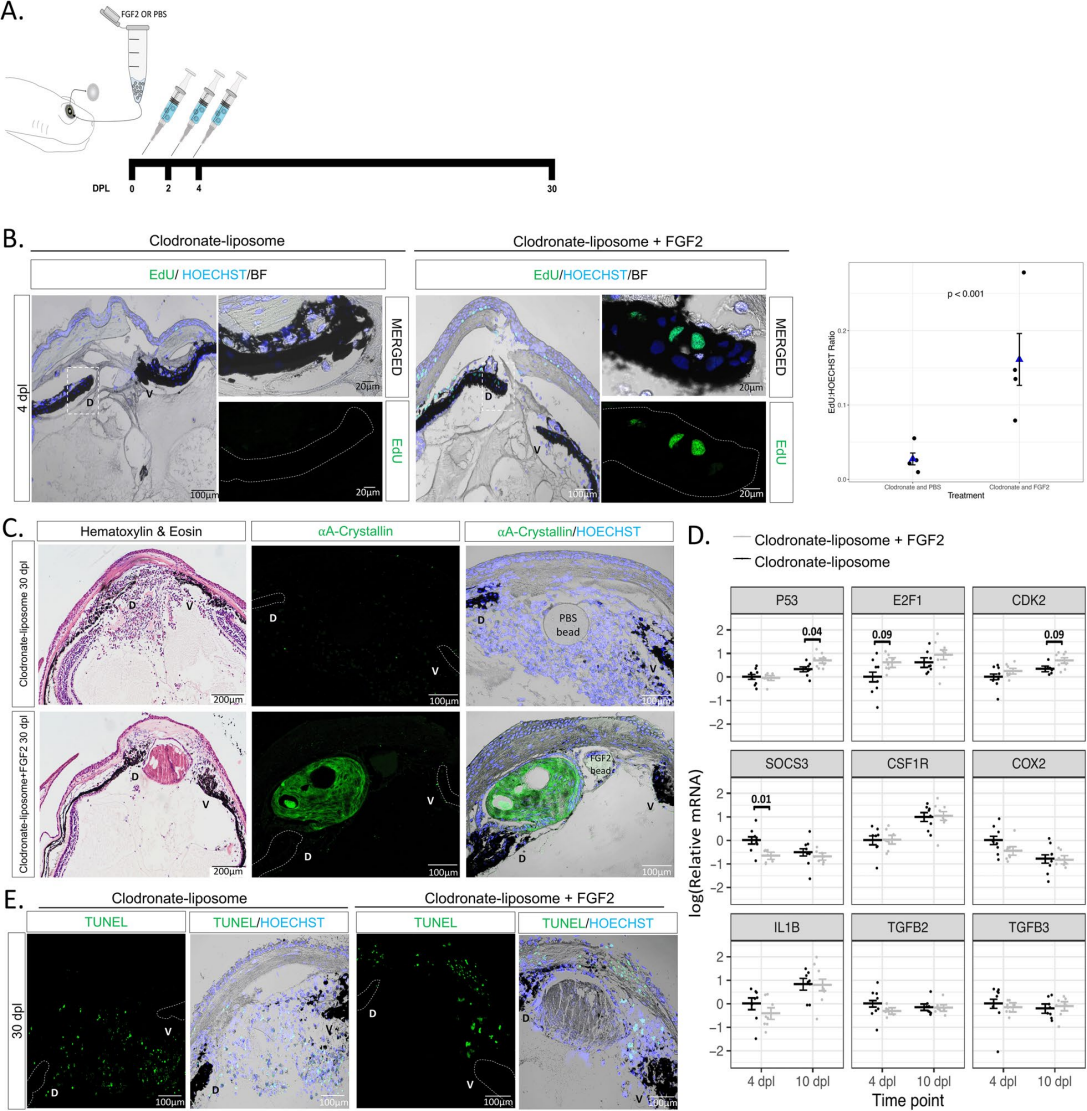
Exogenous FGF2 rescues cell cycle re-entry and lens regeneration following early macrophage depletion. **A** Schematic representation of experimental design. Clodronate liposomes were administered intraocularly following lentectomy at 0, 2 and 4 dpl, and a heparin bead incubated with PBS or FGF2 was added into the newt eye at 0 dpl. **B** EdU staining (paraffin embedded tissue) and quantification revealed a significant increase (*p* < 0.001) in iPECs at the S-phase of the cell cycle in clodronate and FGF2-treated eyes; n = 4. We estimated that the ratio of EdU to Hoeschst was 0.161 (standard error 0.035) for the clodronate and FGF2 condition, while 0.028 (standard error 0.008) for the clodronate and PBS condition. Scale bars: 100 µm (overviews, left) and 20 µm (insets, right). Negative binomial regression, *p* < 0.001. **C** Histology and immunostaining (paraffin embedded tissue) for lens specific marker ⍺A-Crystallin revealed that exogenous supplementation of FGF2 induced lens regeneration and resolve cellular accumulation at 20 dpl in 4/12 eyes. On the contrary, none of the 12 clodronate treated eyes that were supplemented with PBS beads had a crystallin lens; n = 12. Scale bars: 200 µm (Hematoxylin & Eosin, left) and 100 µm (immunostainings, right). **D** RT-qPCR analysis for genes involved in cell cycle and inflammation at 4 and 10 dpl; 8 eyes per treatment at each time point. Statistical analysis using two-way ANOVA was performed and the adjusted *p* values displayed for *p* < 0.1. Error bars in RT-qPCRs plots represent standard error of mean estimate. Estimates were determined as described in methods to account for batch effects. **E** Detection of apoptosis in clodronate treated eyes with and without FGF2 supplementation. TUNEL + nuclei were observed in the cornea and near the ventral (V) iris; n = 12. Scale bars: 100 µm (paraffin embedded tissue)

Previous studies have shown that FGF signaling pathway plays an important role during lens regeneration [96–100]. Since macrophages can directly secrete FGF in certain conditions [101, 102], we hypothesized that macrophage depletion could affect the percentage of iPECs re-entering the cycle by affecting the FGF levels in the newt eye. To test this hypothesis, we administrated exogenous FGF2 into the newt eye right after lentectomy, followed by clodronate treatment (Fig. 5A). We observed that FGF2 treatment caused a significant increase (*p* < 0.001) in the overall number of EdU + iPECs at 4 dpl relative to clodronate treatment only (Fig. 5B). Astonishingly, by 30 dpl, a lens vesicle was observed in one third of the cases, stemming from the dorsal iris of FGF2-supplemented eyes following clodronate treatment (4/12 eyes) (Fig. 5C). In addition to rescuing regeneration, clodronate and FGF2 co-administration caused a reduction in the generalized cellular accumulation, as indicated by the lack of nuclear staining compared to clodronate and PBS co-administration (Fig. 5C). Our data show that exogenous administration of FGF2 in macrophage-depleted eyes can rescue lens regeneration and modulate the accumulation of scar-like tissue.

We next evaluated the gene expression levels of several inflammatory and cell cycle-related genes via RT-qPCR analysis at 4 and 10 dpl. The expression levels of cell cycle associated gene (P53) was found to be significantly upregulated in clodronate/FGF2-treated eyes compared to clodronate/PBS-treated eyes at 10 dpl. In addition, we observed moderate evidence (adjusted *p* < 0.1) that *E2F1* and *CDK2* were upregulated at 4 dpl and 10 dpl respectively in the FGF2-treated eyes. Furthermore, the expression of the suppressor of cytokine signaling 3 (*SOCS3*) gene was significantly downregulated at 4 dpl in clodronate/FGF2-treated eyes. In contrast, we did not find evidence for changes in the expression patterns of macrophage specific receptor *CSF1R* or inflammatory agents *COX2*, *IL-1β*, *TGFβ2*, and *TGFβ3* in control and FGF2-treated eyes (Fig. 5D). We then tested if supplementing FGF2 in macrophage depleted eyes affected the apoptotic levels at 30 dpl. We found that apoptotic cells were still evident in the aqueous chamber near the regenerating lens and inside the cornea (Fig. 5E). Our results suggest that FGF2 plays an important role during iPECs cell cycle re-entry and supplementation of exogenous FGF2 is sufficient to start the regeneration process, even in the absence of macrophages.

### Late administration of clodronate enhances apoptosis and pro-inflammatory signals

**Fig. 6:**
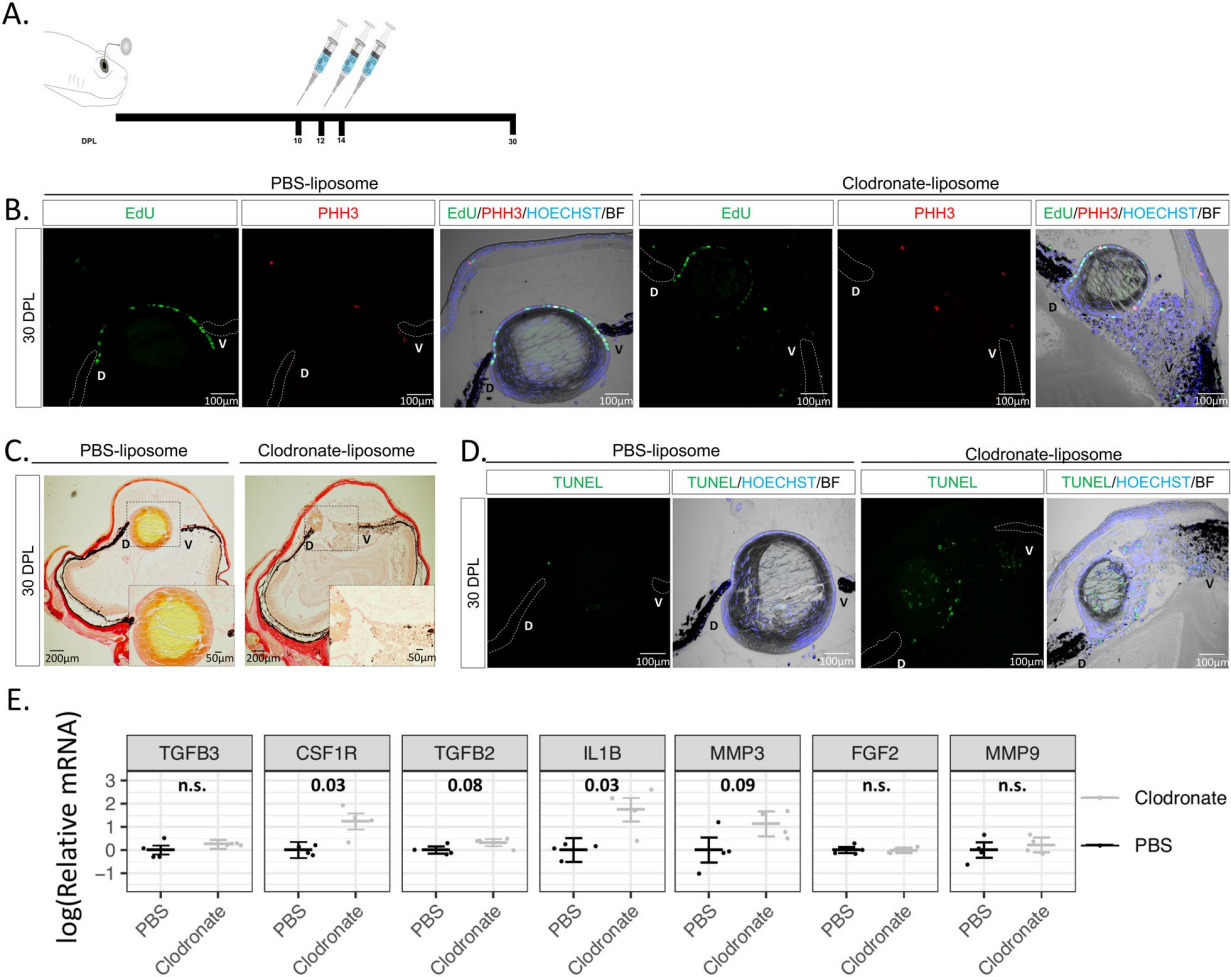
Late macrophage depletion after lens vesicle formation enhances apoptosis and pro-inflammatory signals. **A** Schematic representation of experimental design. Clodronate or PBS liposomes were injected intraocularly in the aqueous chamber at 10, 12, and 14 dpl after lens vesicle formation. **B** LEC in both treatments were positive for EdU and mitosis marker PHH3; n = 6. Scale bars: 100 µm (paraffin embedded tissue). **C** Collagen staining was more abundant in the vitreous chamber of the clodronate treated eyes; n = 6. Inset images show higher magnification of the aqueous and vitreous chambers (paraffin embedded tissue). **D** Apoptotic cells were detected in the aqueous chamber and inside the regenerating lens of clodronate liposome-treated eyes; n = 6. Scale bars: 100 µm (paraffin embedded tissue). **E** RT-qPCR analysis revealed an upregulation of the anti-inflammatory gene IL1-β and macrophage receptor CSF1R following late clodronate administration; 4 eyes per treatment. Statistical analysis using Welch's two-sample t-test was performed and adjusted *p* values displayed for *p* < 0.1. Error bars in plots represent standard error of mean estimate. n.s., Not significant (See Additional file 1: Appendix 1)

To examine if newt macrophages play a role during the later stages of lens regeneration after the critical window of lens vesicle formation has passed, we treated eyes with either clodronate or PBS liposomes at 10, 12, and 14 dpl (Fig. 6A). Even though a lens was detected by 30 dpl, and LECs were positive for EdU and phospho-histone H3 (PHH3), the lens appeared smaller in all clodronate-treated eyes (Fig. 6B). In addition to abnormal lens morphology (smaller size, presence of cells without fiber characteristics in the lens cortex and a multilayer lens epithelium), severe cellular accumulation was observed between the lens and the ventral iris (Fig. 6B, C). Collagen fiber staining revealed that ECM accumulation was evident in the vitreous and aqueous chambers following clodronate treatments (Fig. 6C). Furthermore, TUNEL + nuclei were observed inside the lens fibers and near the ventral iris in clodronate treated eyes, indicating that macrophages may play a role in preventing cell death during the late phases of lens regeneration (Fig. 6D). Similar observations were noted when clodronate administration was initiated at 20 dpl (Additional file 2: Figure S4). To complement, we tested for the expression of selected immune and inflammation related targets via RT-qPCR and found that late clodronate administration caused an increase in expression of the pro-inflammatory cytokine *IL-1β* and the macrophage-specific receptor *CSF1R* at 30 dpl (Fig. 6E). Expression patterns of *FGF2*, *TGFB2*, *TGFB3*, *MMP3*, and *MMP9* were not significantly changed (Fig. 6E). Altogether, these data suggest that macrophages are also necessary for the proper regeneration of the lens in the later phases of the process, potentially controlling survival of lens cells and resolving pro-inflammatory signals.

### A new injury helps to resolve the fibrotic phenotype and can re-activate lens regeneration

**Fig. 7:**
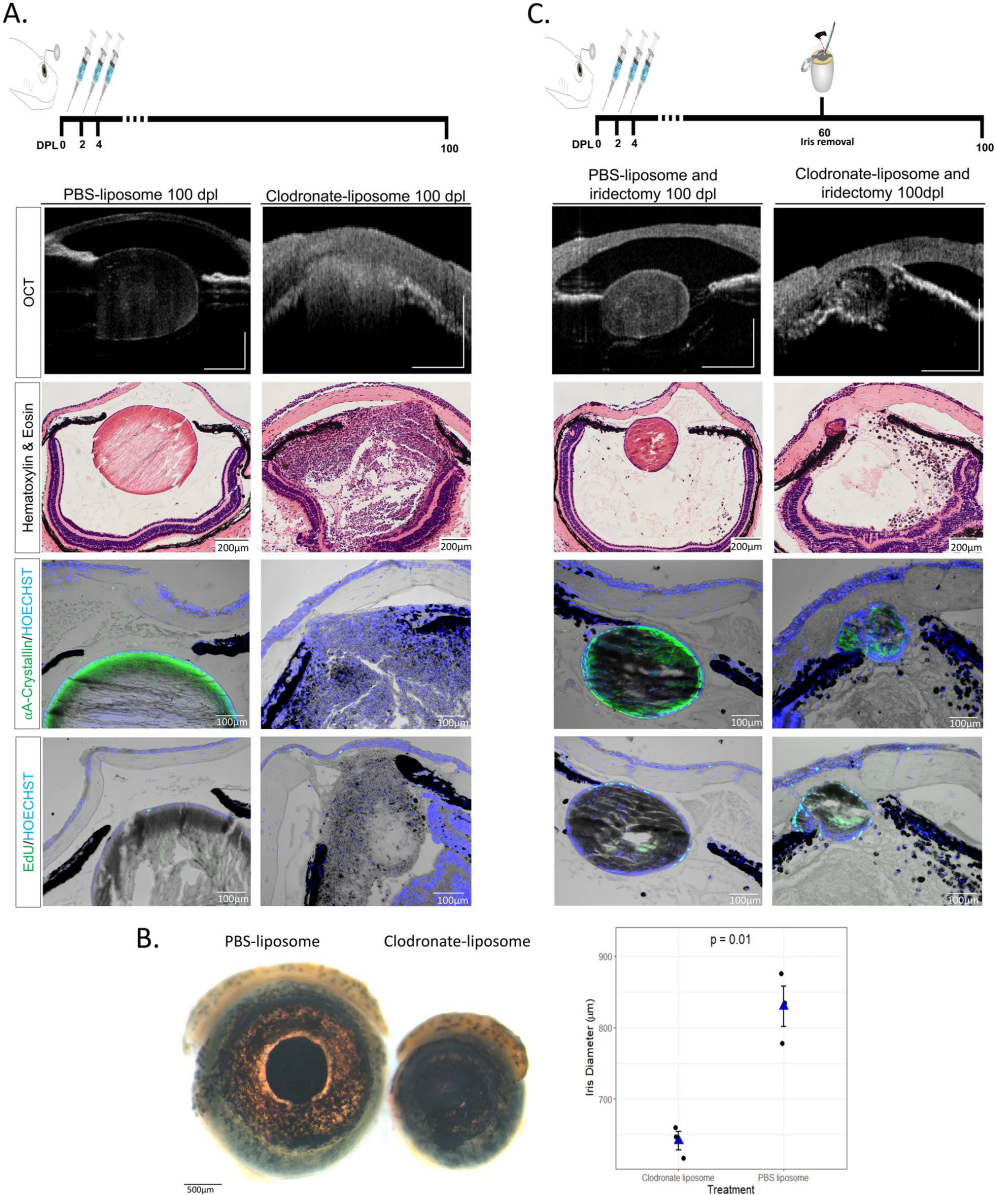
A secondary injury can induce the reabsorption of the scar-like tissue and re-initiate lens regeneration. **A** Schematic representation of the experimental design. Clodronate or PBS liposomes were administered at 0, 2 and 4 dpl. At 100 dpl (Stage IX), 10/10 clodronate liposome treated eyes showed severe cellular accumulation, and ECM (cloudy opacity) in the aqueous and vitreous chambers. Furthermore, no lens was observed in any of the clodronate liposome-treated eyes as indicated by the absence of lens specific marker ⍺A-Crystallin, and no EdU + cells were observed in the lens (paraffin embedded tissue). On the contrary, 10/10 of the PBS liposome-treated eyes regenerated a crystallin lens with EdU LECs without signs of ECM or cellular accumulation; n = 10 per treatment. Scale bars from top to bottom: 100 µm (OCT), 200 µm (Hematoxylin & Eosin) and 100 µm (immunostainings,). **B** At 100 dpl, microphthalmia was observed in clodronate liposome-treated eyes. Scale bar 500 µm. Statistical analysis using Welch's two-sample t-test was performed and adjusted *p* values displayed for *p* < 0.1 (See Additional file 1: Appendix 1). Dots represent the average diameter of animals' eyes (n = 3 surviving animals in each condition). **C** Schematic representation of experimental design. Iridectomy was performed at 60 dpl and eyes were collected for histological analysis at 100 dpl. Secondary injury in the iris restarted the lens regeneration process, resolved cellular and ECM accumulation in 3/8 clodronate liposome-treated and iridectomized eyes, as indicated by SD-OCT, histology, ⍺A-Crystallin and EdU staining at 100 dpl (40 days post-secondary injury); n = 8 (paraffin embedded tissue). Regeneration was restarted in all PBS liposome-treated eyes; n = 8. Scale bars from top to bottom: 100 µm (OCT), 200 µm (Hematoxylin & Eosin) and 100 µm (immunostainings)

The results observed above led us to believe that macrophages in newts were playing an anti-fibrotic role during the injury response. This made us question whether macrophages could resolve the fibrotic injury that was triggered by their absence, if given sufficient time to recover in number following clodronate depletion and lentectomy. To test this, we treated eyes with clodronate or PBS liposomes at 0, 2, and 4 dpl and monitored the animals using SD-OCT for 100 days, as well as H&E, EdU staining, and ⍺A-Crystallin immunohistochemistry. We found no evidence of proliferation or lens formation at 100 dpl in all cases that were treated with clodronate (n = 10) (Fig. 7A). Startlingly, we also observed a microphthalmic phenotype in 5/10 eyes that were treated with clodronate (Fig. 7B).

We hypothesized that once the effects of clodronate diminished and macrophages returned to the eye chamber, they would be met with an inflammatory microenvironment that would immediately polarize them to a damaging, pro-inflammatory phenotypic response. Therefore, we wanted to test if a second injury was sufficient to reprogram the macrophages in order to resolve the chronic inflammation and fibrosis. To do that, we surgically removed a piece of the dorsal iris (iridectomy) at 60 dpl in control- and clodronate-treated eyes without removing the fibrotic mass that was present in the anterior eye chamber (Fig. 7C). It was previously shown that following iridectomy, the iPECs proliferate to replace the missing tissue, and a lens is formed by the transdifferentiation of the newly formed iPECs [103]. Similar to these observations, we detected a newly formed lens following iridectomy in control PBS-treated eyes (Fig. 7C). Interestingly, we found that in 3/8 cases where iridectomy was performed in clodronate-treated eyes, regeneration was initiated, indicated by the presence of an ⍺A-Crystallin + lens vesicle at the dorsal iris (Fig. 7C). Importantly, the cellular accumulation and fibrotic phenotype was mostly resolved from the anterior chamber in the cases that lens regeneration was induced (Fig. 7C).

## Discussion

While it is generally accepted that macrophages play a significant role in tissue repair and regeneration, there is ongoing debate about the precise mechanisms and factors involved. Research in a variety of model organisms have shown that macrophages can both promote and inhibit scar formation, depending on their phenotype and the stage of the repair process [104, 105]. This involvement of macrophages is essential for understanding tissue regeneration mechanisms, as differently polarized macrophages express specific pro- and anti-inflammatory cytokines, influencing multiple processes such as direct and indirect roles in ECM remodeling, vascular inflammation, repair, chemotaxis, recruitment of various cell types, phagocytosis, pro-angiogenesis signaling networks, and physical interactions with endothelial cells [106, 107].

Studying the role of macrophages in regenerative contexts on different model organisms can shed light into the details of how macrophages can control different healing trajectories (regeneration vs. scar formation). The knowledge gained understanding the physiological regeneration process in regenerative species could one day be used to influence the cellular fate and pathophysiology in non-regenerative species. For example, using two species of mice (*Acomys and Mus*), Simkin and collaborators identified secreted factors from activated *Acomys* macrophages that induce a pro-regenerative phenotype in fibroblasts from both species, demonstrating that cell-autonomous mechanisms govern how macrophages react to the same stimuli to differentially produce factors that facilitate regeneration [108].

Despite being the animals with the highest regenerative capacity of the tetrapod lineage, the immunology field in salamander regeneration research is still in its infancy, with just a few studies exploring the role of macrophages in limb regeneration of the axolotl [17, 21, 25]. In this study, we used the lens regeneration paradigm in two newt species to build upon our knowledge of the macrophage’s role during pro-regenerative responses and scar formation.

We present here a transgenic newt line, * mpeg1:GFP*, that opens new avenues for in vivo tracking studies and will enable cross species comparisons of transcriptional profiles at the single cell level. A similar zebrafish reporter line [46] has been recently used to characterize at the cellular levels pro-inflammatory phagocytic macrophages and pro-remodeling macrophages with tissue regeneration signatures [109]. The presence of macrophages had been described in *Notophthalmus* up to 20 days using classical techniques, such as light and electron microscopy [40], but their function required further investigation [110]. Using our mpeg1:GFP line, we provide the first spatial description of the transient occupation of macrophages on different eye structures along the course of 30 days during lens regeneration in *P.  waltl*.

We show for the first time the complex and dynamic immune signature of early responses to lentectomy in these two newt species, with upregulation of transcripts involved in inflammation, ECM remodeling, pattern recognition, macrophages/monocytes, vascular development, complement activation, and angiogenesis. Both in acute and chronic wound healing of multiple tissues and species, macrophages display direct and indirect roles in hypertrophic scar formation versus scarless repair, as they can modulate fibroblast proliferation, myofibroblast differentiation and remodeling processes such as collagen deposition [93, 111–114]. The early upregulation of anti- and pro-inflammatory transcripts is similar to that reported in other tissues in regeneration-competent amphibian species [13, 16, 17].

To expand beyond simply describing the presence of macrophages during lens regeneration, which was first characterized over six decades ago [38–41, 110], we also show the consequences of macrophage depletion during lens regeneration in newts. Using a similar macrophage depletion approach, macrophages were found to be required for limb regeneration in another salamander species, the axolotl [17]. Here we show that, like in the axolotl limb [17], macrophages are essential to achieve lens regeneration in newts, as their depletion leads to the formation of scar-like tissue instead of the formation of a new lens. There is increasing evidence that the innate immune system, and macrophages or macrophage-like cells, interact with and modulate other cells during regeneration to regulate processes like stem cell behavior and cell competition [115]. In the case of newt lens regeneration, we observed changes in the rate of cell cycle re-entry and proliferation of iPECs, but we did not find evidence of apoptosis in dorsal iPECs at the early stages of regeneration. We used non-invasive SD-OCT to monitor the lens regeneration process from the same newts in real-time [44, 78], and recorded the dynamic formation of scar tissue following macrophage depletion. We describe here a clodronate-induced accumulation of both collagen and myofibroblasts. Based on studies in other model organisms, newt macrophages could be directly impacting extracellular matrix remodeling [19, 102, 116–119]. Our findings suggest that under normal conditions in the newt eye, macrophages play a critical role in preventing myofibroblast accumulation and modulating ECM remodeling. Future studies will need to characterize the phenotypic responses of macrophages associated with these pro-wound healing and anti-fibrotic responses. Myofibroblasts are known to be involved in ECM remodeling, immune modulation, and angiogenesis [95]. However, in zebrafish, macrophages have been found to directly contribute collagen to scar formation during tissue repair processes, challenging previous beliefs about the exclusive role of myofibroblasts in collagen deposition [117]. Whether the accumulation of collagen found in newts after early depletion of macrophages by clodronate is secreted by myofibroblasts, late macrophages, or both requires further investigation.

**Fig. 8:**
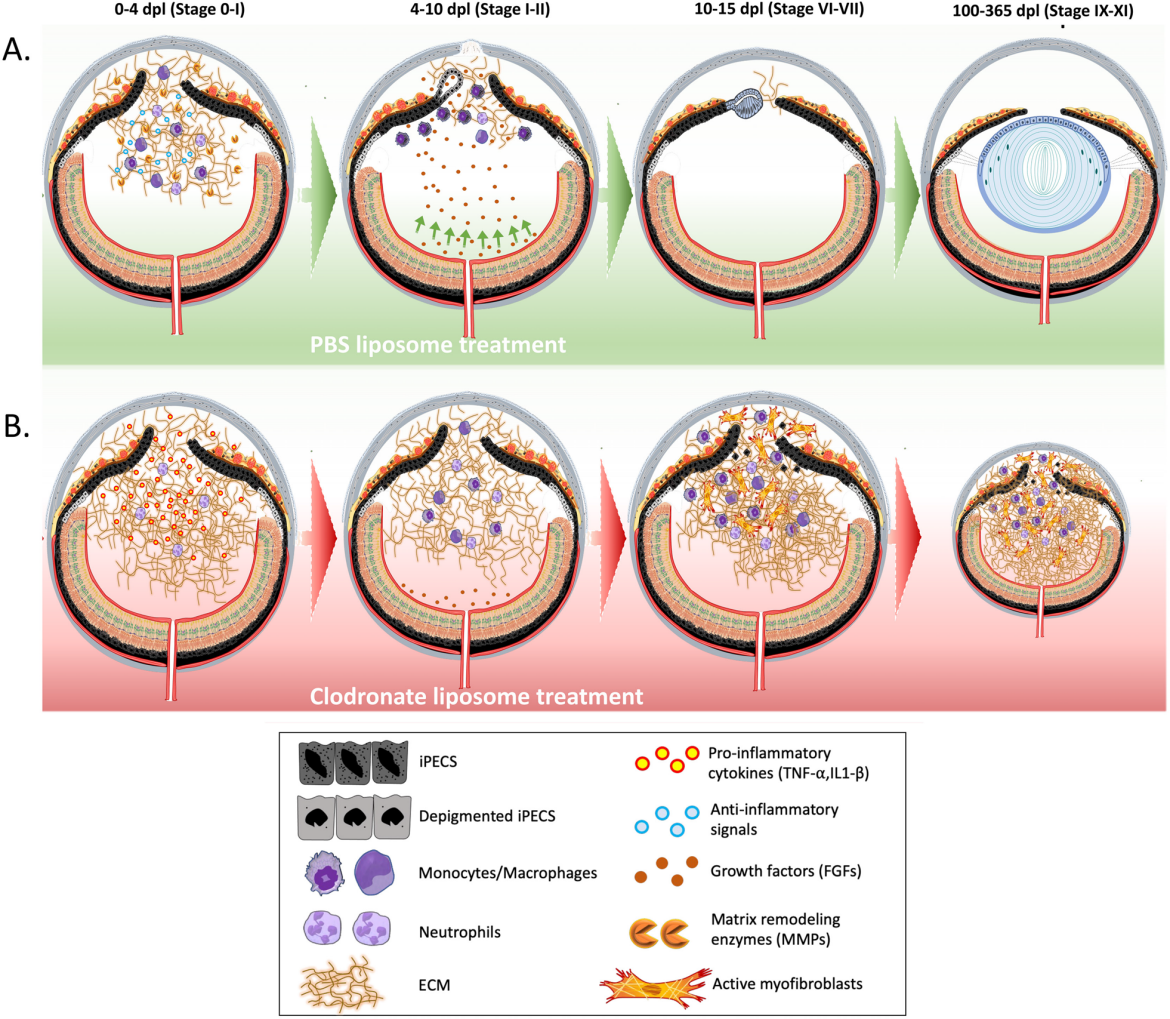
Simplified overview of the proposed model. **A** Following lentectomy, pro-inflammatory cytokines are secreted into the eye cavity and the anterior chamber of the newt eye fills with ECM. Macrophages and other immune cells accumulate in the eye by 4 dpl and secrete anti-inflammatory signals and matrix remodeling molecules to resolve inflammation and degrade ECM. Once ECM is cleared out, growth factors that are secreted from the neural retina reach the iPECs which then enter the cell cycle and start to dedifferentiate. During this phase, macrophages phagocytize the melanosomes that are discharged by iPECs [35, 38, 40]. Dorsal iPECs become completely depigmented and give rise to a new lens vesicle. As LECs proliferate and differentiate into lens fibers, the regenerating lens becomes larger. **B** Upon clodronate treatment, the lack of anti-inflammatory cytokines and matrix remodeling molecules caused by the absence of macrophages at 4dpl, results in an increased and prolonged inflammatory state and the exacerbation of matrix accumulation. As a result, growth factors secreted from the retina can’t traffic through the damaged eye chamber and iPECs fail to re-enter the cell cycle. Alternatively, the macrophages are directly modulating the cell cycle re-entry of the iPECs. The pathogenic environment triggers the recruitment, differentiation, and activation of myofibroblast into the newt eye, contributing to the scar formation. Macrophages returning to the eye following clodronate treatment are unable to resolve the advanced inflammation and fibrotic environment. Absence of lens, severe fibrosis and chronic inflammation results in microphthalmia

Previous studies have shown that the FGF signaling pathway plays an important role during lens regeneration [96–100], and macrophages can directly secrete FGF in certain conditions [101, 102]. Additionally, FGF2 and macrophages have been previously linked in cancer research. FGF2 has been found to alter macrophage polarization, impacting tumor immunity and growth. Tumor-associated macrophages express high levels of FGF2, influencing their behavior and interactions within the tumor microenvironment [120]. In nasopharyngeal carcinoma, FGF2 signaling modulates pericyte-macrophage crosstalk and metastasis. FGF2 indirectly activates macrophages via pericytes, affecting their migration and polarization towards an M2 phenotype [121]. Macrophages have been identified as a crucial link between angiogenesis and lymphangiogenesis, with different macrophage phenotypes expressing genes involved in promoting angiogenesis, including VEGF-A and FGF2 [122]. Here we show that exogenous administration of FGF2 in the absence of macrophages recovers iPEC proliferation decay, reduces the cellular accumulation characteristic of the fibrotic scar, and has the potential to rescue regeneration. Our results indicate that expression of SOCS3 is downregulated in newt eyes that are supplemented with FGF2, and SOCS3 has been shown to function as a negative regulator of the FGF2 signaling pathway [123]. Furthermore, inhibition of SOCS3 has been shown to promote liver and axon regeneration in mammals [124, 125]. How macrophages either directly or indirectly affect the expression of FGFs in the newt eye requires further exploration. One possible explanation is that, like tumor associated macrophages [120], newt macrophages may directly secrete FGF into the iris to promote proliferation since direct secretion of FGFs have been reported before in other contexts [102, 126]. Alternatively, macrophage depletion could indirectly affect FGF levels by preventing its trafficking from nearby tissue sources (Fig. 8). During lens regeneration, the neuroretina secretes growth factors that are necessary for the reprogramming of iPECs [127, 128]. Also, in line with this hypothesis, neuroretina-derived FGFs are essential for lens development [129, 130]. Here, we show that lentectomized eyes treated with clodronate liposomes exhibit higher levels of collagen in the aqueous and vitreous cavities. Therefore, it is possible that excessive amounts of ECM accumulation in the eye cavity could negatively affect the trafficking of growth factors from the neuroretina into the iPECs [131].

Several studies have demonstrated that the initiation of inflammation is necessary for successful regeneration [93, 132–136]. However, the magnitude and duration of inflammation are also key for determining the wound healing outcome [137, 138]. The resolution of inflammation occurs much faster in regeneration-competent animals compared to non-regenerative species, such as mammals [139, 140]. In fact, pharmacological attenuation of inflammation promotes tissue repair in regeneration-incompetent animals, demonstrating the importance of resolving inflammation during the early stages of wound healing [13, 141]. Increasing evidence suggests that macrophages are responsible for placing the necessary restraints on the inflammatory reaction during the early stages of regeneration [16, 17, 93, 142]. Consistent with these studies, we show that macrophage depletion in the newt eye prolongs the expression of pro-inflammatory cytokine IL1-β.

In mammals, prolonged expression of inflammatory genes often leads to fibrosis and ultimately scarring [143–145]. This phenomenon is exacerbated during aging and repeated damage [146, 147]. Salamanders, on the other hand, have mastered scar-free healing and are thought to be resistant to fibrosis. This ability was highlighted in a pioneering study, in which the lens was removed 18 times from the same newts in a period of 19 years, and each time the lens was perfectly regenerated without any signs of fibrosis or scarring [148, 149]. It is tempting to speculate that the resistance to fibrosis in these animals could be due to newt macrophages adopting an earlier and more robust anti-inflammatory phenotype relative to their mammalian counterparts. However, currently, the newt macrophage polarization states after injury are undefined, and this is a topic of ongoing research by our group and others. Interestingly, in a recent study, Simkin et al. showed that macrophages of *Acomys *spp. adopt a pro-regenerative phenotype and secrete several factors that have the potential to modulate ear pinna regeneration, as opposed to macrophages from the regeneration incompetent mice, *Mus musculus *[108]. Furthermore, in another recent study, Debuque et al. directly compared salamander and mammalian macrophages after exposure to different damage-associated molecular patterns (DAMPs) and pathogen associated molecular patterns (PAMPs) and showed that salamander macrophages evolved distinct signaling mechanisms that could favor regeneration outcome [11].

Macrophage depletion with clodronate is transient, yet the return of macrophages was unable to resolve the ECM accumulation that occurred in their absence. Even in clodronate-early-treated eyes monitored for 100 dpl, we found that the scar-like tissue components (cellular inflammation, ECM accumulation, and fibrosis) progressively got worse, often leading to microphthalmia. However, we show that a secondary injury to the dorsal iris is sufficient to resolve the fibrotic lesion and re-start the process of regeneration. These findings are noteworthy and suggest that while the return of late macrophages is not sufficient to resolve a fibrotic scar in the newt eye, a new injury can recruit (or re-program) the type of macrophage needed to eliminate a previously established fibrotic lesion. Whether some other cell type’s response to injury is required to facilitate the reprogramming of newt macrophages into a pro-resolving and anti-fibrotic phenotype requires further study, but it undoubtedly has exciting potential. Consistent with our observations, Godwin et al., had previously shown that re-amputation of fibrotic limbs after macrophage depletion in axolotls rescued regeneration [17]. In these experiments, the entire fibrotic environment was removed by the amputation. In contrast, for our study, we kept the vitreous and aqueous chambers intact (areas filled with collagens, cellular accumulation, and myofibroblasts) when the dorsal iris epithelium was reinjured. Thus, our findings illustrate that newts not only can re-start a previously failed regeneration process, but they are able to repair previously established fibrotic tissue under the right circumstances. Future studies comparing the phenotypes of newt macrophages during lens regeneration in control animals to those recruited to the fibrotic environment after clodronate treatment and to those after secondary injury have potential therapeutic interest, as this approach could give us clues as to which polarizing factors are responsible for inducing anti-fibrotic and pro-regenerative outcomes in newts.

While this study offers valuable insights, it's important to acknowledge some of its limitations. First, bulk transcriptomic methods were used to characterize the molecular mechanisms that promote wound healing and inflammation resolution during the early stages of regeneration. The iris epithelium and stroma consist of a heterogeneous cell population (iPECs, keratinocytes, melanocytes, muscle, blood, and infiltrating immune cells, such as macrophages). In axolotls and zebrafish, not only immune cells, but also other cell types, such as blastema progenitors, senescent cells and fin epithelial cells (in limb and fin, respectively) can produce cytokines that modulate the inflammatory response and influence the outcome of regeneration [93, 150, 151]. Future studies utilizing technologies with the ability to resolve cell types, such as cell sorting, single-cell RNA sequencing, and/or spatial transcriptomics will shed light on how each cell population contributes differently during lens regeneration. Furthermore, these technologies will allow us to identify the heterogeneity of the cellular accumulation that we observed following clodronate treatment. Another limitation comes from the use of liposomes to deplete macrophages. Since liposomes cannot penetrate the blood–brain barrier, they must be injected intraocularly, thus creating an additional injury to the eye. Since other phagocytic cells have been reported to ingest clodronate-liposomes [92, 152], we are also embarking on pharmacological studies using small molecules to target macrophages. The generation of animals with genetically depleted macrophages will further aid the exploration of macrophage function during scar-free healing in newts.

## Conclusions

Our results demonstrate that newt lens regeneration can be added to the ever-growing list of instances showcasing the necessity of macrophages for successful regeneration in multiple species. This study offers a unique perspective and a first glimpse into the functions of macrophages to achieve successful regeneration of the lens. Specifically, newt macrophages promote early cell cycle re-entry in iPECs, are required for the resolution of pro-inflammatory signals, and prevent fibrotic scar formation during lens regeneration. Furthermore, we show that macrophage depletion during lens regeneration leads to a failure of the regeneration program while also establishing a progressive fibrotic disease state that causes microphthalmia. The fibrotic tissue persists, even after the return of macrophages subsequent to the cessation of clodronate treatment. Remarkably, a secondary injury in the dorsal iris, months after the fibrotic lesion is established, elicits fibrotic scar resolution within the eye and restarts the process of lens regeneration. The reversal of fibrosis with re-injury occurs in the presence of returning macrophages, highlighting their potential role in clearing the previously established fibrotic disease. Taking into consideration that fibrosis in humans is often considered irreversible, these significant observations bear great translational potential. Our findings establish a new experimental model and context in which the mechanisms behind scar-free healing, regeneration, and scar reabsorption can be studied further.

### Supplementary Information

**Additional file 1**: Statistical analysis.

**Additional file 2**: **Fig S1.** mpeg1:GFP transgenic newts enable the in vivo labeling of macrophages. **A**, **D**, **G**, **J**, **M** Representative fluorescence images of sections of 5-month-old mpeg1:GFP newts showing presence of eGFP+ cells in the tail, trunk and head sections. **B** Anti-GFP immunofluorescence staining. **C** Merge of mpeg1:GFP endogenous fluorescence, anti-GFP and Hoechst. **E**, **H** F4/80 immunofluorescence staining. **F**, **I** Merge of mpeg1:GFP, F4/80 and Hoechst. **K**, **N** L-plastin immunofluorescence staining. **L**, **O** Merge of mpeg1:GFP, L-plastin and Hoechst. Arrows represent colocalization events. **P** Percentage of colocalization of endogenous eGFP (average from tail, trunk and head sections) with anti-GFP (97.5%), F4/80 (41.4%) and L-plastin (21.6%). Scale bar: 50 µm; n=3. Related to Fig. 1. **Figure S2.** Time-dependent regulation of KEGG pathways in the injured dorsal iris of *Notophthalmus viridescens.* A time course expression analysis was performed of the dorsal iris through 4 dpl. The shown pathways were overrepresented amongst transcripts that exhibited time-dependent regulatory patterns. The displayed expression values in each box represent the expression of homologous transcripts, ordered from left to right by time beginning with the intact iris. Color scale represents Z-score of expression values. Related to Fig. 2B. **Figure S3.** Clodronate treatment does not affect the survival of iPECs during the early stages of lens regeneration. (A) TUNEL assay was used to visualize apoptotic nuclei from control- and clodronate-treated animals at 1, 4, 10, 15, and 30 dpl (paraffin embedded tissue). Dashed lines were used to mark the iris epithelium. Inset images of the dorsal iPECs highlight the effects of macrophage depletion on cell survival. As expected, TUNEL+ nuclei were observed in the vitreous and aqueous chambers of clodronate-liposome treated eyes (arrows) but not in PBS-liposome treated eyes. At 15 (Stage IV-V) and 30 dpl (Stage VIII) TUNEL+ nuclei were found in the lens epithelial layer of PBS-liposome treated eyes (arrowhead); n=6 per time point. Scale bars: 200µm (overviews, left) and 50µm (insets, right). **Figure S4.** Late clodronate-liposome administration impairs lens growth by increasing apoptosis instead of affecting proliferation. **A** Schematic representation of experimental design. Clodronate or PBS liposomes were injected intraocularly in the aqueous chamber at 20, 22, and 24 dpl in the presence of the regenerating lens. **B** Clodronate liposome administration at 20 dpl did not inhibit the proliferation and mitosis levels of lens epithelial cells; n=6. Scale bars: 100µm (paraffin embedded tissue). **C** Picrosirius red staining revealed a stronger collagen staining (red) in the vitreous and aqueous chamber of the clodronate liposome treated eyes; n=6. Scale bars: 100µm (paraffin embedded tissue). **D** Apoptotic cells were detected inside the lens fibers and at the surrounding area of the ventral iris following macrophage depletion at 20 dpl; n=6. Scale bars: 100µm. Related to Fig. 6 (paraffin embedded tissue). **Table S1:** Oligos and target sequences used for RT-qPCR.

**Additional file 3**: **Video S1.** Time-lapse imaging of eGFP fluorescence in mpeg1:GFP transgenic newts. Movie of the dorsal view of an F2:mpeg1:GFP+ embryo. Positive macrophages and microglia of different morphologies are widespread. Border-associated macrophages can be seen floating in the cerebrospinal fluid inside the 4th ventricle. Related to Fig. 1A, B.

**Additional file 4**: **Video S2.** Phagocytic activity of eGFP + cells in the brain. In vivo observation of GeRPs right after intraventricular injection in an F1:mpeg1:GFP larva. Arrowhead point to the first events of GeRPs internalization by mpeg1:GFP+ cells. Time-lapse images used to produce this movie were acquired during a total time of 5 minutes. Related to Fig. 1D, E.

**Additional file 5**: **Video S3.** Phagocytic activity of eGFP + cells in the spinal cord. In vivo observation of GeRPs after intraventricular injection in an F1:mpeg1:GFP larva. Arrowhead point to an mpeg1:GFP+ cell approaching a single GeRP to potentially initiate phagocytosis. Time-lapse images used to produce this movie were acquired during a total time of 10 minutes. Related to Fig. 1D, E.

**Additional file 6**: **Video S4.** Three-dimensional representation of OCT images from PBS treated eyes at 4 dpl. Animated rendering of three-dimensional images, reconstructed from OCT C-scans. Eye tissues (cornea/green, iris/blue, ECM/yellow, regenerating lens/red) were manually pseudo colored to aid in visualization. ECM accumulation is observed at 4 dpl (Stage 0-I).

**Additional file 7**: **Video S5.** Three-dimensional representation of OCT images from PBS treated eyes at 10 dpl. Animated rendering of three-dimensional images, reconstructed from OCT C-scans. Eye tissues (cornea/green, iris/blue, ECM/yellow, regenerating lens/red) were manually pseudo colored to aid in visualization. By the 10 dpl (Stage I-II) ECM is mostly cleared out and the vitreous/aqueous chambers appear clear.

**Additional file 8**: **Video S6.** Three-dimensional representation of OCT images from PBS treated eyes at 21 dpl. Animated rendering of three-dimensional images, reconstructed from OCT C-scans. Eye tissues (cornea/green, iris/blue, ECM/yellow, regenerating lens/red) were manually pseudo colored to aid in visualization. Following ECM clearing the formation of the new lens vesicle is visible at 21 dpl (Stage VI-VII).

**Additional file 9**: **Video S7.** Three-dimensional representation of OCT images from clodronate treated eyes at 4 dpl. Animated rendering of three-dimensional images, reconstructed from OCT C-scans. Eye tissues (cornea/green, iris/blue, ECM/yellow, regenerating lens/red) were manually pseudo colored to aid in visualization. ECM accumulation is observed at 4 dpl (Stage 0-I).

**Additional file 10**: **Video S8.** Three-dimensional representation of OCT images from clodronate treated eyes at 10 dpl. Animated rendering of three-dimensional images, reconstructed from OCT C-scans. Eye tissues (cornea/green, iris/blue, ECM/yellow, regenerating lens/red) were manually pseudo colored to aid in visualization. Unlike the control eyes, in clodronate treated eyes ECM fails to clear out by 10dpl.

**Additional file 11**: **Video S9.** Three-dimensional representation of OCT images from clodronate treated eyes at 21 dpl. Animated rendering of three-dimensional images, reconstructed from OCT C-scans. Eye tissues (cornea/green, iris/blue, ECM/yellow, regenerating lens/red) were manually pseudo colored to aid in visualization. ECM and cellular accumulation increases in clodronate treated eyes by 21 dpl.

## Data Availability

The RNA-seq data discussed in this publication have been deposited in NCBI’s Gene Expression Omnibus and are accessible through GEO Series accession number GSE236908, which can be found at https://www.ncbi.nlm.nih.gov/geo/query/acc.cgi?acc=GSE236908. All code, resultant output files, and any additional information required to reanalyze the data reported in this paper are available upon request to the lead contact. Both the plasmid Tol2-mpeg1:eGFP-polyA and the transgenic newt line *P. waltl* tgTol2(Dre.*mpeg1:eGFP*)^MHY/SIMON^ are available to the scientific community upon request. Further information and requests for resources and reagents should be directed to and will be fulfilled by the lead contact, Katia Del Rio-Tsonis (delriok@miamioh.edu).

## Abbreviations

iPECs: Iris pigment epithelial cells
hpl: Hours post-lentectomy
dpl: Days post-lentectomy
FGF: Fibroblast growth factor
GeRPs: Glucan-encapsulated siRNA particles
SD-OCT: Spectral domain optical coherence tomography
Mpeg1: Macrophage expressed gene 1
ECM: Extracellular matrix
LECs: Lens epithelial cells
