# Supplementary material for: Macrophages modulate fibrosis during newt lens regeneration
Source: Stem Cell Res Ther. 2024 May 14;15:141. doi: 10.1186/s13287-024-03740-1 (PMC11094960; doi:10.1186/s13287-024-03740-1)

Supplementary material for ‘Macrophages modulate fibrosis during newt lens regeneration’: Statistical Analysis

Tsissios et al.

Contents

EdU Experiments (Figures 3E and 5B) **1**

Experimental Setup . . . 1

Analysis Method . . . 2

Figure 3E: PBS liposome vs. Clodronate liposome . . . 2

Figure 5B: CLODRONATE + PBS vs. CLODRONATE + FGF . . . 8

RT-qPCR Analyses (Figures 4A, 5D, and 6E) **15**

Figure 4A: Comparing IL1b using Time and Treatment (PBS vs. Clodronate) as Factors . . . 15

Figure 5D: Comparing Nine Gene Expressions using Time (4 vs. 10 dpl) and Treatment (Heparin vs. FGF2) as Factors . . . 27

Figure 6E: Late Macrophage Depletion . . . 45

Eye Diameters (Figure 7B) **53**

EdU Experiments (Figures 3E and 5B)

Experimental Setup

This experiment used individual eyes as the experimental units. Four eyes were used per treatment, and we assume these four eyes are independent experimental units, even though some may come from the same animal. Based on how the experiment was conducted, we are unable to reanalyze the data accounting for possible dependence among pairs of eyes. However, for cell cycle re-entry in lens regeneration it doesn’t appear to be the case that a systemic response is triggered. That is, Yamada and Roesel (1969) showed the iPECs of the contralateral uninjured eye do not re-enter the cell cycle. Though they found that some cells such as macrophages inside the stroma did re-enter the cell cycle, in our analysis below we didn’t include any cells from the iris stroma or other areas of the eye, outside the iris epithelium.

Originally, the experimental plan was to have two factors: the main Treatment factor had two levels (Control-macrophage vs. Treatment-no macrophage); and the Time factor with three levels (4, 10, and 15 dpl). Note that each level of Time received independent units. So the total number of eyes used was 24. However, after the experiment was done, we realized that the 10 and 15 dpl conditions were unnecessary for several reasons. We observed that under the Treatment condition there was no lens regeneration even at 100 dpl. Also, the Clodronate-treated eyes were too damaged after 10 days to clearly differentiate iris pigmented epithelial cells

from those cells we wished to quantify. Though we didn't quantify for 10 and 15 dpl, we observed very few EdU-positive cells in the clodronate treatments.

Thus, we are left with a simpler set of data: Control vs. Treatment at Day 4. We include the original intention of the experiment for the sake of transparency, though it shouldn't affect the results of the smaller dataset we report.

For each eye, there were 30-35 cross-sections across 10 slides. The underlying response of interest was the number of cells of a certain type in the eye. To make the cell counting tractable, cells on slide 3, 5, and 7 were counted, with three cross-sections per slide. Within each cross-section, they counted EdU and HOECHST cells on both the dorsal and ventral areas. We will focus on the dorsal area in this work and are ultimately interested in the proportion of HOECHST cells that are EdU.

Yamada, T., & Roesel, M. E. (1969). Activation of DNA replication in the iris epithelium by lens removal. Journal of Experimental Zoology, 171(4), 425-431.

Analysis Method

Take the EdU cells divided by the HOECHST cells on the dorsal area, aggregated over all nine cross-sections. More specifically, let C_{Ei} be the number of EdU cells on cross-section i, while C_{Di} is the number of HOECHST cells on cross-section i. This response variable would be computed as follows:

$$P_E = \frac{C_E}{C_D} = \frac{\sum_{i=1}^{9} C_{Ei}}{\sum_{i=1}^{9} C_{Di}}$$

Since this is a rate, it typically makes sense to take the natural log of the response, so that the actual response variable that we consider is $\log(P_E)$. This response variable would lead to a simple two-sample t-test, where we have four replicates for the Treatment condition, and four replicates for the Control condition.

However, since the response of interest, EdU cells, is a count, using Poisson regression is more natural because the Poisson distribution models counts directly. We can account for the varying number of HOECHST cells by including $\log(HOECHST)$ as an offset in the Poisson regression model. In this case, the Treatment factor is a categorical predictor.

Finally, a restrictive aspect of the Poisson distribution is that its mean and variance are required to be the same. In many datasets, the variance is larger than the mean, which is called overdispersion. An approach to handle this is Negative Binomial regression. Based on the data, we ended up recommending and using *the Negative Binomial analysis.*

Figure 3E: PBS liposome vs. Clodronate liposome

Data Handling

```
dataTrt1 <- read.xlsx(xlsxFile = "EdU_Expt1_Cleaned.xlsx", sheet="PBS LIPOSOME", fillMergedCells = TRUE
dataTrt2 <- read.xlsx(xlsxFile = "EdU_Expt1_Cleaned.xlsx", sheet="CLODRONATE LIPOSOME", fillMergedCells

dataTrt1$Trt <- "PBS liposome"
dataTrt2$Trt <- "Clodronate liposome"

# Combine the two datasets together
data <- bind_rows(dataTrt1,dataTrt2)
head(data) # display the first few rows of the data
```

```
##       Rep   Slide   Section      Area HOECHST EdU          Trt
## 1 EYE #1  Slide 3 Section 1   dorsal      35  10 PBS liposome
## 2 EYE #1  Slide 3 Section 1 ventral       34   0 PBS liposome
## 3 EYE #1  Slide 3 Section 1    total      69  10 PBS liposome
## 4 EYE #1  Slide 3 Section 2   dorsal      36  13 PBS liposome
## 5 EYE #1  Slide 3 Section 2 ventral       34   5 PBS liposome
## 6 EYE #1  Slide 3 Section 2    total      70  18 PBS liposome
```

Next is some data wrangling that cleans things up and sums up cell counts over cross-sections. This prepares the data for the two-sample t-test, which we provide though the analysis is not recommended (in the paper we use a Negative Binomial analysis; see below).

```
# note that `EYE #1 ` has an extra space at the end.
data2 <- data %>%
  mutate(Eye=recode_factor(Rep,`EYE #1 `="1",`EYE #2`="2",`EYE #3`="3",`EYE #4`="4"),
         Slide=recode_factor(Slide,`Slide 3`="3",`Slide 5`="5",`Slide 7`="7"),
         Section=recode_factor(Section,`Section 1`="1",`Section 2`="2",`Section 3`="3",
                              `Section 4`="4",`Section 5`="5",`Section 6`="6",
                              `Section 7`="7",`Section 8`="8",`Section 9`="9"),
         Area=recode_factor(Area,`dorsal`="Dorsal",`ventral `="Ventral",`total`="Total")) %>%
  mutate(Trt=factor(Trt)) %>%
  dplyr::select(-Rep) %>%
  filter(Area=="Dorsal") %>% # only Dorsal area
  mutate(EdU_HOECHST_ratio=EdU/HOECHST)

data2sampF <- data2 %>%
  group_by(Trt,Eye) %>%
  # Total cells, added over EDU and HOECHST
  summarize(HOECHST_T=sum(HOECHST),EdU_T=sum(EdU)) %>%
  mutate(EdU_HOECHST_ratio=EdU_T/HOECHST_T)
```

```
## `summarise()` has grouped output by 'Trt'. You can override using the `.groups`
## argument.
```

```
  data2sampF
```

```
## # A tibble: 8 x 5
## # Groups:   Trt [2]
##   Trt                 Eye   HOECHST_T EdU_T EdU_HOECHST_ratio
##   <fct>               <fct>     <dbl> <dbl>             <dbl>
## 1 Clodronate liposome 1           304    19            0.0625
## 2 Clodronate liposome 2           384    25            0.0651
## 3 Clodronate liposome 3           307    20            0.0651
## 4 Clodronate liposome 4           306    31            0.101
## 5 PBS liposome        1           226    64            0.283
## 6 PBS liposome        2           271    42            0.155
## 7 PBS liposome        3           295    39            0.132
## 8 PBS liposome        4           236    44            0.186
```

Plots and Two-sample t-test

Now plot the data, then a two-sample t-test. We will plot both in the original and log scale.

```
ggplot(data2sampF, aes(x=Trt,y=EdU_HOECHST_ratio)) +
  geom_jitter(width=.05,height=0)+
  theme_bw()
```

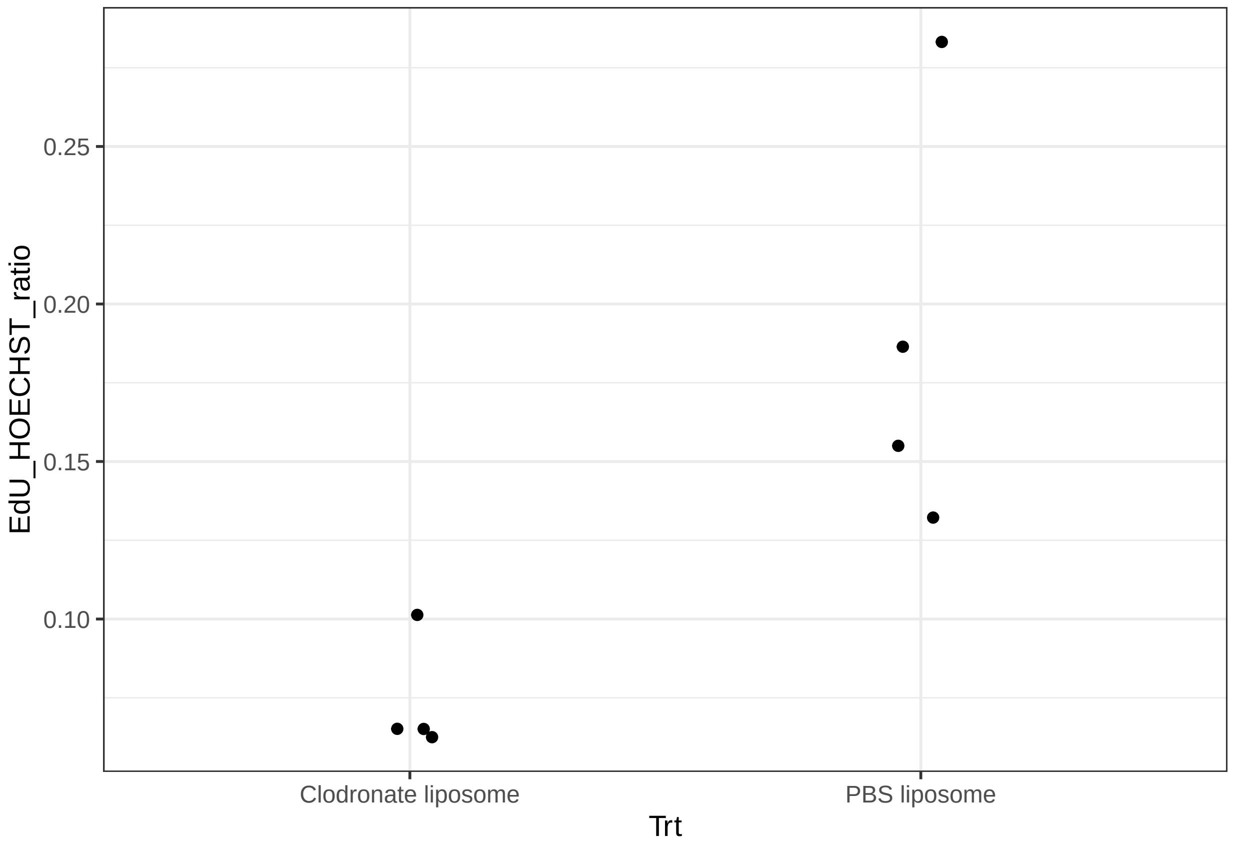


```
ggplot(data2sampF, aes(x=Trt,y=log(EdU_HOECHST_ratio))) +
  geom_jitter(width=.05,height=0)+
  theme_bw()
```

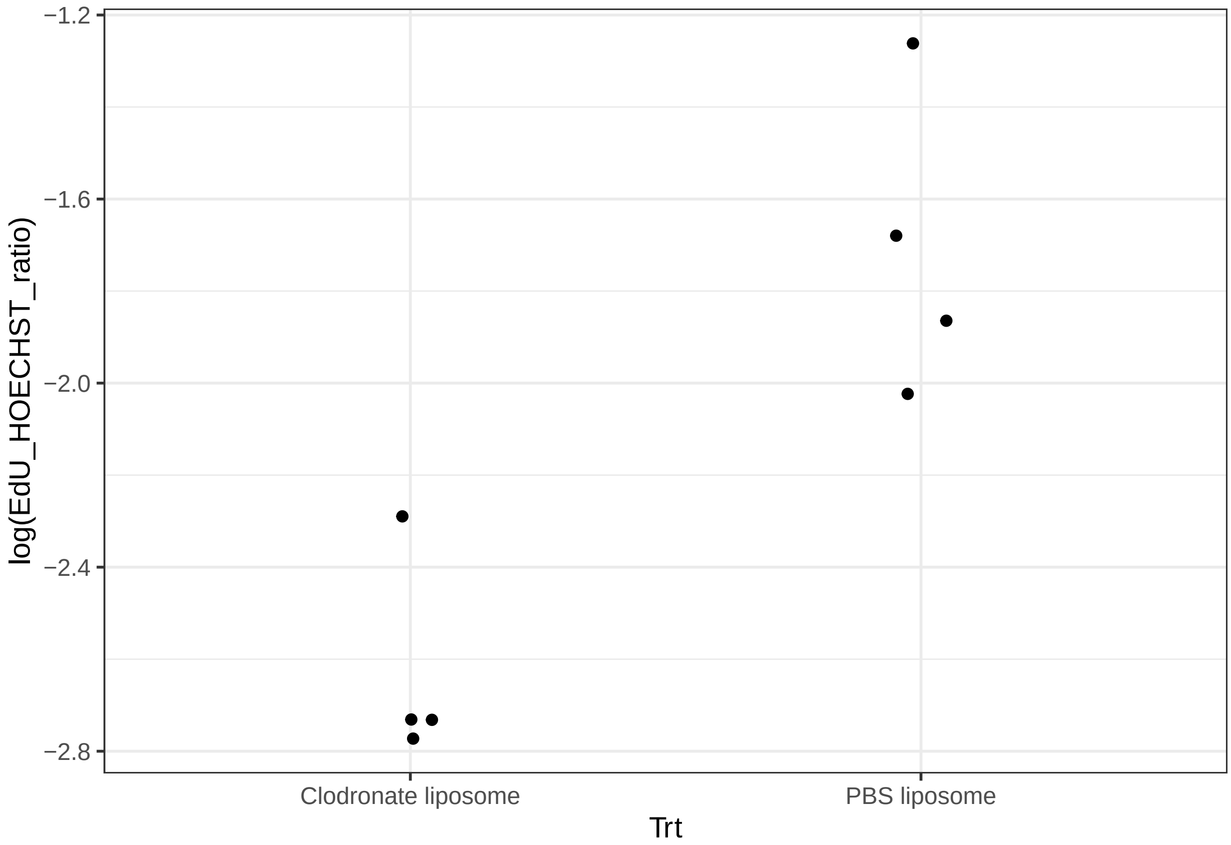


```
t.test(log(EdU_HOECHST_ratio)~Trt,data=data2sampF,var.equal=FALSE)
```

```
##
##  Welch Two Sample t-test
##
## data:  log(EdU_HOECHST_ratio) by Trt
## t = -4.6162, df = 5.3525, p-value = 0.004852
## alternative hypothesis: true difference in means between group Clodronate liposome and group PBS lipo
## 95 percent confidence interval:
##  -1.4284790 -0.4194769
## sample estimates:
## mean in group Clodronate liposome        mean in group PBS liposome
##                         -2.631267                         -1.707289
```

Because of the nature of the data, it is better to analyze the log of the ratio. The interpretation is less straightforward, but you can say that there is strong evidence that the mean of log ratio of the two conditions is not the same. As we can see, the PBS liposome has the larger mean.

However, instead of the t-test, we prefer an analysis which handles the cell counts more naturally, one which uses a distribution with integer support.

Analyzing the Data Directly Using Counts

Now we will fit a series of generalized linear models. First, Poisson to model the number of EdU Cells directly. We use the natural log of HOECHST cells as an offset, which essentially accounts for the fact that the larger the number of total cells the larger we'd expect the number of EdU cells to be.

```r
summary(glm(EdU_T~Trt,family="poisson",offset=log(HOECHST_T),data=data2sampF))
```

```
##
## Call:
## glm(formula = EdU_T ~ Trt, family = "poisson", data = data2sampF,
##     offset = log(HOECHST_T))
##
## Deviance Residuals:
##     Min       1Q   Median       3Q      Max
## -2.1792  -0.8070  -0.5526   0.5016   3.2239
##
## Coefficients:
##                  Estimate Std. Error z value Pr(>|z|)
## (Intercept)       -2.6170     0.1026 -25.507  < 2e-16 ***
## TrtPBS liposome    0.9234     0.1258   7.342  2.1e-13 ***
## ---
## Signif. codes:  0 '***' 0.001 '**' 0.01 '*' 0.05 '.' 0.1 ' ' 1
##
## (Dispersion parameter for poisson family taken to be 1)
##
##     Null deviance: 78.311  on 7  degrees of freedom
## Residual deviance: 20.536  on 6  degrees of freedom
## AIC: 67.22
##
## Number of Fisher Scoring iterations: 4
```

Since Residual deviance is more than 3 times its degrees of freedom, overdispersion is of concern. That is, the Poisson procedure assumes the mean of the count is the same as the variance of the count, and it appears that this data does not conform to this assumption. To account for this we can fit a Negative Binomial regression which allows for overdispersion.

```r
glm.fit <- glm.nb(EdU_T~Trt + offset(log(HOECHST_T)),data=data2sampF)
summary(glm.fit)
```

```
##
## Call:
## glm.nb(formula = EdU_T ~ Trt + offset(log(HOECHST_T)), data = data2sampF,
##     init.theta = 23.86734097, link = log)
##
## Deviance Residuals:
##     Min       1Q   Median       3Q      Max
## -1.3212  -0.5727  -0.4009   0.2834   1.7715
##
## Coefficients:
##                  Estimate Std. Error z value Pr(>|z|)
## (Intercept)       -2.6135     0.1450  -18.03  < 2e-16 ***
## TrtPBS liposome    0.9390     0.1916    4.90 9.58e-07 ***
## ---
## Signif. codes:  0 '***' 0.001 '**' 0.01 '*' 0.05 '.' 0.1 ' ' 1
##
## (Dispersion parameter for Negative Binomial(23.8673) family taken to be 1)
##
```

```
##     Null deviance: 31.5753  on 7  degrees of freedom
## Residual deviance:  7.4428  on 6  degrees of freedom
## AIC: 63.234
##
## Number of Fisher Scoring iterations: 1
##
##
##               Theta:  23.9
##           Std. Err.:  18.9
##
##  2 x log-likelihood:  -57.234
```

```
newdata <- data.frame(Trt=c("Clodronate liposome", "PBS liposome"), HOECHST_T=c(1,1))
pdctd <- predict(glm.fit,newdata,type="response",se.fit=TRUE)
```

The above Negative Binomial regression is our preferred analysis. There is strong evidence of a difference between the treatments, in terms of log of expected EdU cell counts, when accounting for the number of HOECHST cells.

Figure 3E:

```
data2sampF$Trt <- factor(data2sampF$Trt,levels=c("PBS liposome","Clodronate liposome"))

ggplot(data2sampF, aes(x=Trt,y=EdU_HOECHST_ratio)) +
  geom_jitter(width=.05,height=0, size=3)+
  geom_point(x="Clodronate liposome", y=pdctd$fit[1], color="blue", shape=17,size=5)+
  geom_point(x="PBS liposome", y=pdctd$fit[2], color="blue",shape=17,size=5)+  theme_bw() +
  geom_errorbar(aes(x="Clodronate liposome", ymin=pdctd$fit[1]-pdctd$se.fit[1], ymax=pdctd$fit[1]+pdctd$
  geom_errorbar(aes(x="PBS liposome", ymin=pdctd$fit[2]-pdctd$se.fit[2], ymax=pdctd$fit[2]+pdctd$se.fit
  labs(x="Treatment",y="EdU:HOECHST Ratio") +
    theme(aspect.ratio=2/2.2, text = element_text(size = 16), axis.text= element_text(size=12)) +
  annotate("text",x=1.5,y=0.25, label="p < 0.001", size=6)
```

```
## Warning: Using `size` aesthetic for lines was deprecated in ggplot2 3.4.0.
## i Please use `linewidth` instead.
## This warning is displayed once every 8 hours.
## Call `lifecycle::last_lifecycle_warnings()` to see where this warning was
## generated.
```

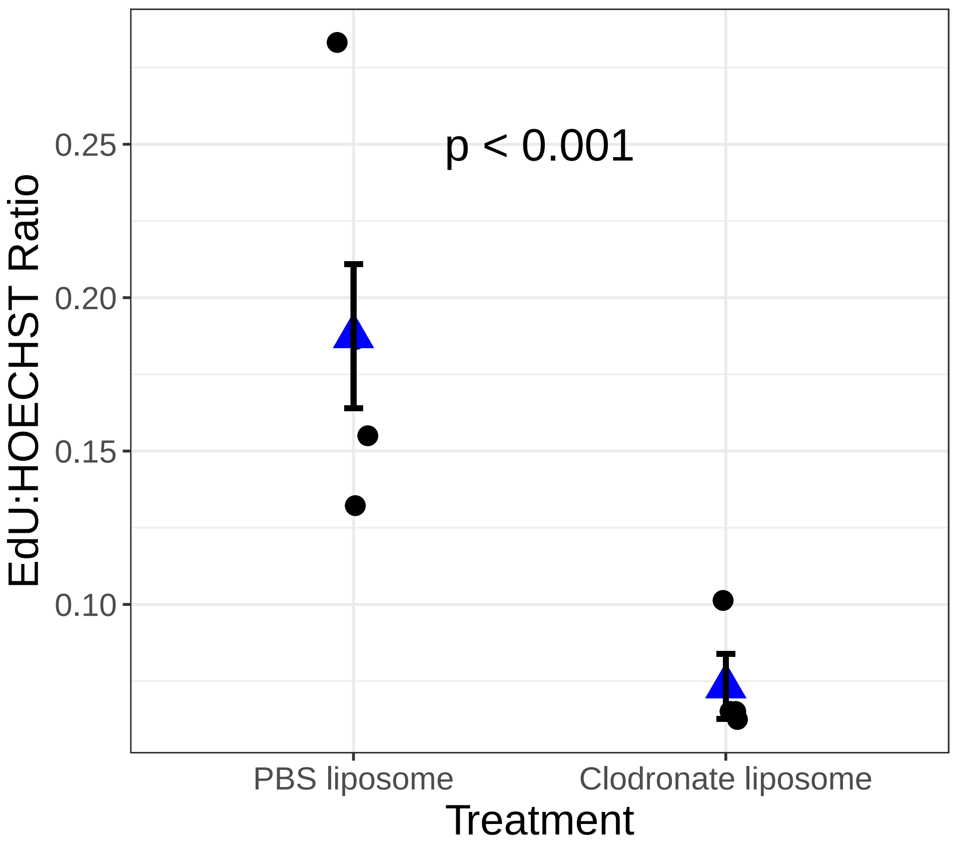

Note that the points in the plot above may vary slightly from those provided in the paper. This is because we have jittered the data points slightly in the plot (not in the analysis) in order to better observe them.

Figure 5B: CLODRONATE + PBS vs. CLODRONATE + FGF

Data Handling

Similar to EdU Experiment 1.

```
dataTrt1 <- read.xlsx(xlsxFile = "EdU_Expt2_Cleaned.xlsx", sheet="CLODRONATE AND PBS", fillMergedCells
dataTrt2 <- read.xlsx(xlsxFile = "EdU_Expt2_Cleaned.xlsx", sheet="CLODRONATE AND FGF", fillMergedCells
dataTrt1$Trt <- "Clodronate and PBS"
dataTrt2$Trt <- "Clodronate and FGF2"
# Combine the two datasets together
data <- bind_rows(dataTrt1,dataTrt2)
head(data) # display first few rows of the data
```

```
##       Rep   Slide   Section    Area HOECHST EdU                Trt
## 1 EYE #1  Slide 3 Section 1  dorsal      41   2 Clodronate and PBS
## 2 EYE #1  Slide 3 Section 1 ventral      29   1 Clodronate and PBS
## 3 EYE #1  Slide 3 Section 1   total      70   3 Clodronate and PBS
## 4 EYE #1  Slide 3 Section 2  dorsal      45   1 Clodronate and PBS
## 5 EYE #1  Slide 3 Section 2 ventral      34   1 Clodronate and PBS
## 6 EYE #1  Slide 3 Section 2   total      79   2 Clodronate and PBS
```

Data wrangling, showing the data after we've aggregated over the cross-sections.

```r
# note that `EYE #1 ` has an extra space at the end.
data2 <- data %>%
  mutate(Eye=recode_factor(Rep,`EYE #1 `="1",`EYE #2`="2",`EYE #3`="3",`EYE #4`="4"),
         Slide=recode_factor(Slide,`Slide 3`="3",`Slide 5`="5",`Slide 7`="7"),
         Section=recode_factor(Section,`Section 1`="1",`Section 2`="2",`Section 3`="3",
                               `Section 4`="4",`Section 5`="5",`Section 6`="6",
                               `Section 7`="7",`Section 8`="8",`Section 9`="9"),
         Area=recode_factor(Area,`dorsal`="Dorsal",`ventral `="Ventral",`total`="Total")) %>%
  mutate(Trt=factor(Trt)) %>%
  dplyr::select(-Rep) %>%
  filter(Area=="Dorsal") %>% # only Dorsal area
  mutate(EdU_HOECHST_ratio=EdU/HOECHST)

data2sampF <- data2 %>%
  group_by(Trt,Eye) %>%
  # Total cells, added over EDU and HOECHST
  summarize(HOECHST_T=sum(HOECHST),EdU_T=sum(EdU)) %>%
  mutate(EdU_HOECHST_ratio=EdU_T/HOECHST_T)
```

```
## `summarise()` has grouped output by 'Trt'. You can override using the `.groups`
## argument.
```

```r
  data2sampF
```

```
## # A tibble: 8 x 5
## # Groups:   Trt [2]
##   Trt                 Eye   HOECHST_T EdU_T EdU_HOECHST_ratio
##   <fct>               <fct>     <dbl> <dbl>             <dbl>
## 1 Clodronate and FGF2 1           274    37           0.135
## 2 Clodronate and FGF2 2           139    11           0.0791
## 3 Clodronate and FGF2 3           212    59           0.278
## 4 Clodronate and FGF2 4           197    29           0.147
## 5 Clodronate and PBS  1           233     5           0.0215
## 6 Clodronate and PBS  2           194     5           0.0258
## 7 Clodronate and PBS  3           205     2           0.00976
## 8 Clodronate and PBS  4           181    10           0.0552
```

Plots and Two-sample t-test

As before, we will show the two-sample t-test analysis, but ultimately use a Negative Binomial approach.

```r
ggplot(data2sampF, aes(x=Trt,y=EdU_HOECHST_ratio)) +
  geom_jitter(width=.05,height=0)+
  theme_bw()
```

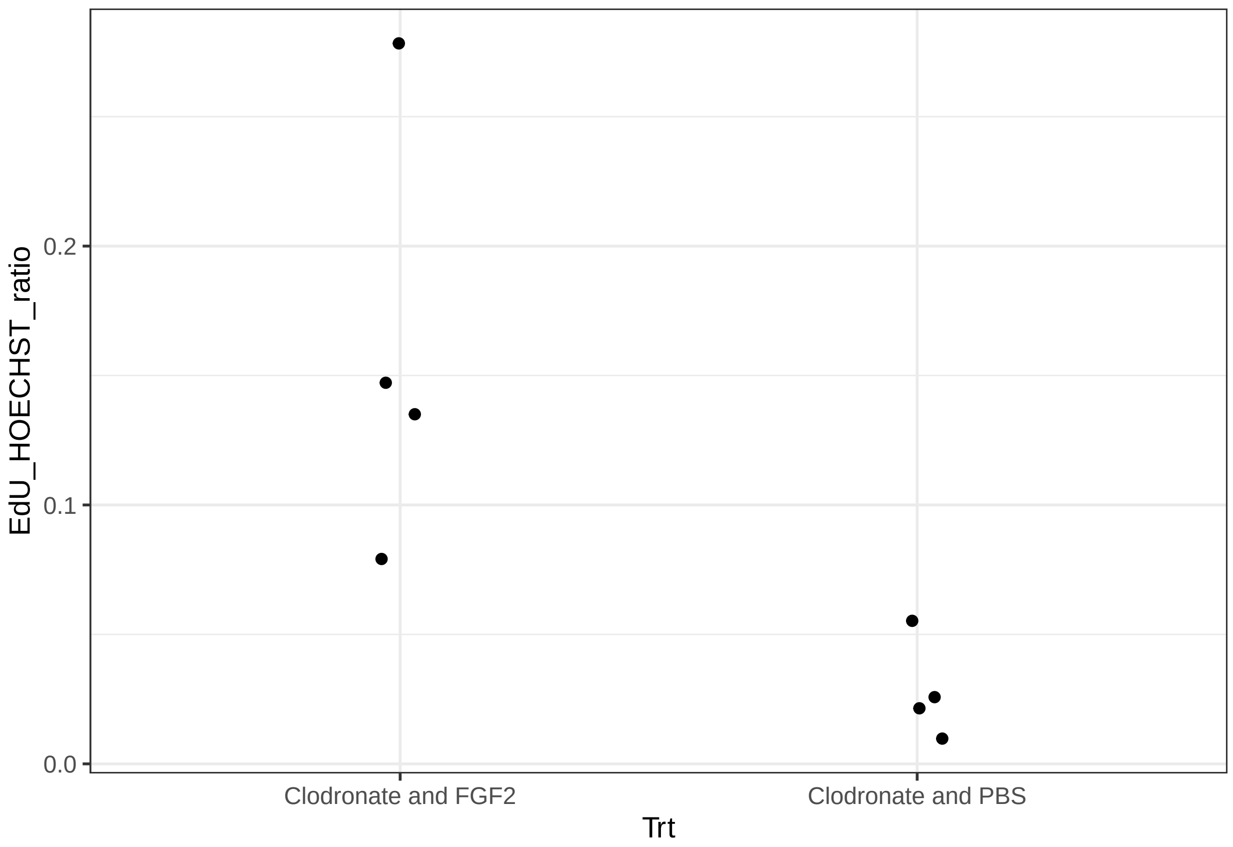


```r
ggplot(data2sampF, aes(x=Trt,y=log(EdU_HOECHST_ratio))) +
  geom_jitter(width=.05,height=0)+
  theme_bw()
```

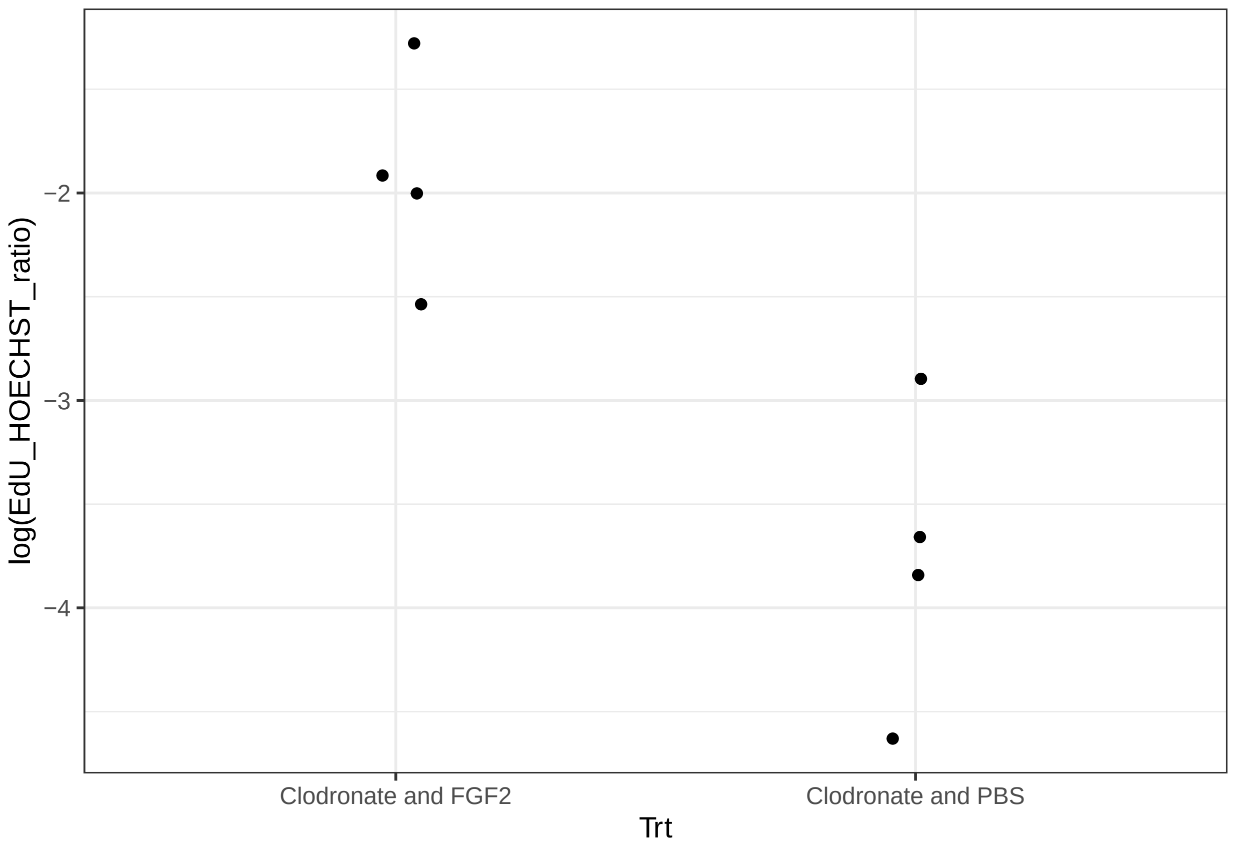


```
t.test(log(EdU_HOECHST_ratio)~Trt,data=data2sampF,var.equal=FALSE)
```

```
##
##  Welch Two Sample t-test
##
## data:  log(EdU_HOECHST_ratio) by Trt
## t = 4.1485, df = 5.4675, p-value = 0.007364
## alternative hypothesis: true difference in means between group Clodronate and FGF2 and group Clodrona
## 95 percent confidence interval:
##  0.7218401 2.9241848
## sample estimates:
## mean in group Clodronate and FGF2  mean in group Clodronate and PBS
##                         -1.933436                         -3.756449
```

Analyzing the Data Directly Using Counts

Again, we will fit a Poisson model to handle the EdU counts more directly, with natural log of HOECHST cells as the offset.

```
summary(glm(EdU_T~Trt,family="poisson",offset=log(HOECHST_T),data=data2sampF))
```

```
##
## Call:
## glm(formula = EdU_T ~ Trt, family = "poisson", data = data2sampF,
```

```
##     offset = log(HOECHST_T))
##
## Deviance Residuals:
##     Min       1Q   Median       3Q      Max
## -2.7875  -1.3932  -0.5905   0.4220   3.6763
##
## Coefficients:
##                       Estimate Std. Error z value Pr(>|z|)
## (Intercept)           -1.79909    0.08575 -20.981  < 2e-16 ***
## TrtClodronate and PBS -1.81060    0.22979  -7.879 3.29e-15 ***
## ---
## Signif. codes:  0 '***' 0.001 '**' 0.01 '*' 0.05 '.' 0.1 ' ' 1
##
## (Dispersion parameter for poisson family taken to be 1)
##
##     Null deviance: 120.973  on 7  degrees of freedom
## Residual deviance:  30.721  on 6  degrees of freedom
## AIC: 69.286
##
## Number of Fisher Scoring iterations: 4
```

Since Residual deviance is more than 5 times its degrees of freedom, overdispersion is a concern, so we fit a Negative Binomial regression which allows for overdispersion.

```
summary(glm.nb(EdU_T~Trt + offset(log(HOECHST_T)),data=data2sampF))
```

```
##
## Call:
## glm.nb(formula = EdU_T ~ Trt + offset(log(HOECHST_T)), data = data2sampF,
##     init.theta = 6.433900451, link = log)
##
## Deviance Residuals:
##     Min       1Q   Median       3Q      Max
## -1.3801  -0.6585  -0.3075   0.2681   1.4134
##
## Coefficients:
##                       Estimate Std. Error z value Pr(>|z|)
## (Intercept)            -1.8245     0.2163  -8.437  < 2e-16 ***
## TrtClodronate and PBS  -1.7687     0.3613  -4.896  9.8e-07 ***
## ---
## Signif. codes:  0 '***' 0.001 '**' 0.01 '*' 0.05 '.' 0.1 ' ' 1
##
## (Dispersion parameter for Negative Binomial(6.4339) family taken to be 1)
##
##     Null deviance: 32.5807  on 7  degrees of freedom
## Residual deviance:  8.1636  on 6  degrees of freedom
## AIC: 58.147
##
## Number of Fisher Scoring iterations: 1
##
##
##               Theta:  6.43
##           Std. Err.:  4.82
```

```
##
##  2 x log-likelihood:  -52.147
```

```
glm.fit <- glm.nb(EdU_T~Trt + offset(log(HOECHST_T)),data=data2sampF)
summary(glm.fit)
```

```
##
## Call:
## glm.nb(formula = EdU_T ~ Trt + offset(log(HOECHST_T)), data = data2sampF,
##     init.theta = 6.433900451, link = log)
##
## Deviance Residuals:
##     Min       1Q   Median       3Q      Max
## -1.3801  -0.6585  -0.3075   0.2681   1.4134
##
## Coefficients:
##                       Estimate Std. Error z value Pr(>|z|)
## (Intercept)            -1.8245     0.2163  -8.437  < 2e-16 ***
## TrtClodronate and PBS  -1.7687     0.3613  -4.896  9.8e-07 ***
## ---
## Signif. codes:  0 '***' 0.001 '**' 0.01 '*' 0.05 '.' 0.1 ' ' 1
##
## (Dispersion parameter for Negative Binomial(6.4339) family taken to be 1)
##
##     Null deviance: 32.5807  on 7  degrees of freedom
## Residual deviance:  8.1636  on 6  degrees of freedom
## AIC: 58.147
##
## Number of Fisher Scoring iterations: 1
##
##
##               Theta:  6.43
##           Std. Err.:  4.82
##
##  2 x log-likelihood:  -52.147
```

```
newdata <- data.frame(Trt=c("Clodronate and PBS", "Clodronate and FGF2"), HOECHST_T=c(1,1))
pdctd2 <- predict(glm.fit,newdata,type="response",se.fit=TRUE)
pdctd2
```

```
## $fit
##          1          2
## 0.02751087 0.16129585
##
## $se.fit
##           1           2
## 0.007961443 0.034882558
##
## $residual.scale
## [1] 1
```

Again, *the above Negative Binomial regression is the preferred analysis.* There is strong evidence of a difference between the treatments, in terms of log of expected EdU cell counts, when accounting for the number of HOECHST cells.

Figure 5B:

```
data2sampF$Trt <- factor(data2sampF$Trt,levels=c("Clodronate and PBS","Clodronate and FGF2"))

Fig5B <- ggplot(data2sampF, aes(x=Trt,y=EdU_HOECHST_ratio)) +
  geom_jitter(width=.05,height=0, size=3)+
  geom_point(x="Clodronate and PBS", y=pdctd2$fit[1], color="blue", shape=17,size=5)+
  geom_point(x="Clodronate and FGF2", y=pdctd2$fit[2], color="blue",shape=17,size=5)+  theme_bw() +
  geom_errorbar(aes(x="Clodronate and PBS", ymin=pdctd2$fit[1]-pdctd2$se.fit[1], ymax=pdctd2$fit[1]+pdct
  geom_errorbar(aes(x="Clodronate and FGF2", ymin=pdctd2$fit[2]-pdctd2$se.fit[2], ymax=pdctd2$fit[2]+pdc
  labs(x="Treatment",y="EdU:HOECHST Ratio") +
  theme(aspect.ratio=2/2.2, text = element_text(size = 16), axis.text= element_text(size=12)) +
  annotate("text",x=1.5,y=0.25, label="p < 0.001", size=6)
Fig5B
```

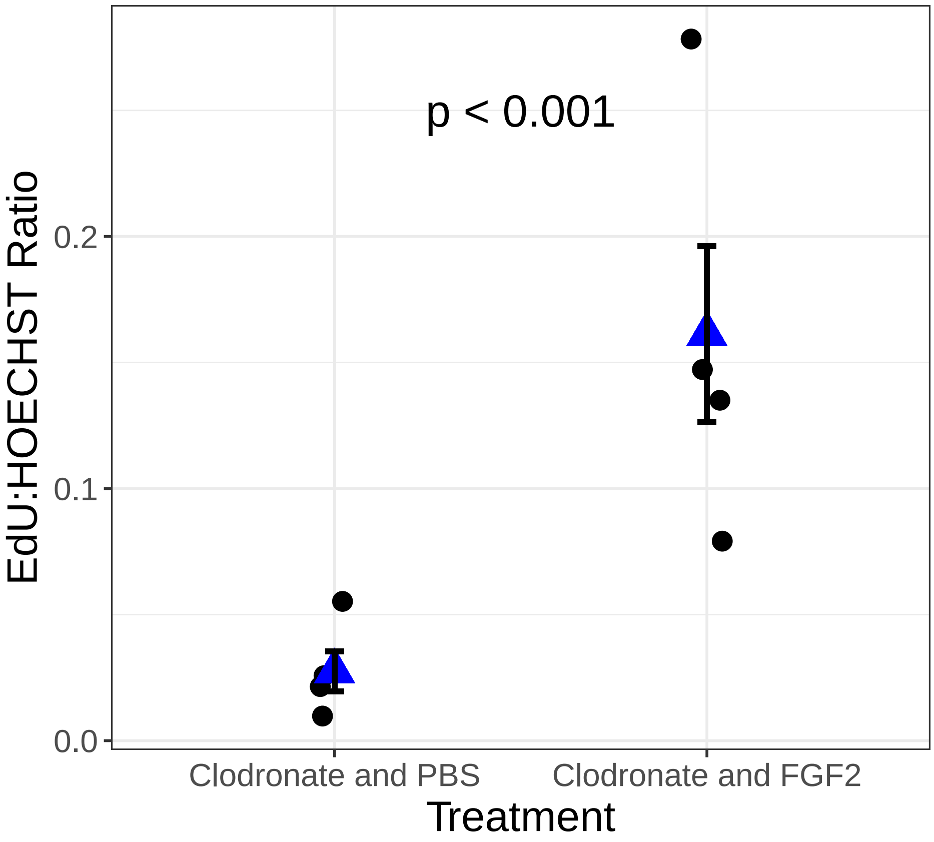


```
pdf("Fig5B.pdf")
print(Fig5B)
dev.off()
```

```
## pdf
##   2
```

Note again that the points in the plot above may vary slightly from those provided in the paper, due to jittering in the plot.

RT-qPCR Analyses (Figures 4A, 5D, and 6E)

The following analyses are for study results shown in Figures 4A, 5D, and 6E. It is again important to note that in all of these studies, we are assuming that each eye is an independent biological replicate. If there is substantial correlation between eyes from the same animal, our statistical inference results may be optimistic to some extent.

Figure 4A: Comparing IL1b using Time and Treatment (PBS vs. Clodronate) as Factors

A two-factor experiment with 8 independent replications for each of 10 groups. That is, there was a treatment factor (`PBS` vs. `Clodronate`) and a time factor (2, 4, 10, 15, and 30 days post lentectomy). However, not all replicates were taken at the same time. For times 4, 10, 15, and 30, 4 replicates were measured initially (Group `A`), then 4 replicates later (Group `B`). 8 replicates of `Time=2` were also added in Group `B`. Thus, to account for possible variation between the original experiment and the follow-up, we include a block effect (Group A vs. Group B) in our analysis. The responses measured were `Relative mRNA of FGF 10`, `Relative mRNA of IL1b`, and `Relative mRNA of FGF 2`. The response for 8 intact specimens were also measured, but we will omit this from the analysis because the treatment was not applied.

Note that the paper only shows the IL1B analysis, but we have included FGF 10 and FGF 2 here for completeness, since they were considered as well.

For these analyses, we wish to consider if there are any treatment differences within the five time levels, while accounting for any Block differences. Since we are looking for any treatment differences, following Rubin (2021) we perform disjunction testing for which we perform multiple comparisons corrections. Since the tests can reasonably be assumed to be independent, we can use the original False Discovery Rate correction (Benjamini and Hochberg 1995), which controls the proportion of false discoveries.

Benjamini, Y., and Hochberg, Y. (1995). Controlling the false discovery rate: a practical and powerful approach to multiple testing. Journal of the Royal Statistical Society Series B, 57, 289–300. http://www.jstor.org/stable/2346101.

Rubin, M. (2021). When to adjust alpha during multiple testing: A consideration of disjunction, conjunction, and individual testing. Synthese, 199(3), 10969-11000.

Data Handling

```
dataFig4A <- read.xlsx(xlsxFile = "qPCR_IL1b.xlsx", colNames = TRUE)

dataFig4A <- dataFig4A %>%
  drop_na() %>%
  mutate(Time=factor(Time),Trt=factor(Trt))
head(dataFig4A)
```

```
##          Sample Tube     FGF10      IL1b      FGF2        Trt Time Group
## 1 Clodro 2DPL 1   9b 1.7560899 1.6608666 0.7483956 Clodronate    2     B
## 2 Clodro 2DPL 2  10b 0.4166281 0.8295467 0.8444079 Clodronate    2     B
## 3 Clodro 2DPL 3  11b 0.5359373 1.2959301 0.7749010 Clodronate    2     B
## 4 Clodro 2DPL 4  12b 0.4606719 0.5599696 0.4090876 Clodronate    2     B
## 5 Clodro 2DPL 5  13b 0.7811943 2.2803654 0.7377066 Clodronate    2     B
## 6 Clodro 2DPL 6  14b 0.5436186 7.4113821 0.8155250 Clodronate    2     B
```

Exploratory Data Analysis

Now plot the data, in both the original and log scale.

```
ggp1 <- ggplot(dataFig4A, aes(x=Time,y=FGF10,color=Trt)) +
  geom_boxplot() +
  geom_point(position=position_jitterdodge())+
  facet_wrap(~Group) +
  labs(x="Time (dpl)",y="relative FGF 10 mRNA") +
  theme_bw()
ggp2 <- ggplot(dataFig4A, aes(x=Time,y=log(FGF10),color=Trt)) +
  geom_boxplot() +
  geom_point(position=position_jitterdodge())+
  facet_wrap(~Group) +
  labs(x="Time (dpl)",y="log(relative FGF 10 mRNA)") +
  theme_bw()
grid.arrange(ggp1, ggp2, nrow = 2)
```

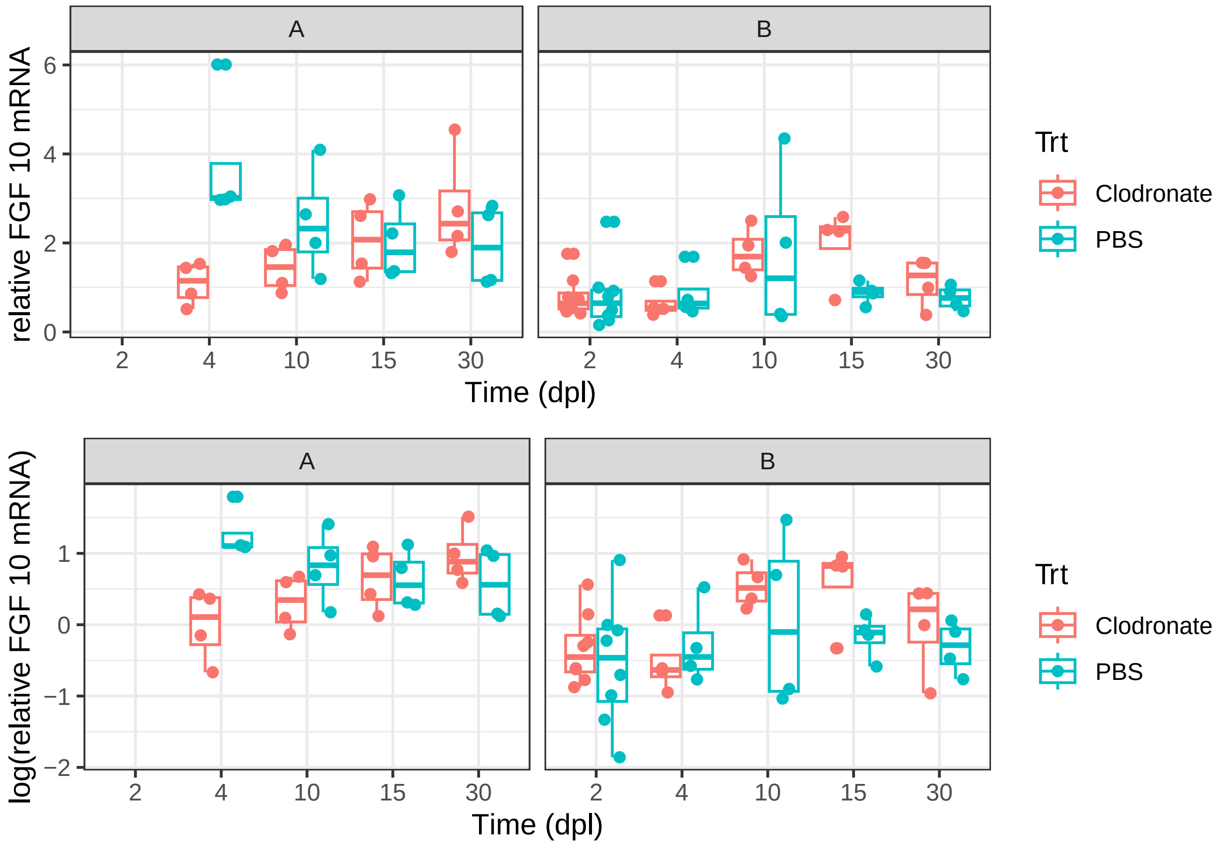


```
ggp1 <- ggplot(dataFig4A, aes(x=Time,y=IL1b,color=Trt)) +
  geom_boxplot() +
  geom_point(position=position_jitterdodge())+
  facet_wrap(~Group) +
  labs(x="Time (dpl)",y="relative IL1b mRNA") +
  theme_bw()
ggp2 <- ggplot(dataFig4A, aes(x=Time,y=log(IL1b),color=Trt)) +
```

```r
  geom_boxplot() +
  geom_point(position=position_jitterdodge())+
  facet_wrap(~Group) +
  labs(x="Time (dpl)",y="log(relative IL1b mRNA)") +
  theme_bw()
grid.arrange(ggp1, ggp2, nrow = 2)
```

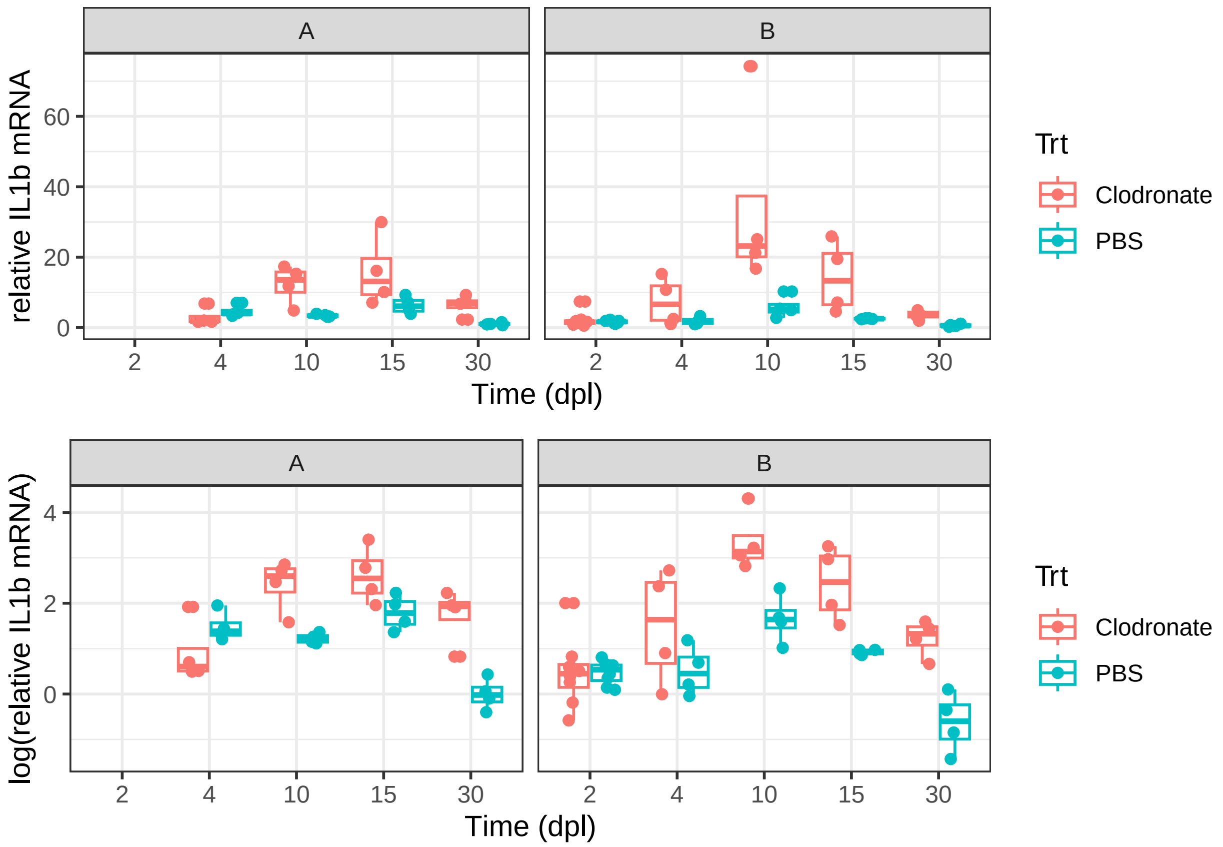


```r
ggp1 <- ggplot(dataFig4A, aes(x=Time,y=FGF2,color=Trt)) +
  geom_boxplot() +
  geom_point(position=position_jitterdodge())+
  facet_wrap(~Group) +
  labs(x="Time (dpl)",y="relative FGF 2 mRNA") +
  theme_bw()
ggp2 <- ggplot(dataFig4A, aes(x=Time,y=log(FGF2),color=Trt)) +
  geom_boxplot() +
  geom_point(position=position_jitterdodge())+
  facet_wrap(~Group) +
  labs(x="Time (dpl)",y="log(relative FGF 2 mRNA)") +
  theme_bw()
grid.arrange(ggp1, ggp2, nrow = 2)
```

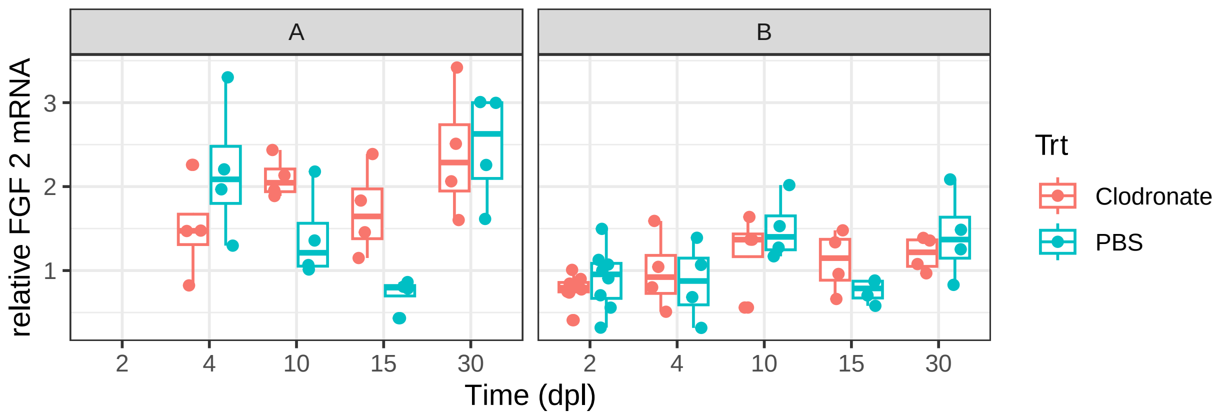

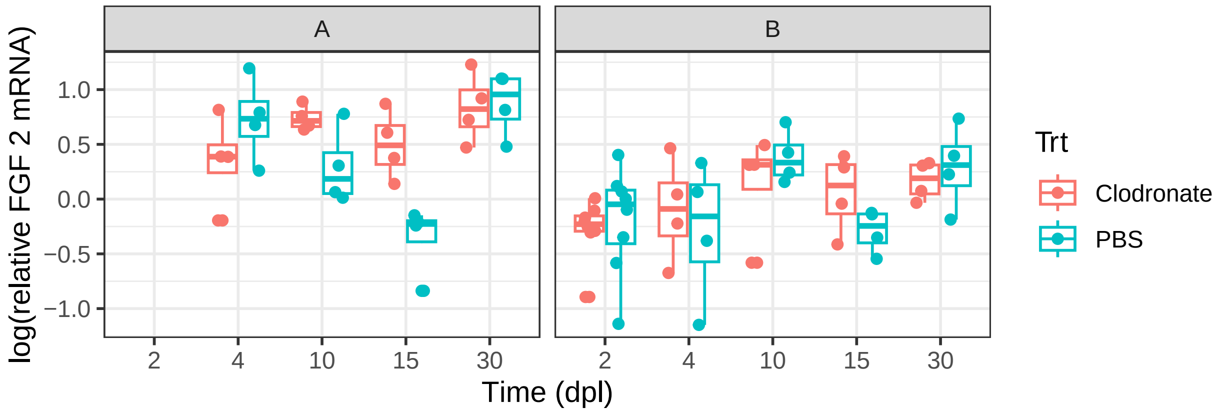

Now we will conduct a two-way ANOVA for each response.

```
fgf10.fit <- aov(FGF10~Trt+Time+Trt*Time+Group, data=dataFig4A)
autoplot(fgf10.fit)
```

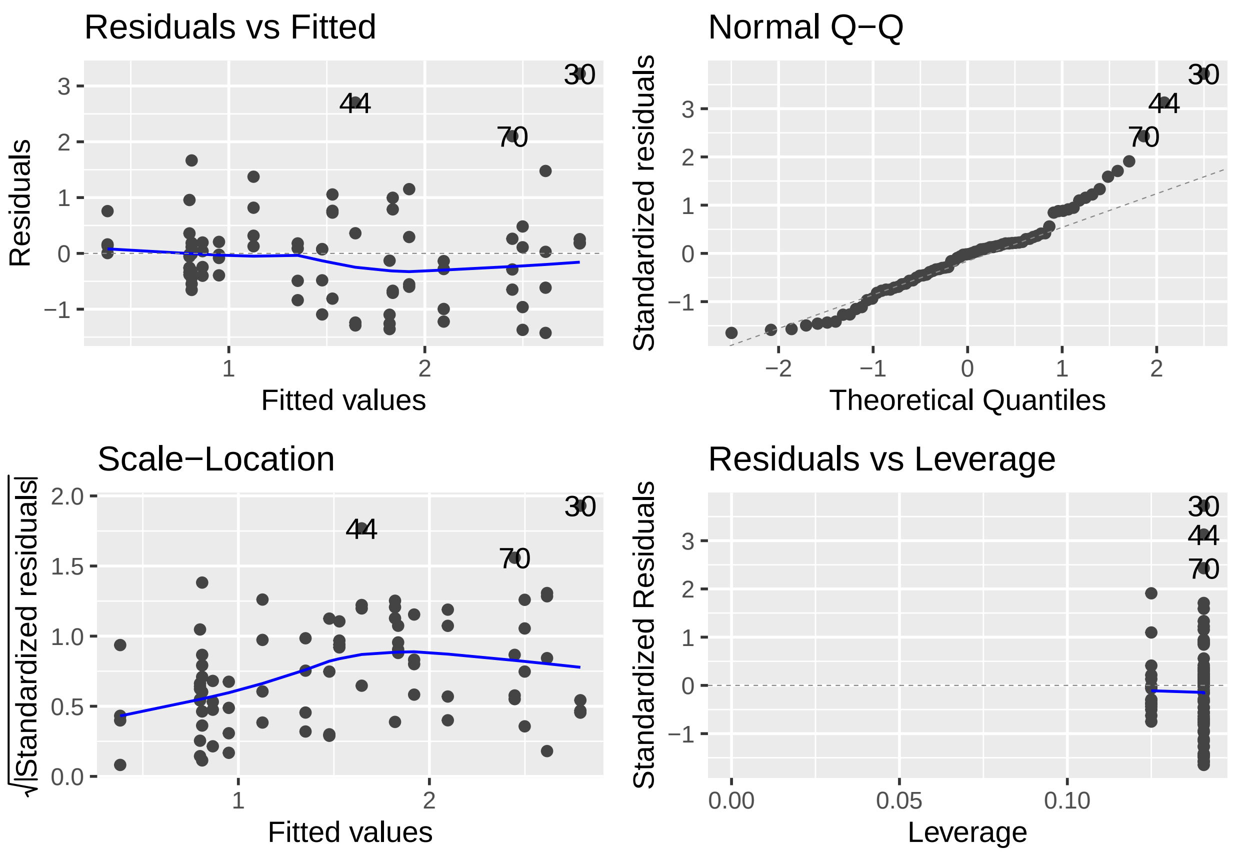


```
fgf10.logfit <- aov(log(FGF10)~Trt+Time+Trt*Time + Group, data=dataFig4A)
autoplot(fgf10.logfit)
```

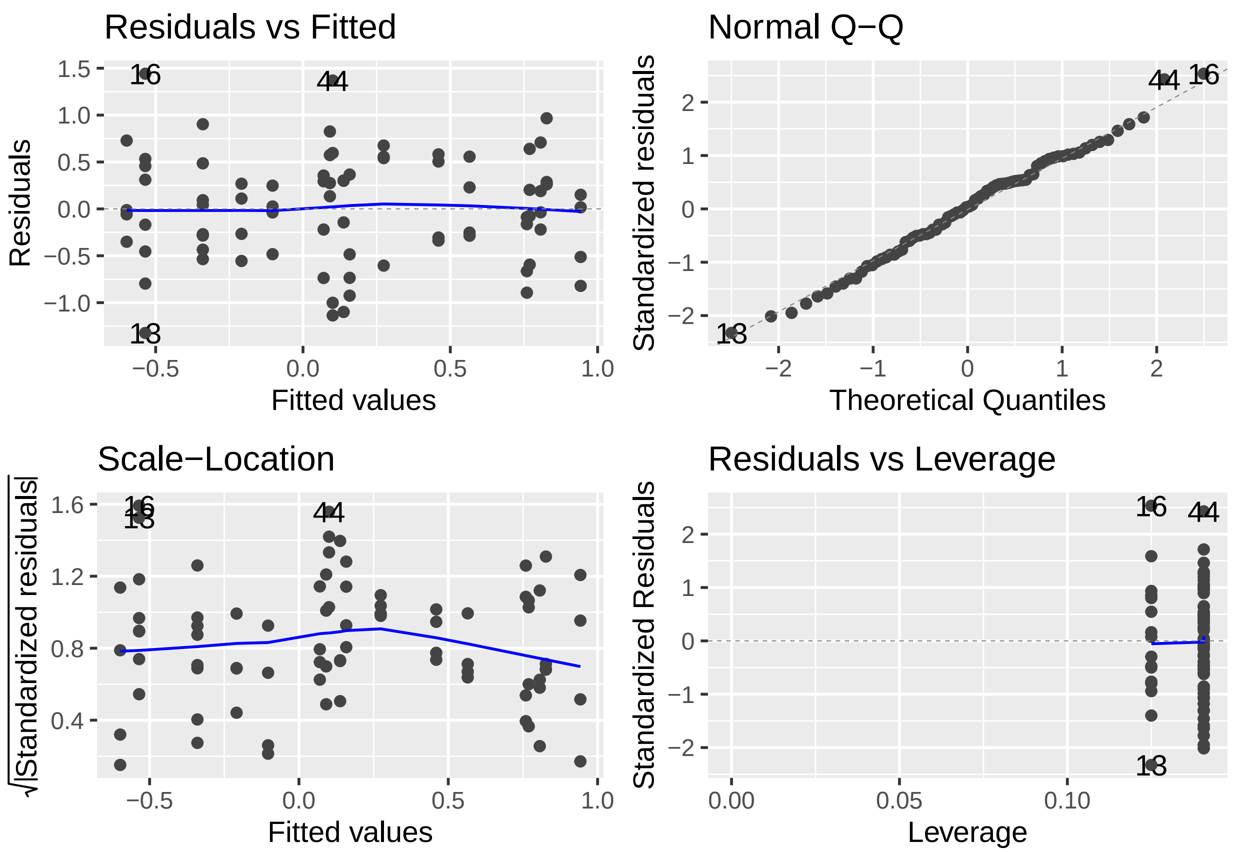


```
summary(fgf10.logfit)
```

```
##              Df Sum Sq Mean Sq F value   Pr(>F)
## Trt           1  0.019   0.019   0.050 0.823076
## Time          4  8.299   2.075   5.609 0.000572 ***
## Group         1  7.150   7.150  19.332 3.89e-05 ***
## Trt:Time      4  3.475   0.869   2.349 0.062790 .
## Residuals    69 25.520   0.370
## ---
## Signif. codes:  0 '***' 0.001 '**' 0.01 '*' 0.05 '.' 0.1 ' ' 1
```

```
IL1b.logfit <- aov(log(IL1b)~Trt+Time+Trt*Time + Group, data=dataFig4A)
autoplot(IL1b.logfit)
```

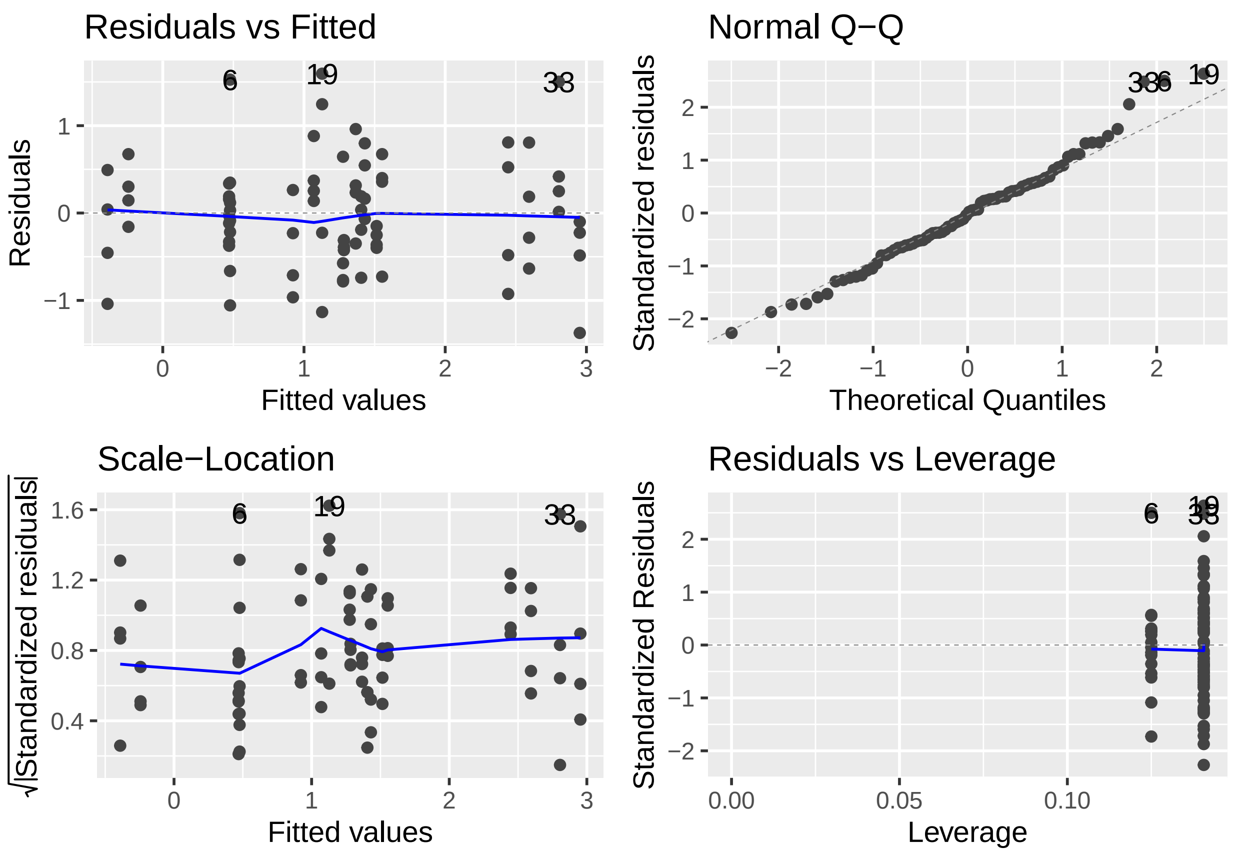


```
summary(IL1b.logfit)
```

```
##              Df Sum Sq Mean Sq F value   Pr(>F)    
## Trt           1  17.01  17.014  39.908 2.24e-08 ***
## Time          4  38.01   9.501  22.286 7.65e-12 ***
## Group         1   0.35   0.350   0.821 0.368069    
## Trt:Time      4   9.75   2.438   5.719 0.000492 ***
## Residuals    69  29.42   0.426                     
## ---
## Signif. codes:  0 '***' 0.001 '**' 0.01 '*' 0.05 '.' 0.1 ' ' 1
```

```
fgf2.logfit <- aov(log(FGF2)~Trt+Time+Trt*Time + Group, data=dataFig4A)
autoplot(fgf2.logfit)
```

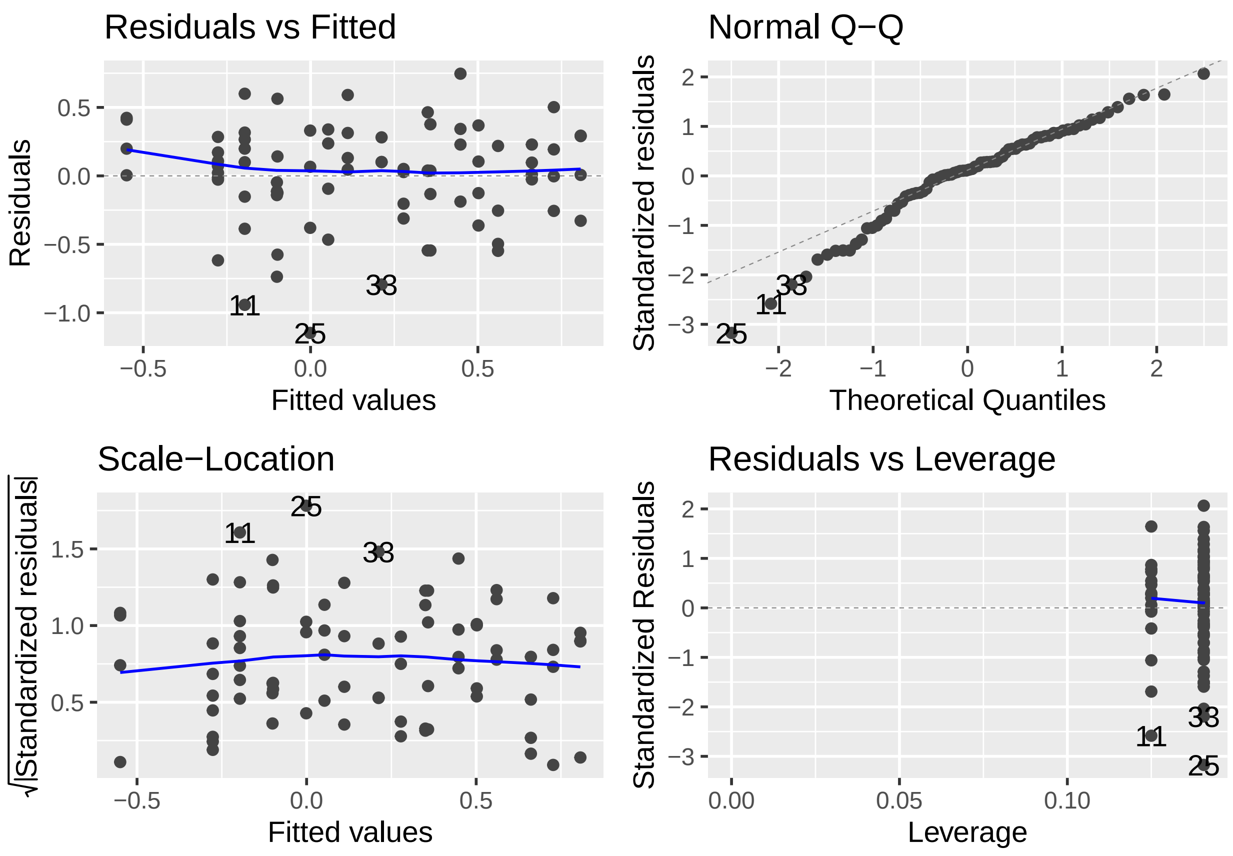


```
summary(fgf2.logfit)
```

```
##              Df Sum Sq Mean Sq F value   Pr(>F)    
## Trt           1  0.159   0.159   1.044   0.3104    
## Time          4  6.218   1.554  10.221 1.48e-06 ***
## Group         1  3.227   3.227  21.220 1.82e-05 ***
## Trt:Time      4  1.423   0.356   2.339   0.0637 .  
## Residuals    69 10.494   0.152                     
## ---
## Signif. codes:  0 '***' 0.001 '**' 0.01 '*' 0.05 '.' 0.1 ' ' 1
```

It appears better to analyze the log of the response here, because without the log it doesn't appear that the residuals have the same variance (their spread clearly changes for different groups). The interpretation is less straight-forward, but small p-values here still mean evidence of differences between groups. Overall, for all three genes once we take the log of the response, the residual vs. fitted values plots show no clear evidence of changing spread. Thus, our assumption of constant variance of each measurement is reasonable.

As specified above, we will compare treatments within each time point, correcting for the five multiple comparisons. Note that for the sake of good statistical practice we perform these comparisons whether or not there is strong evidence of a `Trt:Time` interaction or a `Trt` effect.

```
fgf10.mc <- emmeans(fgf10.logfit, pairwise ~ Trt|Time)
fgf10.mc
```

```
## $emmeans
```

```
## Time = 2:
##  Trt         emmean    SE df lower.CL upper.CL
##  Clodronate -0.0056 0.228 69 -0.46057    0.449
##  PBS        -0.2009 0.228 69 -0.65584    0.254
##
## Time = 4:
##  Trt         emmean    SE df lower.CL upper.CL
##  Clodronate -0.2641 0.215 69 -0.69310    0.165
##  PBS         0.4927 0.215 69  0.06373    0.922
##
## Time = 10:
##  Trt         emmean    SE df lower.CL upper.CL
##  Clodronate  0.4256 0.215 69 -0.00340    0.855
##  PBS         0.4351 0.215 69  0.00614    0.864
##
## Time = 15:
##  Trt         emmean    SE df lower.CL upper.CL
##  Clodronate  0.6081 0.215 69  0.17919    1.037
##  PBS         0.2310 0.215 69 -0.19792    0.660
##
## Time = 30:
##  Trt         emmean    SE df lower.CL upper.CL
##  Clodronate  0.4722 0.215 69  0.04321    0.901
##  PBS         0.1256 0.215 69 -0.30339    0.555
##
## Results are averaged over the levels of: Group
## Results are given on the log (not the response) scale.
## Confidence level used: 0.95
##
## $contrasts
## Time = 2:
##  contrast         estimate    SE df t.ratio p.value
##  Clodronate - PBS  0.19527 0.304 69   0.642  0.5229
##
## Time = 4:
##  contrast         estimate    SE df t.ratio p.value
##  Clodronate - PBS -0.75683 0.304 69  -2.489  0.0152
##
## Time = 10:
##  contrast         estimate    SE df t.ratio p.value
##  Clodronate - PBS -0.00954 0.304 69  -0.031  0.9751
##
## Time = 15:
##  contrast         estimate    SE df t.ratio p.value
##  Clodronate - PBS  0.37710 0.304 69   1.240  0.2191
##
## Time = 30:
##  contrast         estimate    SE df t.ratio p.value
##  Clodronate - PBS  0.34660 0.304 69   1.140  0.2583
##
## Results are averaged over the levels of: Group
## Results are given on the log (not the response) scale.
```

```r
fgf10.tests <- test(fgf10.mc)
fgf10.tests$contrasts$SE
```

```
## [1] 0.3040813 0.3040813 0.3040813 0.3040813 0.3040813
```

```r
# p values
fgf10.pvals <- fgf10.tests$contrasts$p.value
# adjusting p-values
p.adjust(fgf10.pvals,method="fdr")
```

```
## [1] 0.65361298 0.07615274 0.97506574 0.43050301 0.43050301
```

For the FGF 10 gene, we have a moderate amount of evidence of a treatment difference at `Time=4` (p-value 0.076). No evidence of any other treatment differences.

```r
IL1b.mc <- emmeans(IL1b.logfit, pairwise ~ Trt|Time)
IL1b.mc
```

```
## $emmeans
## Time = 2:
##  Trt        emmean    SE df lower.CL upper.CL
##  Clodronate  0.550 0.245 69   0.0620    1.039
##  PBS         0.544 0.245 69   0.0551    1.032
##
## Time = 4:
##  Trt        emmean    SE df lower.CL upper.CL
##  Clodronate  1.203 0.231 69   0.7420    1.663
##  PBS         0.996 0.231 69   0.5352    1.456
##
## Time = 10:
##  Trt        emmean    SE df lower.CL upper.CL
##  Clodronate  2.879 0.231 69   2.4184    3.339
##  PBS         1.440 0.231 69   0.9799    1.901
##
## Time = 15:
##  Trt        emmean    SE df lower.CL upper.CL
##  Clodronate  2.520 0.231 69   2.0595    2.981
##  PBS         1.357 0.231 69   0.8962    1.817
##
## Time = 30:
##  Trt        emmean    SE df lower.CL upper.CL
##  Clodronate  1.479 0.231 69   1.0187    1.940
##  PBS        -0.317 0.231 69  -0.7777    0.143
##
## Results are averaged over the levels of: Group
## Results are given on the log (not the response) scale.
## Confidence level used: 0.95
##
## $contrasts
## Time = 2:
##  contrast         estimate    SE df t.ratio p.value
##  Clodronate - PBS  0.00693 0.326 69   0.021  0.9831
```

```
##
## Time = 4:
##  contrast          estimate    SE df t.ratio p.value
##  Clodronate - PBS  0.20681 0.326 69   0.633  0.5285
##
## Time = 10:
##  contrast          estimate    SE df t.ratio p.value
##  Clodronate - PBS  1.43846 0.326 69   4.406  <.0001
##
## Time = 15:
##  contrast          estimate    SE df t.ratio p.value
##  Clodronate - PBS  1.16322 0.326 69   3.563  0.0007
##
## Time = 30:
##  contrast          estimate    SE df t.ratio p.value
##  Clodronate - PBS  1.79632 0.326 69   5.502  <.0001
##
## Results are averaged over the levels of: Group
## Results are given on the log (not the response) scale.
```

```
IL1b.tests <- test(IL1b.mc)
# p values
IL1b.tests$contrasts$SE
```

```
## [1] 0.326476 0.326476 0.326476 0.326476 0.326476
```

```
IL1b.pvals <- IL1b.tests$contrasts$p.value
# adjusting p-values
p.adjust(IL1b.pvals,method="fdr")
```

```
## [1] 9.831298e-01 6.606636e-01 9.419159e-05 1.117126e-03 2.980652e-06
```

For the IL1b gene, we have strong evidence of treatment differences at `Time=10` (p-value 0.000094), at `Time=15` (p-value 0.0011), and at `Time=30` (p-value 0.0000030). No evidence of differences at the other two time points.

```
fgf2.mc <- emmeans(fgf2.logfit, pairwise ~ Trt|Time)
fgf2.mc
```

```
## $emmeans
## Time = 2:
##  Trt         emmean    SE df lower.CL upper.CL
##  Clodronate -0.0522 0.146 69  -0.3439   0.2396
##  PBS         0.0278 0.146 69  -0.2639   0.3196
##
## Time = 4:
##  Trt         emmean    SE df lower.CL upper.CL
##  Clodronate  0.1256 0.138 69  -0.1494   0.4007
##  PBS         0.2234 0.138 69  -0.0517   0.4984
##
## Time = 10:
##  Trt         emmean    SE df lower.CL upper.CL
```

```
##   Clodronate  0.4368 0.138 69   0.1617   0.7118
##   PBS         0.3357 0.138 69   0.0607   0.6108
##
## Time = 15:
##   Trt          emmean    SE df lower.CL upper.CL
##   Clodronate  0.2773 0.138 69   0.0022   0.5523
##   PBS        -0.3251 0.138 69  -0.6002  -0.0501
##
## Time = 30:
##   Trt          emmean    SE df lower.CL upper.CL
##   Clodronate  0.5025 0.138 69   0.2274   0.7775
##   PBS         0.5827 0.138 69   0.3076   0.8577
##
## Results are averaged over the levels of: Group
## Results are given on the log (not the response) scale.
## Confidence level used: 0.95
##
## $contrasts
## Time = 2:
##   contrast          estimate    SE df t.ratio p.value
##   Clodronate - PBS   -0.0800 0.195 69  -0.410  0.6829
##
## Time = 4:
##   contrast          estimate    SE df t.ratio p.value
##   Clodronate - PBS   -0.0977 0.195 69  -0.501  0.6178
##
## Time = 10:
##   contrast          estimate    SE df t.ratio p.value
##   Clodronate - PBS    0.1011 0.195 69   0.518  0.6059
##
## Time = 15:
##   contrast          estimate    SE df t.ratio p.value
##   Clodronate - PBS    0.6024 0.195 69   3.089  0.0029
##
## Time = 30:
##   contrast          estimate    SE df t.ratio p.value
##   Clodronate - PBS   -0.0802 0.195 69  -0.411  0.6821
##
## Results are averaged over the levels of: Group
## Results are given on the log (not the response) scale.
```

```r
fgf2.tests <- test(fgf2.mc)
fgf2.tests$contrasts$SE
```

```
## [1] 0.1949886 0.1949886 0.1949886 0.1949886 0.1949886
```

```r
# p values
fgf2.pvals <- fgf2.tests$contrasts$p.value
# adjusting p-values
p.adjust(fgf2.pvals,method="fdr")
```

```
## [1] 0.68288392 0.68288392 0.68288392 0.01445287 0.68288392
```

For the FGF 2 gene, there is evidence of a treatment difference at `Time=15` (p-value 0.014), and no evidence of any other differences.

Figure 5D: Comparing Nine Gene Expressions using Time (4 vs. 10 dpl) and Treatment (Heparin vs. FGF2) as Factors

```
dataFigHepFGF2 <- read.xlsx(xlsxFile = "qPCR_HepFGF2.xlsx", colNames = TRUE)

dataFigHepFGF2 <- dataFigHepFGF2 %>%
  mutate(Time=factor(Time),Trt=factor(Trt)) %>%
  rename(CSF1R=CSFLR)
str(dataFigHepFGF2)
```

```
## 'data.frame':    32 obs. of  12 variables:
##  $ Sample: chr  "HEP 4DPL 1" "HEP 4DPL 2" "HEP 4DPL 3" "HEP 4DPL 4" ...
##  $ TGFB3 : num  1.61 1.82 1.68 1.87 1.38 ...
##  $ CSF1R : num  0.773 1.62 0.756 1.229 1.212 ...
##  $ TGFB2 : num  1.262 2.475 1.544 1.584 0.747 ...
##  $ IL1b  : num  0.543 1.617 1.688 0.974 1.247 ...
##  $ SOCS3 : num  0.922 1.12 0.421 1.475 1.188 ...
##  $ COX2  : num  0.441 1.429 0.743 1.823 0.877 ...
##  $ P53   : num  0.602 1.61 1.437 0.935 0.704 ...
##  $ CDK2  : num  0.917 1.481 1.655 1.541 0.756 ...
##  $ E2F1  : num  0.273 0.826 2.749 2.069 1.521 ...
##  $ Trt   : Factor w/ 2 levels "FGF","HEP": 2 2 2 2 2 2 2 2 1 1 ...
##  $ Time  : Factor w/ 2 levels "4","10": 1 1 1 1 1 1 1 1 1 1 ...
```

Exploratory Data Analysis

We will plot both in the original and log scale.

```
#genes <- c("TGFB3","CSF1R","TGFB2","IL1b","SOCS3","COX2", "P53","CDK2", "E2F1")
dataFigHepFGF2_L <- dataFigHepFGF2 %>% pivot_longer(2:10, names_to="Genes", values_to="Response") %>% mu

ggplot(dataFigHepFGF2_L, aes(x=Time,y=Response,color=Trt)) +
  geom_boxplot() +
  geom_point(position=position_jitterdodge()) +
  facet_wrap(~Genes) +
  theme_bw()
```

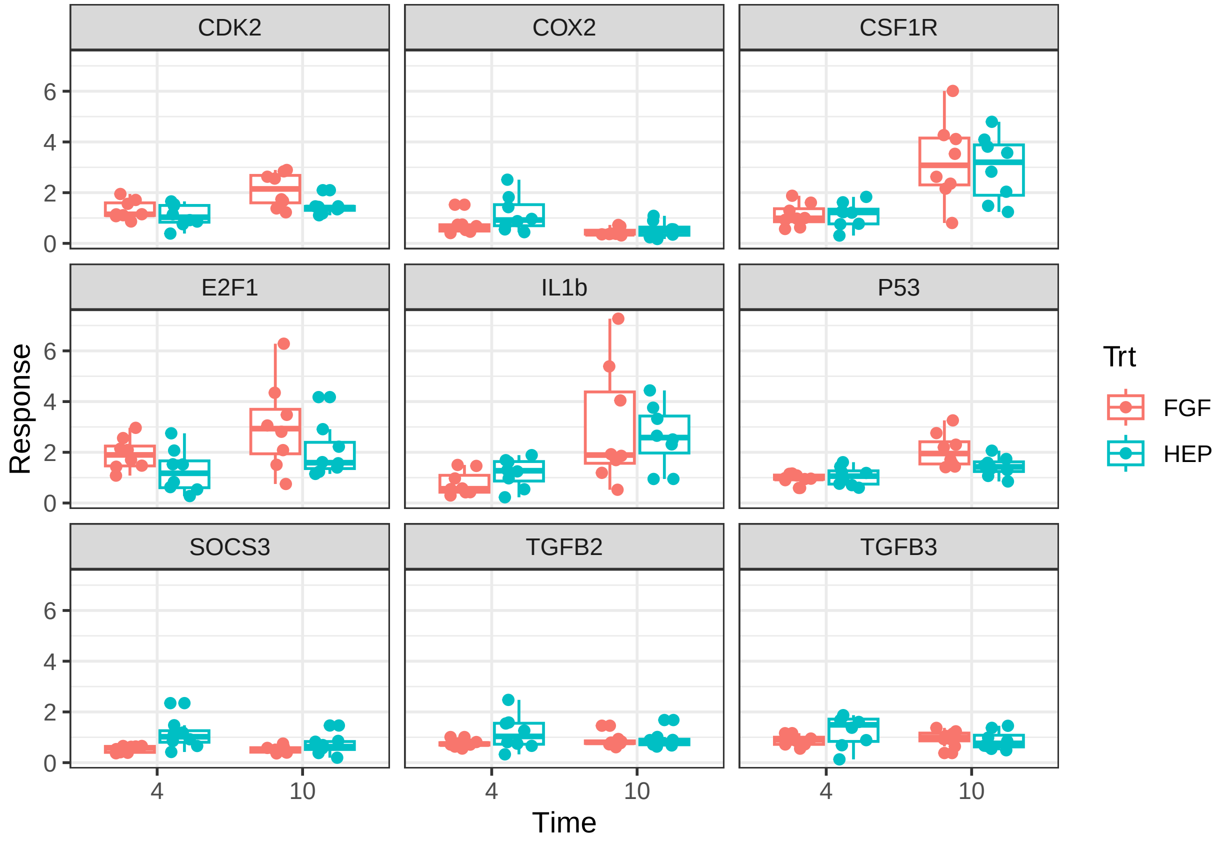


```r
ggplot(dataFigHepFGF2_L, aes(x=Time,y=log(Response),color=Trt)) +
  geom_boxplot() +
  geom_point(position=position_jitterdodge()) +
  facet_wrap(~Genes) +
  theme_bw()
```

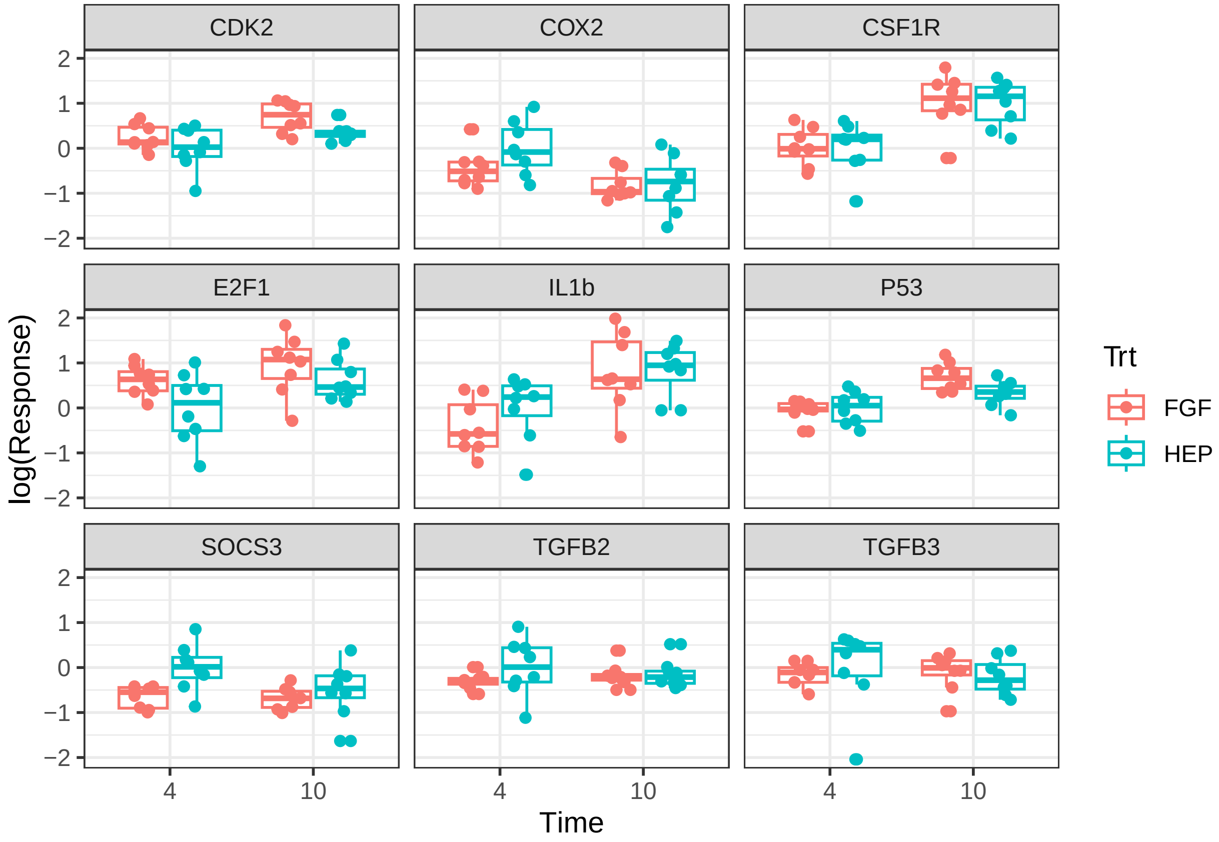

We will be fitting linear models to each of these gene expression datasets. Thus, for each dataset we make the assumption that each observation has the same variance. Neither the original data nor the logged response fits the equal variance assumption perfectly, as evidenced by more variation in some boxplots than others. However, the logged response looks better overall so we will proceed with that.

```
FigHepFGF2.logfitTGFB3 <- lm(log(Response)~Trt+Time+Trt*Time, data=dataFigHepFGF2_L[dataFigHepFGF2_L$Ger
  autoplot(FigHepFGF2.logfitTGFB3)
```

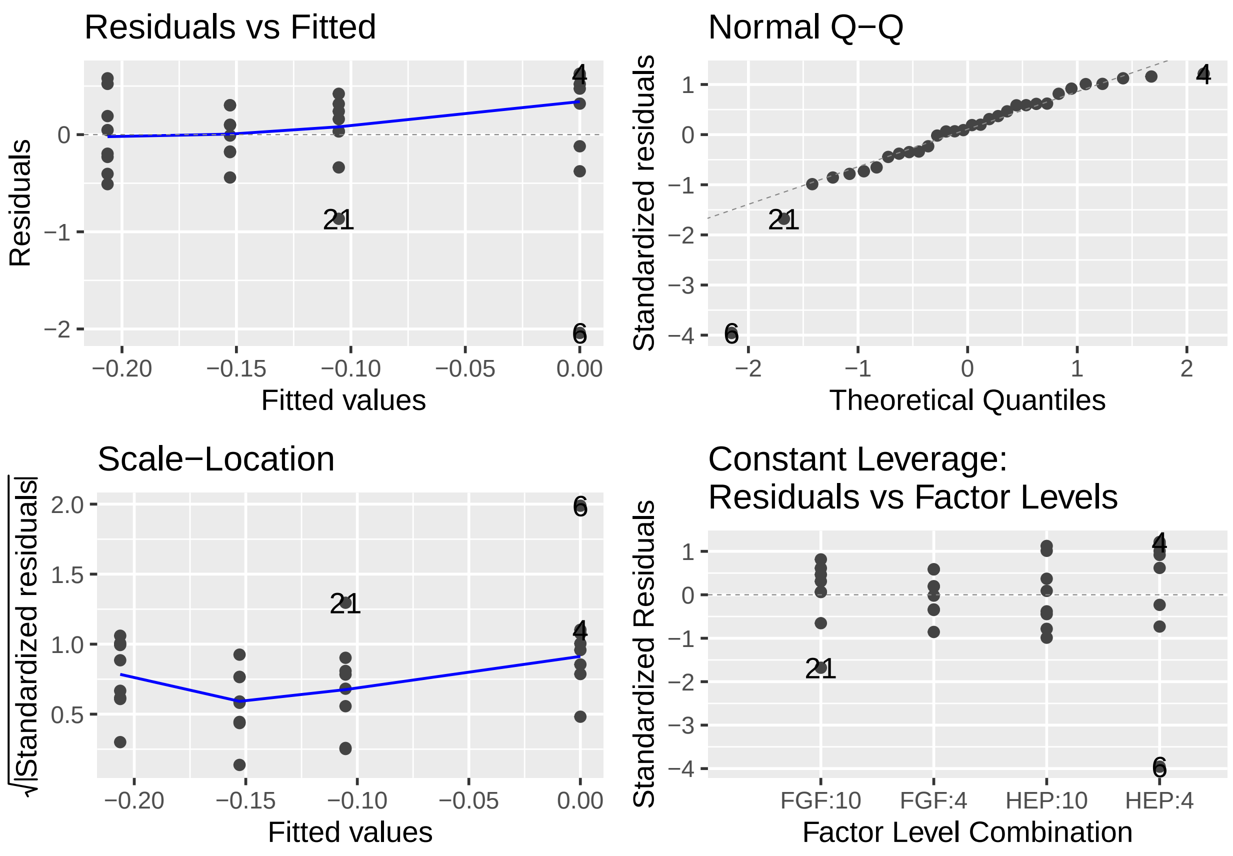


```
summary(FigHepFGF2.logfitTGFB3)
```

```
##
## Call:
## lm(formula = log(Response) ~ Trt + Time + Trt * Time, data = dataFigHepFGF2_L[dataFigHepFGF2_L$Genes
##     "TGFB3", ])
##
## Residuals:
##      Min       1Q   Median       3Q      Max
## -2.04084 -0.20457  0.07238  0.31697  0.62703
##
## Coefficients:
##               Estimate Std. Error t value Pr(>|t|)
## (Intercept)   -0.15279    0.19499  -0.784    0.440
## TrtHEP         0.15279    0.27576   0.554    0.584
## Time10         0.04751    0.27576   0.172    0.864
## TrtHEP:Time10 -0.25382    0.38998  -0.651    0.520
##
## Residual standard error: 0.5515 on 28 degrees of freedom
## Multiple R-squared:  0.02122,    Adjusted R-squared:  -0.08365
## F-statistic: 0.2023 on 3 and 28 DF,  p-value: 0.8939
```

```
FigHepFGF2.logfitCSF1R <- lm(log(Response)~Trt+Time+Trt*Time, data=dataFigHepFGF2_L[dataFigHepFGF2_L$Ge
  autoplot(FigHepFGF2.logfitCSF1R)
```

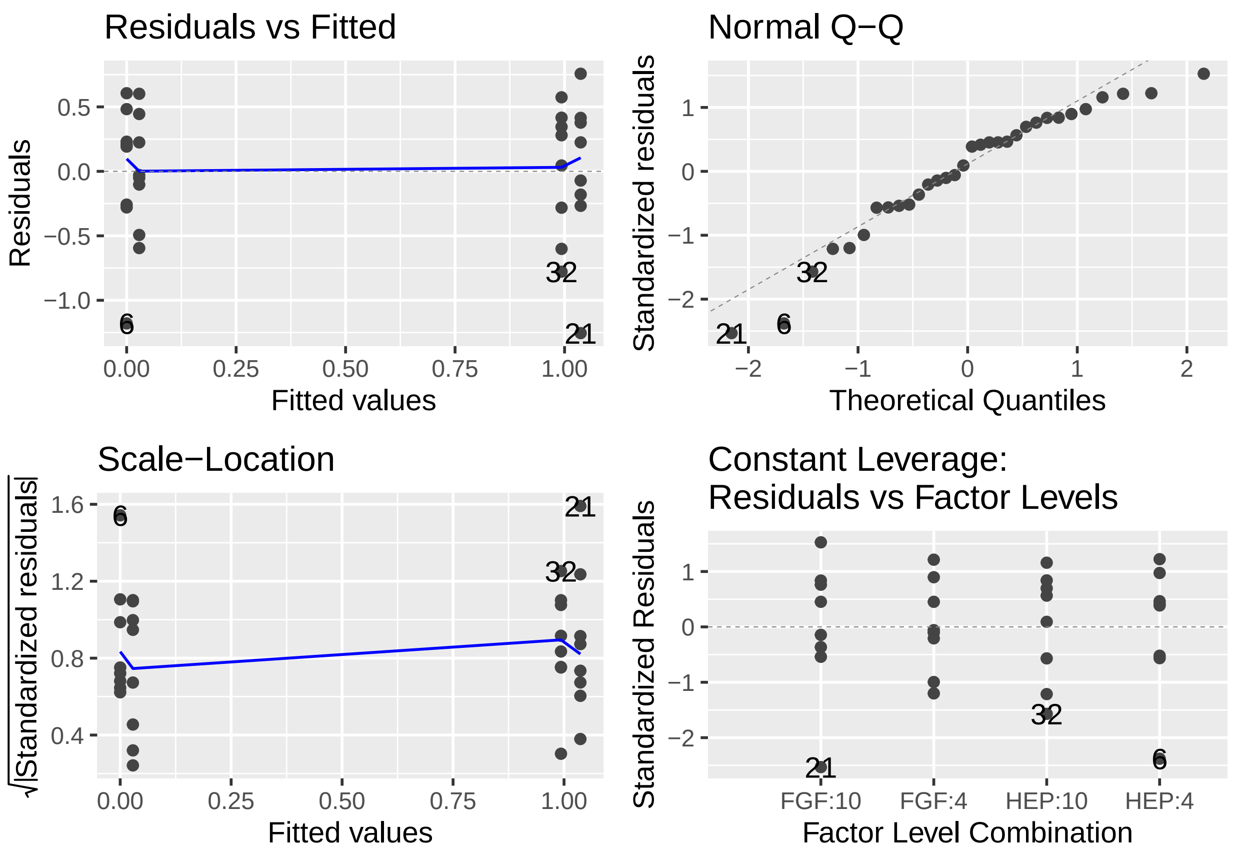


```
summary(FigHepFGF2.logfitCSF1R)
```

```
##
## Call:
## lm(formula = log(Response) ~ Trt + Time + Trt * Time, data = dataFigHepFGF2_L[dataFigHepFGF2_L$Genes
##     "CSF1R", ])
##
## Residuals:
##     Min      1Q  Median      3Q     Max
## -1.2547 -0.2704  0.1187  0.3870  0.7569
##
## Coefficients:
##               Estimate Std. Error t value Pr(>|t|)
## (Intercept)    0.02866    0.18731   0.153 0.879473
## TrtHEP        -0.02866    0.26489  -0.108 0.914603
## Time10         1.00818    0.26489   3.806 0.000705 ***
## TrtHEP:Time10 -0.01503    0.37461  -0.040 0.968286
## ---
## Signif. codes:  0 '***' 0.001 '**' 0.01 '*' 0.05 '.' 0.1 ' ' 1
##
## Residual standard error: 0.5298 on 28 degrees of freedom
## Multiple R-squared:  0.5051, Adjusted R-squared:  0.4521
## F-statistic: 9.527 on 3 and 28 DF,  p-value: 0.0001679
```

```
FigHepFGF2.logfitTGFB2 <- lm(log(Response)~Trt+Time+Trt*Time, data=dataFigHepFGF2_L[dataFigHepFGF2_L$Gen
  autoplot(FigHepFGF2.logfitTGFB2)
```

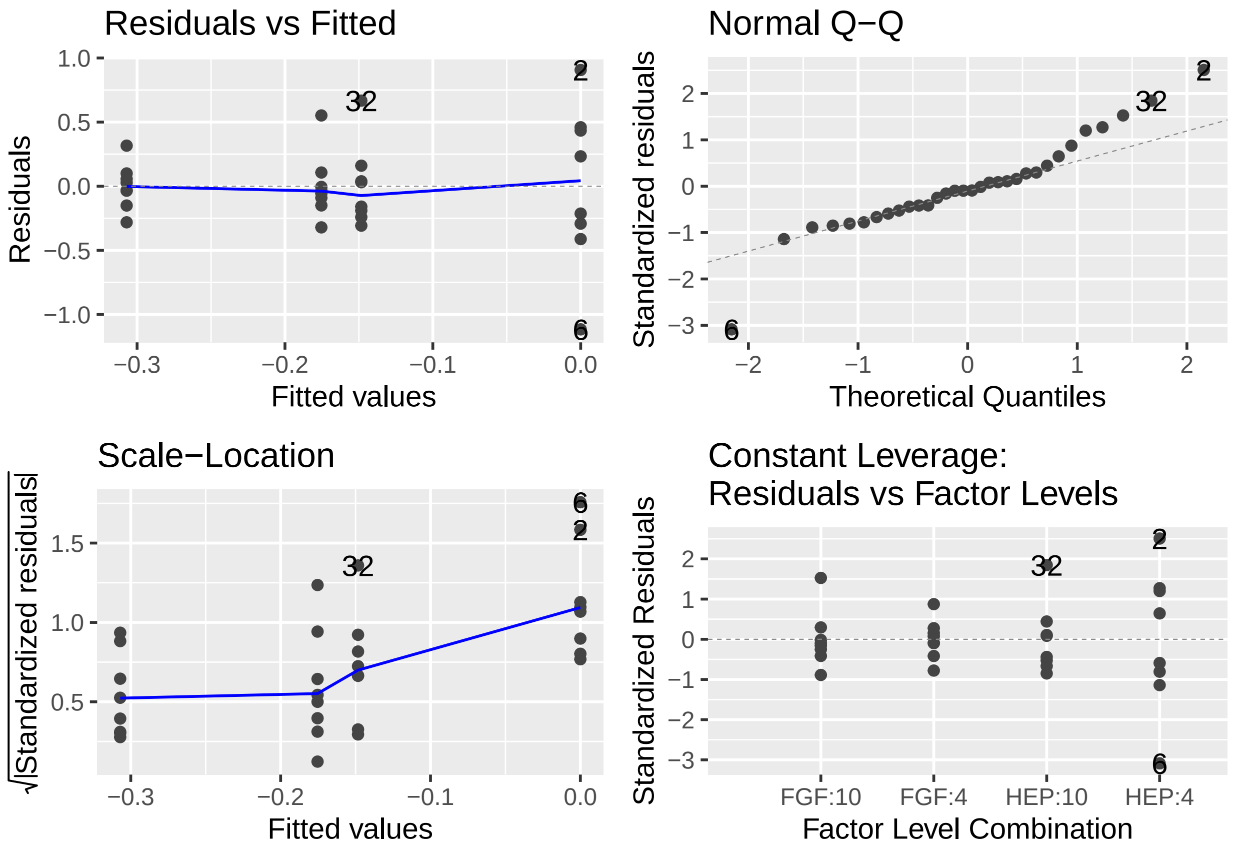


```
  summary(FigHepFGF2.logfitTGFB2)
```

```
##
## Call:
## lm(formula = log(Response) ~ Trt + Time + Trt * Time, data = dataFigHepFGF2_L[dataFigHepFGF2_L$Genes
##     "TGFB2", ])
##
## Residuals:
##      Min       1Q   Median       3Q      Max
## -1.11606 -0.19554 -0.03407  0.12009  0.90631
##
## Coefficients:
##              Estimate Std. Error t value Pr(>|t|)
## (Intercept)   -0.3071     0.1366  -2.248   0.0327 *
## TrtHEP         0.3071     0.1932   1.589   0.1232
## Time10         0.1317     0.1932   0.682   0.5011
## TrtHEP:Time10  -0.2800    0.2732  -1.025   0.3142
## ---
## Signif. codes:  0 '***' 0.001 '**' 0.01 '*' 0.05 '.' 0.1 ' ' 1
##
## Residual standard error: 0.3864 on 28 degrees of freedom
## Multiple R-squared:  0.08344,    Adjusted R-squared:  -0.01476
```

```
## F-statistic: 0.8497 on 3 and 28 DF,  p-value: 0.4785
```

```
FigHepFGF2.logfitIL1B <- lm(log(Response)~Trt+Time+Trt*Time, data=dataFigHepFGF2_L[dataFigHepFGF2_L$Gen
  autoplot(FigHepFGF2.logfitIL1B)
```

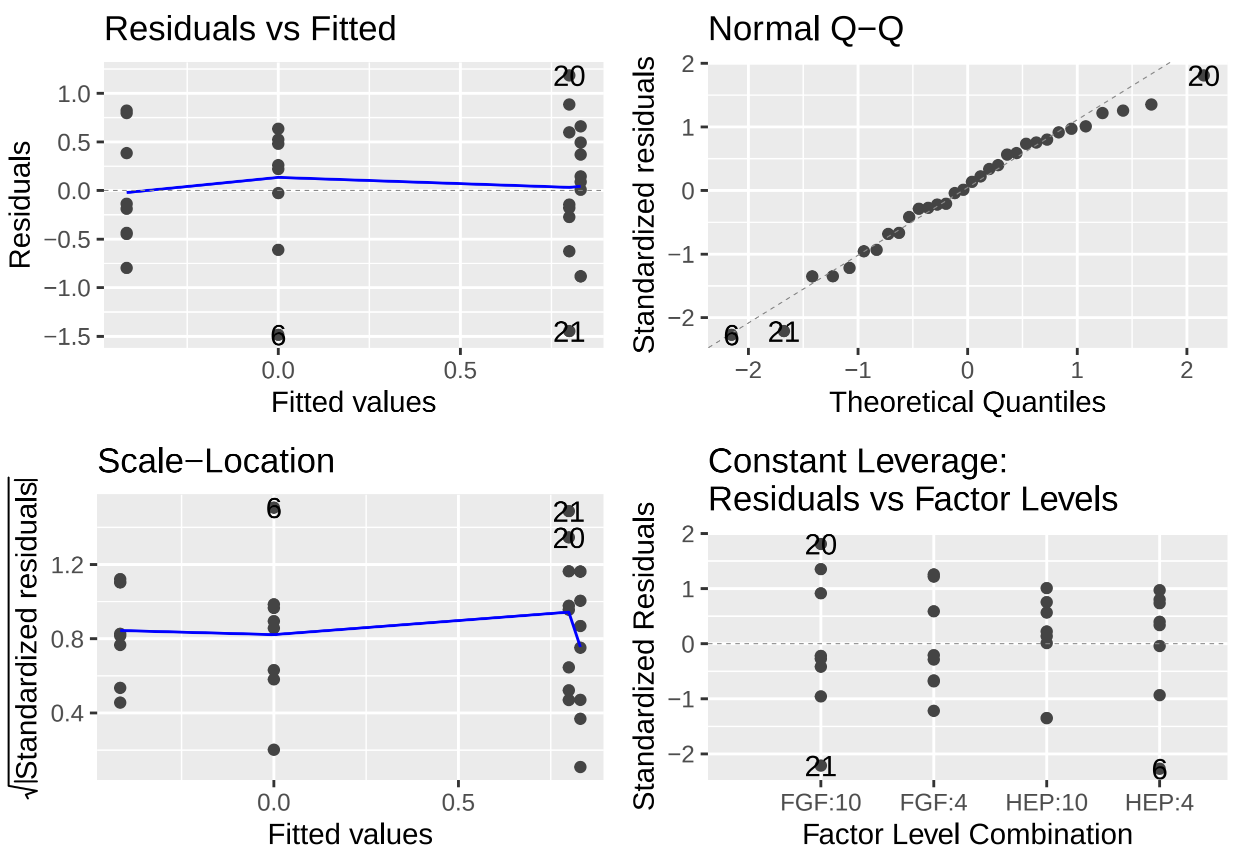


```
  summary(FigHepFGF2.logfitIL1B)
```

```
##
## Call:
## lm(formula = log(Response) ~ Trt + Time + Trt * Time, data = dataFigHepFGF2_L[dataFigHepFGF2_L$Genes
##     "IL1b", ])
##
## Residuals:
##      Min       1Q   Median       3Q      Max
## -1.48416 -0.43885  0.04845  0.50131  1.18462
##
## Coefficients:
##                Estimate Std. Error t value Pr(>|t|)
## (Intercept)     -0.4163     0.2473  -1.683  0.10348
## TrtHEP           0.4163     0.3498   1.190  0.24399
## Time10           1.2152     0.3498   3.474  0.00168 **
## TrtHEP:Time10   -0.3850     0.4946  -0.778  0.44291
## ---
## Signif. codes:  0 '***' 0.001 '**' 0.01 '*' 0.05 '.' 0.1 ' ' 1
##
```

```
## Residual standard error: 0.6995 on 28 degrees of freedom
## Multiple R-squared:  0.3982, Adjusted R-squared:  0.3337
## F-statistic: 6.175 on 3 and 28 DF,  p-value: 0.002344
```

```
FigHepFGF2.logfitSOCS3 <- lm(log(Response)~Trt+Time+Trt*Time, data=dataFigHepFGF2_L[dataFigHepFGF2_L$Ge
  autoplot(FigHepFGF2.logfitSOCS3)
```

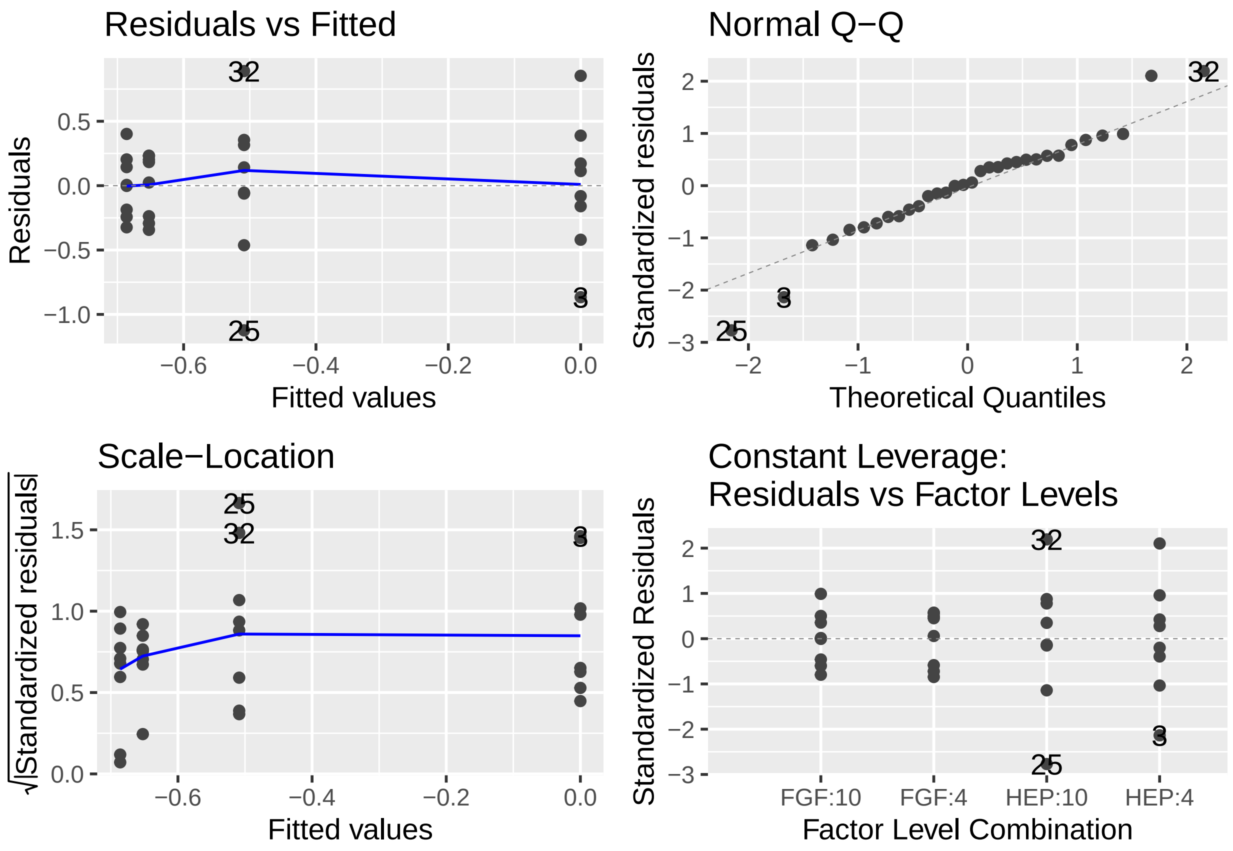


```
  summary(FigHepFGF2.logfitSOCS3)
```

```
##
## Call:
## lm(formula = log(Response) ~ Trt + Time + Trt * Time, data = dataFigHepFGF2_L[dataFigHepFGF2_L$Genes
##     "SOCS3", ])
##
## Residuals:
##     Min      1Q  Median      3Q     Max
## -1.1237 -0.2387  0.0150  0.2109  0.8898
##
## Coefficients:
##                 Estimate Std. Error t value Pr(>|t|)
## (Intercept)      -0.6524     0.1533  -4.255 0.000211 ***
## TrtHEP           0.6524     0.2168   3.009 0.005496 **
## Time10          -0.0337     0.2168  -0.155 0.877618
## TrtHEP:Time10   -0.4750     0.3066  -1.549 0.132625
## ---
```

```
## Signif. codes:  0 '***' 0.001 '**' 0.01 '*' 0.05 '.' 0.1 ' ' 1
##
## Residual standard error: 0.4336 on 28 degrees of freedom
## Multiple R-squared:  0.3146, Adjusted R-squared:  0.2411
## F-statistic: 4.284 on 3 and 28 DF,  p-value: 0.01312
```

```
FigHepFGF2.logfitCOX2 <- lm(log(Response)~Trt+Time+Trt*Time, data=dataFigHepFGF2_L[dataFigHepFGF2_L$Gene
  autoplot(FigHepFGF2.logfitCOX2)
```

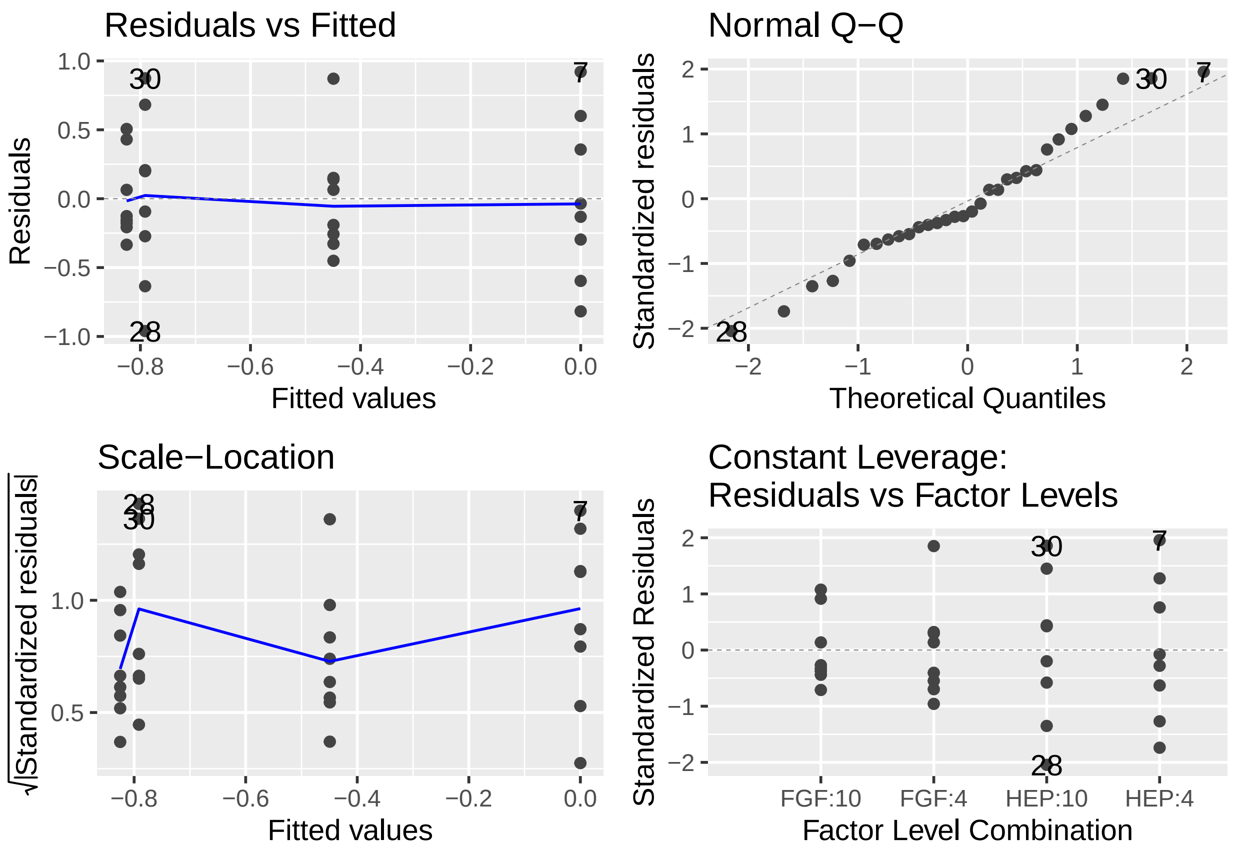


```
  summary(FigHepFGF2.logfitCOX2)
```

```
##
## Call:
## lm(formula = log(Response) ~ Trt + Time + Trt * Time, data = dataFigHepFGF2_L[dataFigHepFGF2_L$Genes
##     "COX2", ])
##
## Residuals:
##     Min      1Q  Median      3Q     Max
## -0.9611 -0.2785 -0.1101  0.2446  0.9206
##
## Coefficients:
##              Estimate Std. Error t value Pr(>|t|)
## (Intercept)   -0.4492     0.1778  -2.527   0.0174 *
## TrtHEP         0.4492     0.2514   1.787   0.0848 .
## Time10        -0.3758     0.2514  -1.495   0.1461
```

```
## TrtHEP:Time10  -0.4156     0.3555  -1.169   0.2522
## ---
## Signif. codes:  0 '***' 0.001 '**' 0.01 '*' 0.05 '.' 0.1 ' ' 1
##
## Residual standard error: 0.5028 on 28 degrees of freedom
## Multiple R-squared:  0.3332, Adjusted R-squared:  0.2617
## F-statistic: 4.663 on 3 and 28 DF,  p-value: 0.009142
```

```
FigHepFGF2.logfitP53 <- lm(log(Response)~Trt+Time+Trt*Time, data=dataFigHepFGF2_L[dataFigHepFGF2_L$Gene
  autoplot(FigHepFGF2.logfitP53)
```

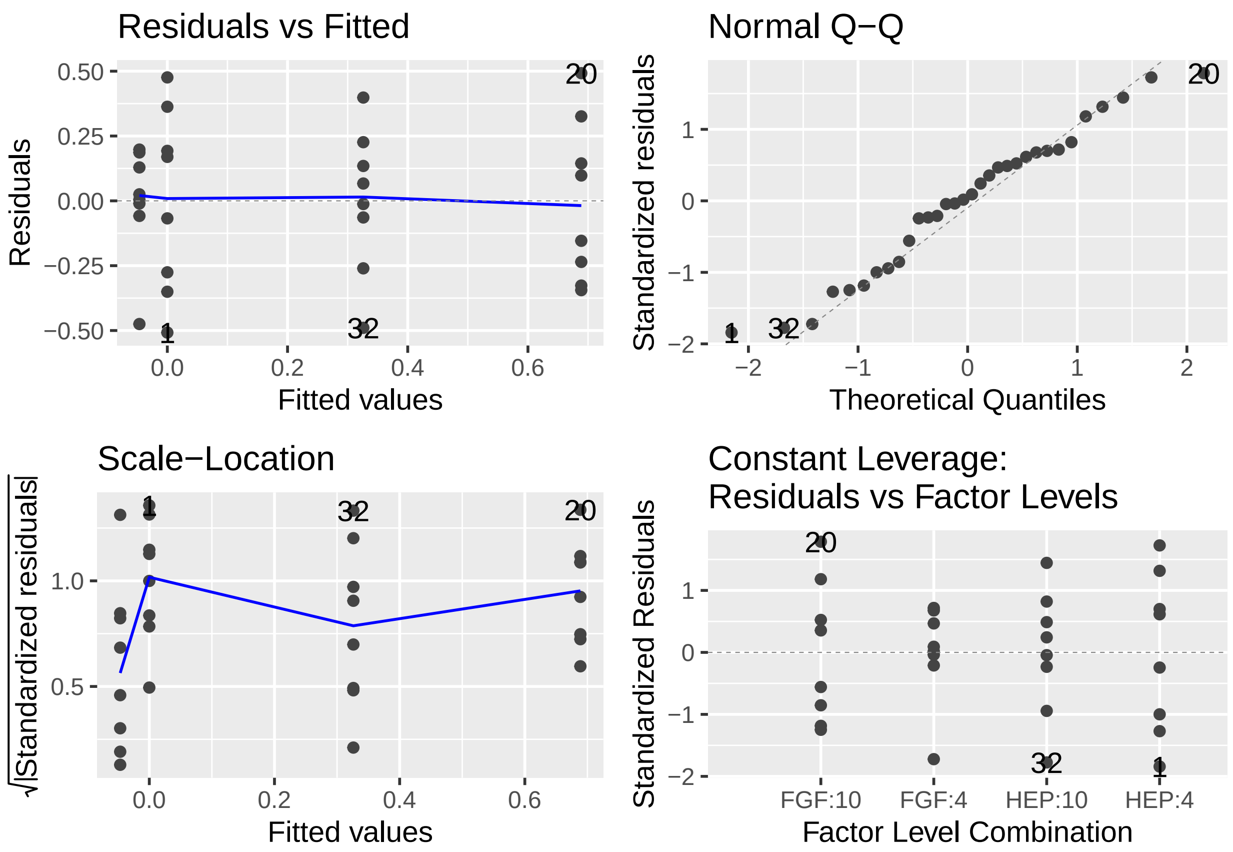


```
  summary(FigHepFGF2.logfitP53)
```

```
##
## Call:
## lm(formula = log(Response) ~ Trt + Time + Trt * Time, data = dataFigHepFGF2_L[dataFigHepFGF2_L$Genes
##     "P53", ])
##
## Residuals:
##      Min       1Q   Median       3Q      Max
## -0.50779 -0.24158  0.01489  0.18821  0.49241
##
## Coefficients:
##                Estimate Std. Error t value Pr(>|t|)
## (Intercept)    -0.0465     0.1042  -0.446   0.6589
```

```
## TrtHEP          0.0465     0.1474   0.315   0.7547
## Time10          0.7353     0.1474   4.989 2.86e-05 ***
## TrtHEP:Time10  -0.4092     0.2084  -1.963   0.0596 .
## ---
## Signif. codes:  0 '***' 0.001 '**' 0.01 '*' 0.05 '.' 0.1 ' ' 1
##
## Residual standard error: 0.2948 on 28 degrees of freedom
## Multiple R-squared:  0.534,  Adjusted R-squared:  0.4841
## F-statistic: 10.69 on 3 and 28 DF,  p-value: 7.42e-05
```

```
FigHepFGF2.logfitCDK2 <- lm(log(Response)~Trt+Time+Trt*Time, data=dataFigHepFGF2_L[dataFigHepFGF2_L$Gene
  autoplot(FigHepFGF2.logfitCDK2)
```

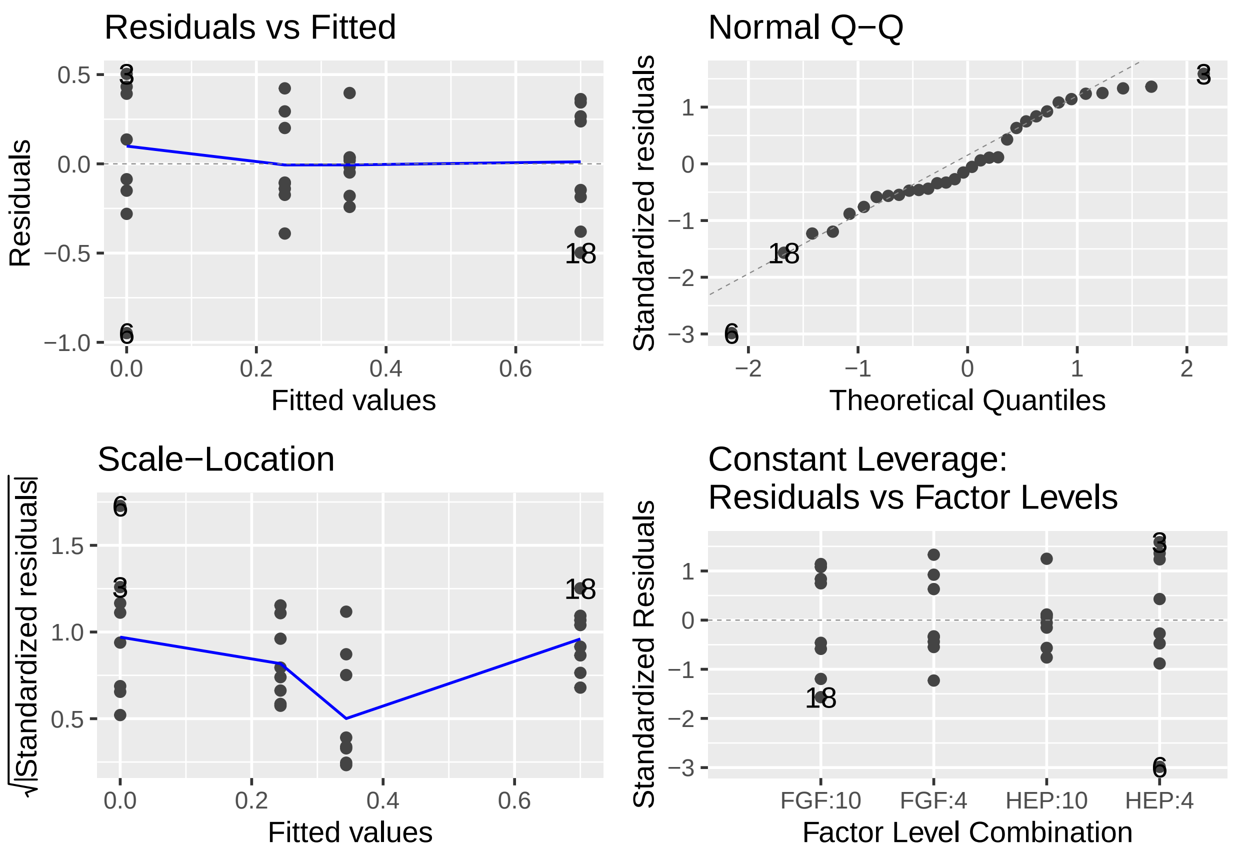


```
  summary(FigHepFGF2.logfitCDK2)
```

```
##
## Call:
## lm(formula = log(Response) ~ Trt + Time + Trt * Time, data = dataFigHepFGF2_L[dataFigHepFGF2_L$Genes
##     "CDK2", ])
##
## Residuals:
##      Min       1Q   Median       3Q      Max
## -0.94832 -0.17522 -0.03287  0.27296  0.50394
##
## Coefficients:
```

```
##                 Estimate Std. Error t value Pr(>|t|)
## (Intercept)       0.2438     0.1201   2.031   0.0519 .
## TrtHEP           -0.2438     0.1698  -1.436   0.1621
## Time10            0.4562     0.1698   2.686   0.0120 *
## TrtHEP:Time10    -0.1124     0.2402  -0.468   0.6434
## ---
## Signif. codes:  0 '***' 0.001 '**' 0.01 '*' 0.05 '.' 0.1 ' ' 1
##
## Residual standard error: 0.3397 on 28 degrees of freedom
## Multiple R-squared:  0.3854, Adjusted R-squared:  0.3196
## F-statistic: 5.853 on 3 and 28 DF,  p-value: 0.003102
```

```
FigHepFGF2.logfitE2F1 <- lm(log(Response)~Trt+Time+Trt*Time, data=dataFigHepFGF2_L[dataFigHepFGF2_L$Gene
  autoplot(FigHepFGF2.logfitE2F1)
```

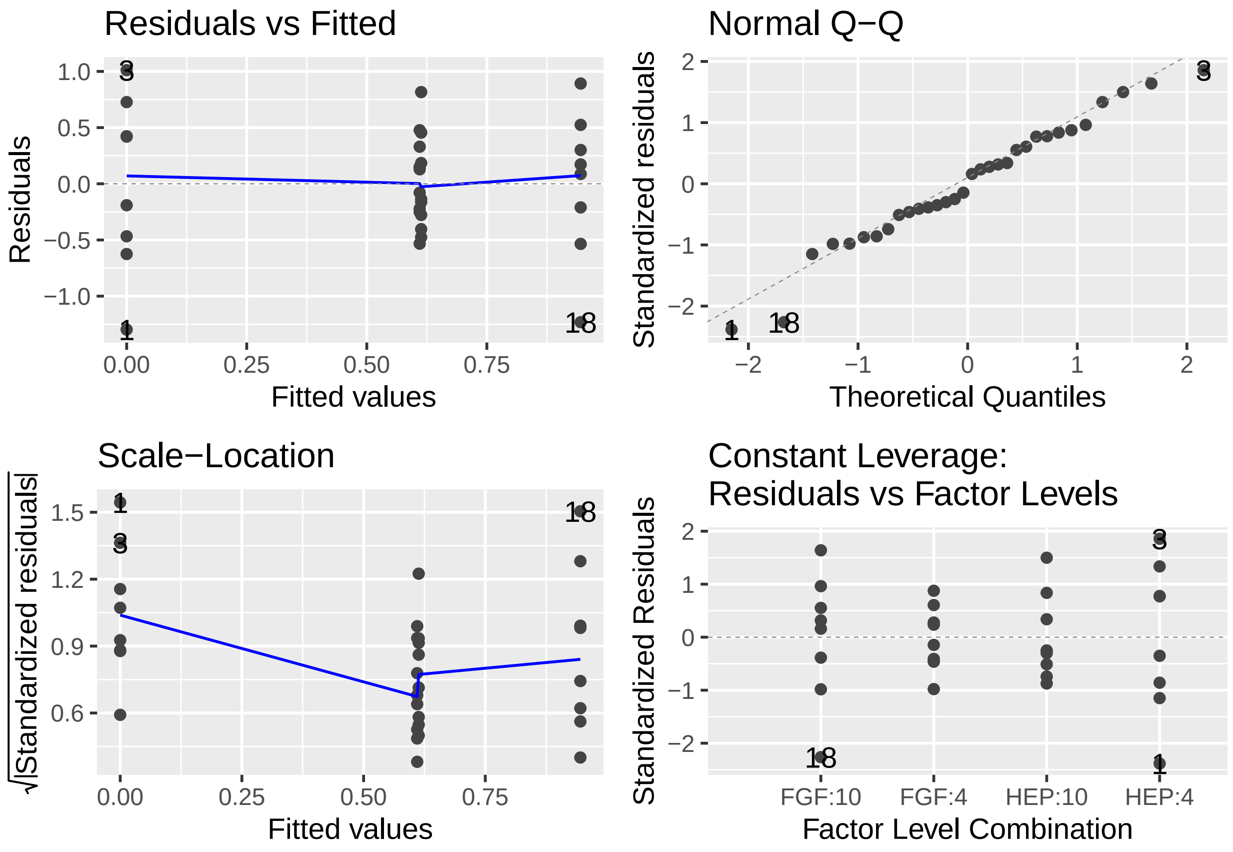


```
  summary(FigHepFGF2.logfitE2F1)
```

```
##
## Call:
## lm(formula = log(Response) ~ Trt + Time + Trt * Time, data = dataFigHepFGF2_L[dataFigHepFGF2_L$Genes
##     "E2F1", ])
##
## Residuals:
##      Min       1Q   Median       3Q      Max
## -1.29747 -0.30912  0.00412  0.42040  1.01111
```

```
##
## Coefficients:
##                Estimate Std. Error t value Pr(>|t|)
## (Intercept)      0.6102     0.2057   2.966  0.00611 **
## TrtHEP          -0.6102     0.2909  -2.097  0.04511 *
## Time10           0.3352     0.2909   1.152  0.25900
## TrtHEP:Time10    0.2781     0.4114   0.676  0.50465
## ---
## Signif. codes:  0 '***' 0.001 '**' 0.01 '*' 0.05 '.' 0.1 ' ' 1
##
## Residual standard error: 0.5818 on 28 degrees of freedom
## Multiple R-squared:  0.2824, Adjusted R-squared:  0.2055
## F-statistic: 3.672 on 3 and 28 DF,  p-value: 0.02388
```

```r
mcTGFB3 <- emmeans(FigHepFGF2.logfitTGFB3, pairwise ~ Trt|Time)
mcTGFB3
```

```
## $emmeans
## Time = 4:
##  Trt emmean    SE df lower.CL upper.CL
##  FGF -0.153 0.195 28   -0.552    0.247
##  HEP  0.000 0.195 28   -0.399    0.399
##
## Time = 10:
##  Trt emmean    SE df lower.CL upper.CL
##  FGF -0.105 0.195 28   -0.505    0.294
##  HEP -0.206 0.195 28   -0.606    0.193
##
## Results are given on the log (not the response) scale.
## Confidence level used: 0.95
##
## $contrasts
## Time = 4:
##  contrast  estimate    SE df t.ratio p.value
##  FGF - HEP   -0.153 0.276 28  -0.554  0.5839
##
## Time = 10:
##  contrast  estimate    SE df t.ratio p.value
##  FGF - HEP    0.101 0.276 28   0.366  0.7169
##
## Results are given on the log (not the response) scale.
```

```r
mcTGFB3.tests <- test(mcTGFB3)
#mcTGFB3.tests$contrasts$SE
# p values
mcTGFB3.pvals <- mcTGFB3.tests$contrasts$p.value
# adjusting p-values
p.adjust(mcTGFB3.pvals,method="fdr")
```

```
## [1] 0.7168581 0.7168581
```

```r
mcCSF1R <- emmeans(FigHepFGF2.logfitCSF1R, pairwise ~ Trt|Time)
mcCSF1R
```

```
## $emmeans
## Time = 4:
##  Trt emmean    SE df lower.CL upper.CL
##  FGF 0.0287 0.187 28   -0.355    0.412
##  HEP 0.0000 0.187 28   -0.384    0.384
##
## Time = 10:
##  Trt emmean    SE df lower.CL upper.CL
##  FGF 1.0368 0.187 28    0.653    1.421
##  HEP 0.9932 0.187 28    0.609    1.377
##
## Results are given on the log (not the response) scale.
## Confidence level used: 0.95
##
## $contrasts
## Time = 4:
##  contrast  estimate    SE df t.ratio p.value
##  FGF - HEP   0.0287 0.265 28   0.108  0.9146
##
## Time = 10:
##  contrast  estimate    SE df t.ratio p.value
##  FGF - HEP   0.0437 0.265 28   0.165  0.8702
##
## Results are given on the log (not the response) scale.
```

```r
mcCSF1R.tests <- test(mcCSF1R)
#mcCSF1R.tests$contrasts$SE
mcCSF1R.pvals <- mcCSF1R.tests$contrasts$p.value
p.adjust(mcCSF1R.pvals,method="fdr")
```

```
## [1] 0.9146027 0.9146027
```

```r
mcTGFB2 <- emmeans(FigHepFGF2.logfitTGFB2, pairwise ~ Trt|Time)
mcTGFB2
```

```
## $emmeans
## Time = 4:
##  Trt emmean    SE df lower.CL upper.CL
##  FGF -0.307 0.137 28   -0.587  -0.0272
##  HEP  0.000 0.137 28   -0.280   0.2798
##
## Time = 10:
##  Trt emmean    SE df lower.CL upper.CL
##  FGF -0.175 0.137 28   -0.455   0.1045
##  HEP -0.148 0.137 28   -0.428   0.1315
##
## Results are given on the log (not the response) scale.
## Confidence level used: 0.95
##
```

```
## $contrasts
## Time = 4:
##  contrast  estimate    SE df t.ratio p.value
##  FGF - HEP   -0.307 0.193 28  -1.589  0.1232
##
## Time = 10:
##  contrast  estimate    SE df t.ratio p.value
##  FGF - HEP   -0.027 0.193 28  -0.140  0.8898
##
## Results are given on the log (not the response) scale.
```

```
mcTGFB2.tests <- test(mcTGFB2)
#mcTGFB2.tests$contrasts$SE
mcTGFB2.pvals <- mcTGFB2.tests$contrasts$p.value
# adjusting p-values
p.adjust(mcTGFB2.pvals,method="fdr")
```

```
## [1] 0.2464464 0.8898105
```

```
mcIL1B <- emmeans(FigHepFGF2.logfitIL1B, pairwise ~ Trt|Time)
mcIL1B
```

```
## $emmeans
## Time = 4:
##  Trt emmean    SE df lower.CL upper.CL
##  FGF -0.416 0.247 28   -0.923   0.0903
##  HEP  0.000 0.247 28   -0.507   0.5066
##
## Time = 10:
##  Trt emmean    SE df lower.CL upper.CL
##  FGF  0.799 0.247 28    0.292   1.3056
##  HEP  0.830 0.247 28    0.324   1.3368
##
## Results are given on the log (not the response) scale.
## Confidence level used: 0.95
##
## $contrasts
## Time = 4:
##  contrast  estimate   SE df t.ratio p.value
##  FGF - HEP  -0.4163 0.35 28  -1.190  0.2440
##
## Time = 10:
##  contrast  estimate   SE df t.ratio p.value
##  FGF - HEP  -0.0313 0.35 28  -0.089  0.9294
##
## Results are given on the log (not the response) scale.
```

```
mcIL1B.tests <- test(mcIL1B)
#mcIL1B.tests$contrasts$SE
# p values
mcIL1B.pvals <- mcIL1B.tests$contrasts$p.value
# adjusting p-values
p.adjust(mcIL1B.pvals,method="fdr")
```

```
## [1] 0.4879765 0.9293778
```

```
mcSOCS3 <- emmeans(FigHepFGF2.logfitSOCS3, pairwise ~ Trt|Time)
mcSOCS3
```

```
## $emmeans
## Time = 4:
##  Trt emmean    SE df lower.CL upper.CL
##  FGF -0.652 0.153 28   -0.966   -0.338
##  HEP  0.000 0.153 28   -0.314    0.314
##
## Time = 10:
##  Trt emmean    SE df lower.CL upper.CL
##  FGF -0.686 0.153 28   -1.000   -0.372
##  HEP -0.509 0.153 28   -0.823   -0.195
##
## Results are given on the log (not the response) scale.
## Confidence level used: 0.95
##
## $contrasts
## Time = 4:
##  contrast  estimate    SE df t.ratio p.value
##  FGF - HEP   -0.652 0.217 28  -3.009  0.0055
##
## Time = 10:
##  contrast  estimate    SE df t.ratio p.value
##  FGF - HEP   -0.177 0.217 28  -0.818  0.4201
##
## Results are given on the log (not the response) scale.
```

```
mcSOCS3.tests <- test(mcSOCS3)
#mcSOCS3.tests$contrasts$SE
# p values
mcSOCS3.pvals <- mcSOCS3.tests$contrasts$p.value
# adjusting p-values
p.adjust(mcSOCS3.pvals,method="fdr")
```

```
## [1] 0.01099216 0.42010741
```

```
mcCOX2 <- emmeans(FigHepFGF2.logfitCOX2, pairwise ~ Trt|Time)
mcCOX2
```

```
## $emmeans
## Time = 4:
##  Trt emmean    SE df lower.CL upper.CL
##  FGF -0.449 0.178 28   -0.813  -0.0851
##  HEP  0.000 0.178 28   -0.364   0.3641
##
## Time = 10:
##  Trt emmean    SE df lower.CL upper.CL
##  FGF -0.825 0.178 28   -1.189  -0.4608
##  HEP -0.791 0.178 28   -1.155  -0.4273
```

```
##
## Results are given on the log (not the response) scale.
## Confidence level used: 0.95
##
## $contrasts
## Time = 4:
##  contrast  estimate    SE df t.ratio p.value
##  FGF - HEP  -0.4492 0.251 28  -1.787  0.0848
##
## Time = 10:
##  contrast  estimate    SE df t.ratio p.value
##  FGF - HEP  -0.0336 0.251 28  -0.134  0.8947
##
## Results are given on the log (not the response) scale.
```

```
mcCOX2.tests <- test(mcCOX2)
#mcCOX2.tests$contrasts$SE
# p values
mcCOX2.pvals <- mcCOX2.tests$contrasts$p.value
# adjusting p-values
p.adjust(mcCOX2.pvals,method="fdr")
```

```
## [1] 0.1695810 0.8946901
```

```
mcP53 <- emmeans(FigHepFGF2.logfitP53, pairwise ~ Trt|Time)
mcP53
```

```
## $emmeans
## Time = 4:
##  Trt   emmean    SE df lower.CL upper.CL
##  FGF -0.0465 0.104 28   -0.260    0.167
##  HEP  0.0000 0.104 28   -0.213    0.213
##
## Time = 10:
##  Trt   emmean    SE df lower.CL upper.CL
##  FGF  0.6888 0.104 28    0.475    0.902
##  HEP  0.3261 0.104 28    0.113    0.540
##
## Results are given on the log (not the response) scale.
## Confidence level used: 0.95
##
## $contrasts
## Time = 4:
##  contrast  estimate    SE df t.ratio p.value
##  FGF - HEP  -0.0465 0.147 28  -0.315  0.7547
##
## Time = 10:
##  contrast  estimate    SE df t.ratio p.value
##  FGF - HEP   0.3627 0.147 28   2.461  0.0203
##
## Results are given on the log (not the response) scale.
```

```r
mcP53.tests <- test(mcP53)
#mcP53.tests$contrasts$SE
# p values
mcP53.pvals <- mcP53.tests$contrasts$p.value
# adjusting p-values
p.adjust(mcP53.pvals,method="fdr")
```

```
## [1] 0.75474821 0.04057978
```

```r
mcCDK2 <- emmeans(FigHepFGF2.logfitCDK2, pairwise ~ Trt|Time)
mcCDK2
```

```
## $emmeans
## Time = 4:
##  Trt emmean   SE df lower.CL upper.CL
##  FGF  0.244 0.12 28 -0.00214    0.490
##  HEP  0.000 0.12 28 -0.24599    0.246
##
## Time = 10:
##  Trt emmean   SE df lower.CL upper.CL
##  FGF  0.700 0.12 28  0.45409    0.946
##  HEP  0.344 0.12 28  0.09784    0.590
##
## Results are given on the log (not the response) scale.
## Confidence level used: 0.95
##
## $contrasts
## Time = 4:
##  contrast  estimate   SE df t.ratio p.value
##  FGF - HEP    0.244 0.17 28   1.436  0.1621
##
## Time = 10:
##  contrast  estimate   SE df t.ratio p.value
##  FGF - HEP    0.356 0.17 28   2.098  0.0451
##
## Results are given on the log (not the response) scale.
```

```r
mcCDK2.tests <- test(mcCDK2)
#mcCDK2.tests$contrasts$SE
# p values
mcCDK2.pvals <- mcCDK2.tests$contrasts$p.value
# adjusting p-values
p.adjust(mcCDK2.pvals,method="fdr")
```

```
## [1] 0.16213125 0.09016049
```

```r
mcE2F1 <- emmeans(FigHepFGF2.logfitE2F1, pairwise ~ Trt|Time)
mcE2F1
```

```
## $emmeans
## Time = 4:
```

```
##   Trt emmean    SE df lower.CL upper.CL
##   FGF  0.610 0.206 28    0.189    1.032
##   HEP  0.000 0.206 28   -0.421    0.421
##
## Time = 10:
##   Trt emmean    SE df lower.CL upper.CL
##   FGF  0.945 0.206 28    0.524    1.367
##   HEP  0.613 0.206 28    0.192    1.035
##
## Results are given on the log (not the response) scale.
## Confidence level used: 0.95
##
## $contrasts
## Time = 4:
##   contrast  estimate    SE df t.ratio p.value
##   FGF - HEP    0.610 0.291 28   2.097  0.0451
##
## Time = 10:
##   contrast  estimate    SE df t.ratio p.value
##   FGF - HEP    0.332 0.291 28   1.142  0.2633
##
## Results are given on the log (not the response) scale.
```

```
  mcE2F1.tests <- test(mcE2F1)
  # mcE2F1.tests$contrasts$SE
  # p values
  mcE2F1.pvals <- mcE2F1.tests$contrasts$p.value
  # adjusting p-values
  p.adjust(mcE2F1.pvals,method="fdr")
```

```
## [1] 0.09022361 0.26331869
```

Conclusions for Figure 5D as follows:

- No evidence for Treatment differences for either Time for the following genes: TGFB3, CSF1R, TGFB2, IL1b, COX2 (at `Time=4`, FDR-adjusted p-value of 0.17)
- Some evidence for Treatment differences for at least one Time point for the following genes: SOCS3 (at `Time=4`, FDR-adjusted p-value of 0.01), P53 (at `Time=10`, FDR-adjusted p-value of 0.04), CDK2 (at `Time=4`, adjusted p-value of 0.16; at `Time=10`, adjusted p-value of 0.09), E2F1 (at `Time=4`, adjusted p-value of 0.09).

Figure 6E: Late Macrophage Depletion

```
dataFigLate <- read.xlsx(xlsxFile = "qPCR_Late.xlsx", colNames = TRUE)

dataFigLate <- dataFigLate %>%
  mutate(Trt=factor(Trt)) %>%
  rename(CSF1R=CSFLR)
str(dataFigLate)
```

```
## 'data.frame':    8 obs. of  11 variables:
##  $ Sample  : chr  "6h PBS S1" "6h PBS S2" "6h PBS S3" "6h PBS S4" ...
##  $ TGFB3   : num  1.075 1.677 0.746 0.744 1.337 ...
##  $ CSF1R   : num  0.883 1.234 1.148 0.799 3.609 ...
##  $ TGFB2   : num  0.831 1.346 0.885 1.01 1.505 ...
##  $ IL1b    : num  0.615 1.284 1.182 1.072 5.503 ...
##  $ Collagen: num  0.739 1.957 0.81 0.853 1.175 ...
##  $ MMP13   : num  0.643 1.933 0.845 0.953 2.605 ...
##  $ MMP3    : num  0.361 3.331 0.883 0.942 4.681 ...
##  $ FGF2    : num  0.851 1.333 1.005 0.877 0.907 ...
##  $ MMP9    : num  0.533 1.932 1.07 0.906 1.45 ...
##  $ Trt     : Factor w/ 2 levels "CLO","PBS": 2 2 2 2 1 1 1 1
```

```r
p.IL1b <- ggplot(dataFigLate, aes(x=Trt,y=IL1b)) +
  geom_jitter(width=.05,height=0)+
  #geom_point()+
  theme_bw()
p.CSF1R <- ggplot(dataFigLate, aes(x=Trt,y=CSF1R)) +
  geom_jitter(width=.05,height=0)+
  #geom_point()+
  theme_bw()
p.FGF2 <- ggplot(dataFigLate, aes(x=Trt,y=FGF2)) +
  geom_jitter(width=.05,height=0)+
  #geom_point()+
  theme_bw()
p.TGFB2 <- ggplot(dataFigLate, aes(x=Trt,y=TGFB2)) +
  geom_jitter(width=.05,height=0)+
  #geom_point()+
  theme_bw()
p.TGFB3 <- ggplot(dataFigLate, aes(x=Trt,y=TGFB3)) +
  geom_jitter(width=.05,height=0)+
  #geom_point()+
  theme_bw()
p.Coll <- ggplot(dataFigLate, aes(x=Trt,y=Collagen)) +
  geom_jitter(width=.05,height=0)+
  #geom_point()+
  theme_bw()
p.MMP3 <- ggplot(dataFigLate, aes(x=Trt,y=MMP3)) +
  geom_jitter(width=.05,height=0)+
  #geom_point()+
  theme_bw()
p.MMP9 <- ggplot(dataFigLate, aes(x=Trt,y=MMP9)) +
  geom_jitter(width=.05,height=0)+
  #geom_point()+
  theme_bw()
p.MMP13 <- ggplot(dataFigLate, aes(x=Trt,y=MMP13)) +
  geom_jitter(width=.05,height=0)+
  #geom_point()+
  theme_bw()

ggarrange(p.IL1b,p.CSF1R,p.FGF2,p.TGFB2,p.TGFB3,p.Coll,p.MMP3,p.MMP9,p.MMP13, ncol=3, nrow=3)
```

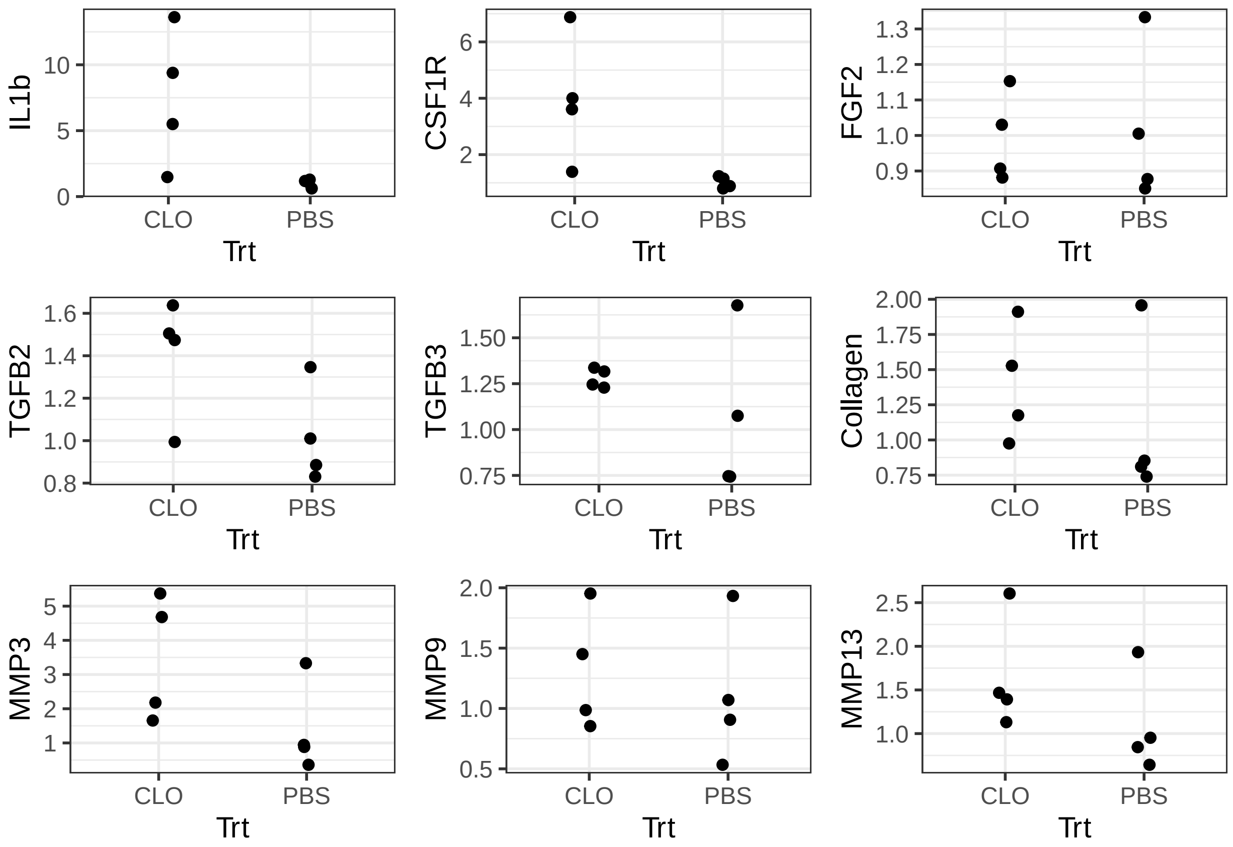


```
p.IL1b.l <- ggplot(dataFigLate, aes(x=Trt,y=log(IL1b))) +
  geom_jitter(width=.05,height=0)+
  #geom_point()+
  theme_bw()
p.CSF1R.l <- ggplot(dataFigLate, aes(x=Trt,y=log(CSF1R))) +
  geom_jitter(width=.05,height=0)+
  #geom_point()+
  theme_bw()
p.FGF2.l <- ggplot(dataFigLate, aes(x=Trt,y=log(FGF2))) +
  geom_jitter(width=.05,height=0)+
  #geom_point()+
  theme_bw()
p.TGFB2.l <- ggplot(dataFigLate, aes(x=Trt,y=log(TGFB2))) +
  geom_jitter(width=.05,height=0)+
  #geom_point()+
  theme_bw()
p.TGFB3.l <- ggplot(dataFigLate, aes(x=Trt,y=log(TGFB3))) +
  geom_jitter(width=.05,height=0)+
  #geom_point()+
  theme_bw()
p.Coll.l <- ggplot(dataFigLate, aes(x=Trt,y=log(Collagen))) +
  geom_jitter(width=.05,height=0)+
  #geom_point()+
  theme_bw()
p.MMP3.l <- ggplot(dataFigLate, aes(x=Trt,y=log(MMP3))) +
  geom_jitter(width=.05,height=0)+
```

```
  #geom_point()+
  theme_bw()
p.MMP9.l <- ggplot(dataFigLate, aes(x=Trt,y=log(MMP9))) +
  geom_jitter(width=.05,height=0)+
  #geom_point()+
  theme_bw()
p.MMP13.l <- ggplot(dataFigLate, aes(x=Trt,y=log(MMP13))) +
  geom_jitter(width=.05,height=0)+
  #geom_point()+
  theme_bw()

ggarrange(p.IL1b.l,p.CSF1R.l,p.FGF2.l,p.TGFB2.l,p.TGFB3.l,p.Col1.l,p.MMP3.l,p.MMP9.l,p.MMP13.l, ncol=3,
```

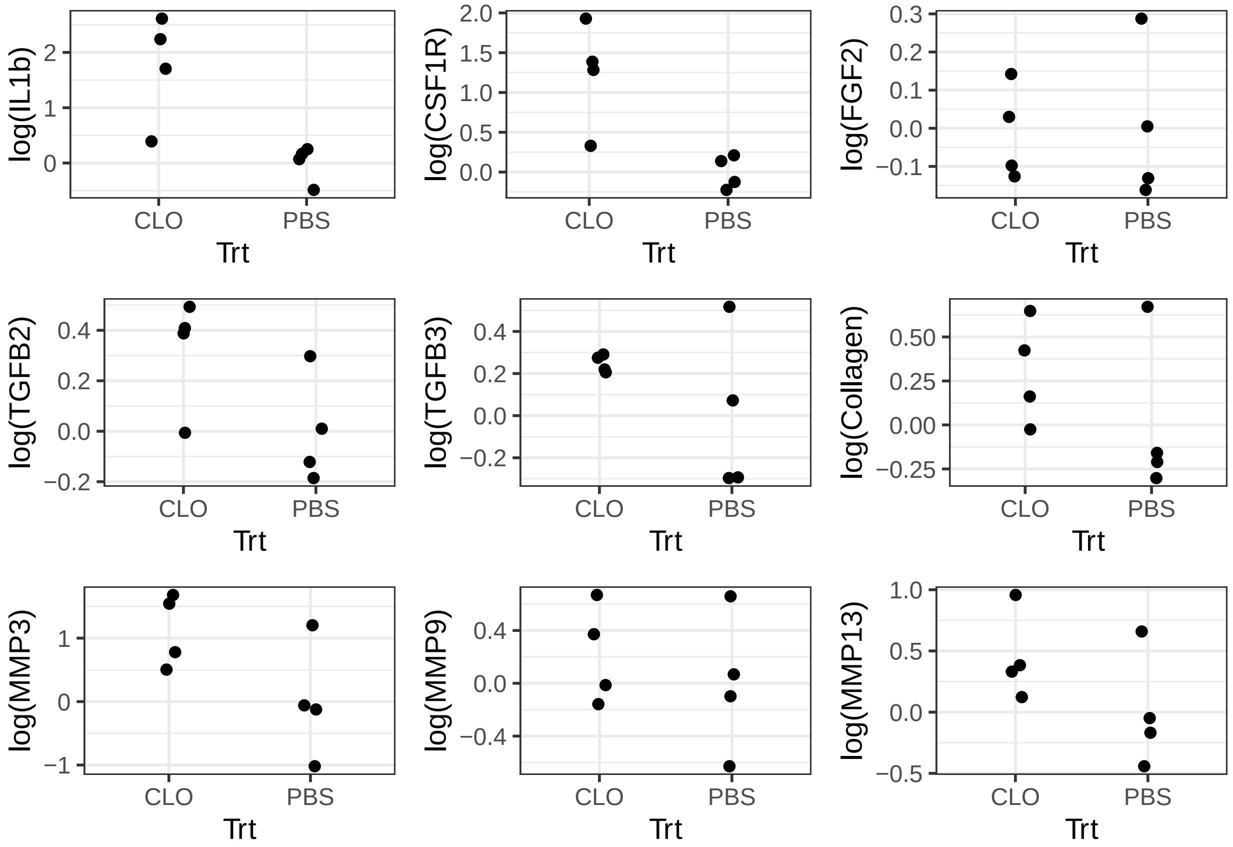


```
dataFigLate_L <- dataFigLate %>% pivot_longer(2:10, names_to="Genes", values_to="Response") %>% mutate(

# Summarize estimates and SE's
group_stats <- dataFigLate_L %>%
  group_by(Genes,Trt) %>%
  summarize(Est=mean(log(Response)), SE=sd(log(Response))/sqrt(n()))
```

```
## `summarise()` has grouped output by 'Genes'. You can override using the
## `.groups` argument.
```

```r
group_stats
```

```
## # A tibble: 18 x 4
## # Groups:   Genes [9]
##    Genes    Trt          Est     SE
##    <fct>    <fct>      <dbl>  <dbl>
##  1 Collagen CLO     3.02e- 1 0.148
##  2 Collagen PBS     6.11e-16 0.226
##  3 CSF1R    CLO     1.23e+ 0 0.332
##  4 CSF1R    PBS    -1.73e-17 0.104
##  5 FGF2     CLO    -1.30e- 2 0.0619
##  6 FGF2     PBS     1.78e-17 0.102
##  7 IL1b     CLO     1.74e+ 0 0.485
##  8 IL1b     PBS     6.52e-16 0.166
##  9 MMP13    CLO     4.49e- 1 0.179
## 10 MMP13    PBS     1.73e-17 0.235
## 11 MMP3     CLO     1.13e+ 0 0.287
## 12 MMP3     PBS    -1.04e-17 0.457
## 13 MMP9     CLO     2.17e- 1 0.188
## 14 MMP9     PBS    -2.43e-17 0.265
## 15 TGFB2    CLO     3.21e- 1 0.111
## 16 TGFB2    PBS    -5.25e-17 0.107
## 17 TGFB3    CLO     2.48e- 1 0.0207
## 18 TGFB3    PBS     6.14e-16 0.193
```

```r
FigLate.logfit1 <- t.test(log(Response)~Trt,data=dataFigLate_L[dataFigLate_L$Genes=="TGFB3",])
FigLate.logfit1
```

```
##
##  Welch Two Sample t-test
##
## data:  log(Response) by Trt
## t = 1.2777, df = 3.0689, p-value = 0.2895
## alternative hypothesis: true difference in means between group CLO and group PBS is not equal to 0
## 95 percent confidence interval:
##  -0.3615931  0.8570929
## sample estimates:
## mean in group CLO mean in group PBS
##      2.477499e-01      6.140921e-16
```

```r
FigLate.logfit2 <- t.test(log(Response)~Trt,data=dataFigLate_L[dataFigLate_L$Genes=="CSF1R",])
FigLate.logfit2
```

```
##
##  Welch Two Sample t-test
##
## data:  log(Response) by Trt
## t = 3.5388, df = 3.5795, p-value = 0.02886
## alternative hypothesis: true difference in means between group CLO and group PBS is not equal to 0
## 95 percent confidence interval:
##  0.2189226 2.2450678
## sample estimates:
```

```
## mean in group CLO mean in group PBS
##      1.231995e+00     -1.734723e-17
```

```r
FigLate.logfit2$stderr
```

```
## [1] 0.3481437
```

```r
FigLate.logfit3 <- t.test(log(Response)~Trt,data=dataFigLate_L[dataFigLate_L$Genes=="TGFB2",])
FigLate.logfit3
```

```
##
##  Welch Two Sample t-test
##
## data:  log(Response) by Trt
## t = 2.0764, df = 5.9911, p-value = 0.08322
## alternative hypothesis: true difference in means between group CLO and group PBS is not equal to 0
## 95 percent confidence interval:
##  -0.05741036  0.69929150
## sample estimates:
## mean in group CLO mean in group PBS
##      3.209406e-01     -5.247539e-17
```

```r
FigLate.logfit3$stderr
```

```
## [1] 0.1545684
```

```r
FigLate.logfit4 <- t.test(log(Response)~Trt,data=dataFigLate_L[dataFigLate_L$Genes=="IL1b",])
FigLate.logfit4
```

```
##
##  Welch Two Sample t-test
##
## data:  log(Response) by Trt
## t = 3.3845, df = 3.6939, p-value = 0.03129
## alternative hypothesis: true difference in means between group CLO and group PBS is not equal to 0
## 95 percent confidence interval:
##  0.2642399 3.2092863
## sample estimates:
## mean in group CLO mean in group PBS
##      1.736763e+00      6.522560e-16
```

```r
FigLate.logfit4$stderr
```

```
## [1] 0.5131492
```

```r
FigLate.logfit5 <- t.test(log(Response)~Trt,data=dataFigLate_L[dataFigLate_L$Genes=="Collagen",])
FigLate.logfit5
```

```
##
##  Welch Two Sample t-test
##
## data:  log(Response) by Trt
## t = 1.1198, df = 5.1666, p-value = 0.3121
## alternative hypothesis: true difference in means between group CLO and group PBS is not equal to 0
## 95 percent confidence interval:
##  -0.3845547  0.9884871
## sample estimates:
## mean in group CLO mean in group PBS
##      3.019662e-01      6.106227e-16
```

```
FigLate.logfit5$stderr
```

```
## [1] 0.2696632
```

```
FigLate.logfit6 <- t.test(log(Response)~Trt,data=dataFigLate_L[dataFigLate_L$Genes=="MMP13",])
FigLate.logfit6
```

```
##
##  Welch Two Sample t-test
##
## data:  log(Response) by Trt
## t = 1.5219, df = 5.6036, p-value = 0.1823
## alternative hypothesis: true difference in means between group CLO and group PBS is not equal to 0
## 95 percent confidence interval:
##  -0.2852685  1.1826595
## sample estimates:
## mean in group CLO mean in group PBS
##      4.486955e-01      1.734723e-17
```

```
FigLate.logfit6$stderr
```

```
## [1] 0.2948252
```

```
FigLate.logfit7 <- t.test(log(Response)~Trt,data=dataFigLate_L[dataFigLate_L$Genes=="MMP3",])
FigLate.logfit7
```

```
##
##  Welch Two Sample t-test
##
## data:  log(Response) by Trt
## t = 2.088, df = 5.0487, p-value = 0.09059
## alternative hypothesis: true difference in means between group CLO and group PBS is not equal to 0
## 95 percent confidence interval:
##  -0.2564114  2.5100850
## sample estimates:
## mean in group CLO mean in group PBS
##      1.126837e+00     -1.040834e-17
```

```r
FigLate.logfit7$stderr
```

```
## [1] 0.5396693
```

```r
FigLate.logfit8 <- t.test(log(Response)~Trt,data=dataFigLate_L[dataFigLate_L$Genes=="FGF2",])
FigLate.logfit8
```

```
##
##  Welch Two Sample t-test
##
## data:  log(Response) by Trt
## t = -0.10833, df = 4.9323, p-value = 0.918
## alternative hypothesis: true difference in means between group CLO and group PBS is not equal to 0
## 95 percent confidence interval:
##  -0.3219373  0.2960021
## sample estimates:
## mean in group CLO mean in group PBS
##     -1.296756e-02      1.778092e-17
```

```r
FigLate.logfit8$stderr
```

```
## [1] 0.1196989
```

```r
FigLate.logfit9 <- t.test(log(Response)~Trt,data=dataFigLate_L[dataFigLate_L$Genes=="MMP9",])
FigLate.logfit9
```

```
##
##  Welch Two Sample t-test
##
## data:  log(Response) by Trt
## t = 0.66915, df = 5.4021, p-value = 0.5309
## alternative hypothesis: true difference in means between group CLO and group PBS is not equal to 0
## 95 percent confidence interval:
##  -0.5991035  1.0336842
## sample estimates:
## mean in group CLO mean in group PBS
##      2.172904e-01     -2.428613e-17
```

```r
FigLate.logfit9$stderr
```

```
## [1] 0.3247282
```

Conclusions for Fig 6E:

- No clear evidence of Treatment differences for the following genes: TGFB3, Collagen, MMP13, FGF2, MMP9.
- Solid evidence of treatment differences: CSF1R (p-value 0.029); IL1b (p-value 0.031).
- Weak evidence of treatment differences: TGFB2 (0.083), MMP3 (0.091)

Eye Diameters (Figure 7B)

Three animals (six eyes) under each condition were observed at 100 days post lentectomy and diameters measured. Given that eye diameter is likely correlated within animal, we simply average the Left and Right diameters for each animal and perform a two-sample t-test.

```
data100DPL <- read.xlsx(xlsxFile = "Eye_Diameters.xlsx", colNames = TRUE)
glimpse(data100DPL)
```

```
## Rows: 36
## Columns: 8
## $ Sample           <dbl> 1, 1, 1, 2, 2, 2, 3, 3, 3, 1, 1, 1, 2, 2, 2, 3, 3, 3,~
## $ Treatment        <chr> "CLO", "CLO", "CLO", "CLO", "CLO", "CLO", "CLO", "CLO~
## $ Eye              <chr> "R", "R", "R", "R", "R", "R", "R", "R", "R", "L", "L"~
## $ Sample.ID        <chr> "CLO 1R", "CLO 1R", "CLO 1R", "CLO 2R", "CLO 2R", "CL~
## $ Measurement.Area <chr> "Beginning", "Middle", "End", "Beginning", "Middle", ~
## $ Cross.Section    <dbl> 125, 250, 375, 125, 250, 375, 125, 250, 375, 125, 250~
## $ Length           <dbl> 214, 311, 259, 223, 339, 310, 159, 299, 275, 216, 324~
## $ Notes            <chr> NA, NA, NA, NA, NA, NA, NA, NA, NA, NA, NA, NA, NA, N~
```

```
data3 <- data100DPL %>%
  group_by(Sample,Treatment) %>%
  summarize(Avg.Diam = mean(Length)) %>%
  mutate(Avg.Diam.microm = Avg.Diam*1200/500) # transform to micrometers
```

```
## `summarise()` has grouped output by 'Sample'. You can override using the
## `.groups` argument.
```

```
data3
```

```
## # A tibble: 6 x 4
## # Groups:   Sample [3]
##   Sample Treatment Avg.Diam Avg.Diam.microm
##    <dbl> <chr>        <dbl>           <dbl>
## 1      1 CLO           270.            647.
## 2      1 PBS           348.            834.
## 3      2 CLO           275             660
## 4      2 PBS           365             876
## 5      3 CLO           257.            617.
## 6      3 PBS           324.            778
```

```
ggplot(data3, aes(x=Treatment,y=Avg.Diam.microm)) +
  geom_jitter(width=.05,height=0)+
  theme_bw()
```

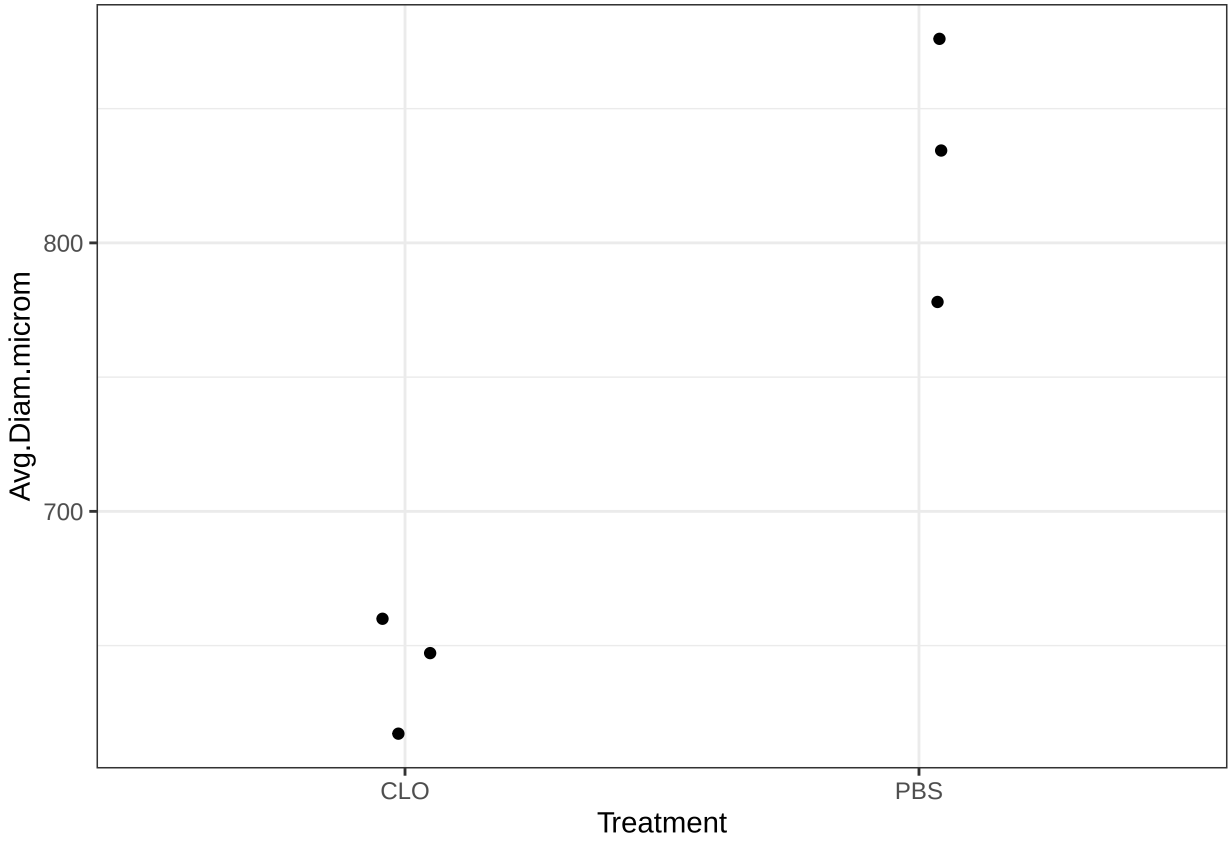


```
t.test(Avg.Diam.microm~Treatment,data=data3,var.equal=FALSE)
```

```
##
##  Welch Two Sample t-test
##
## data:  Avg.Diam.microm by Treatment
## t = -6.0448, df = 2.7674, p-value = 0.0114
## alternative hypothesis: true difference in means between group CLO and group PBS is not equal to 0
## 95 percent confidence interval:
##  -291.85463  -84.14537
## sample estimates:
## mean in group CLO mean in group PBS
##          641.4667          829.4667
```

```
group_stats <- data3 %>%
  group_by(Treatment) %>%
  summarize(Est=mean(Avg.Diam.microm), SE=sd(Avg.Diam.microm)/sqrt(n()))

group_stats
```

```
## # A tibble: 2 x 3
##   Treatment   Est    SE
##   <chr>     <dbl> <dbl>
## 1 CLO        641.  12.7
## 2 PBS        829.  28.4
```

Plot for paper:

```r
glimpse(data3)
```

```
## Rows: 6
## Columns: 4
## Groups: Sample [3]
## $ Sample          <dbl> 1, 1, 2, 2, 3, 3
## $ Treatment       <chr> "CLO", "PBS", "CLO", "PBS", "CLO", "PBS"
## $ Avg.Diam        <dbl> 269.6667, 347.6667, 275.0000, 365.0000, 257.1667, 324.~
## $ Avg.Diam.microm <dbl> 647.2, 834.4, 660.0, 876.0, 617.2, 778.0
```

```r
ggplot(data3, aes(x=Treatment,y=Avg.Diam.microm)) +
  geom_jitter(width=.05,height=0, size=3)+
  geom_point(x="CLO", y=group_stats$Est[1], color="blue", shape=17,size=5)+
  geom_point(x="PBS", y=group_stats$Est[2], color="blue",shape=17,size=5)+  theme_bw() +
  theme_bw() +
  geom_errorbar(aes(x="CLO", ymin=group_stats$Est[1]-group_stats$SE[1], ymax=group_stats$Est[1]+group_st
  geom_errorbar(aes(x="PBS", ymin=group_stats$Est[2]-group_stats$SE[2], ymax=group_stats$Est[2]+group_st
  ylab(expression(paste("Iris Diameter (", mu, "m)"))) +
  scale_x_discrete(breaks=c("CLO", "PBS"),
                   labels=c("Clodronate liposome", "PBS liposome")) +
  theme(aspect.ratio=2/2.2, text = element_text(size = 16), axis.text= element_text(size=12)) +
  annotate("text",x=1.5,y=925, label="p = 0.01", size=6)
```

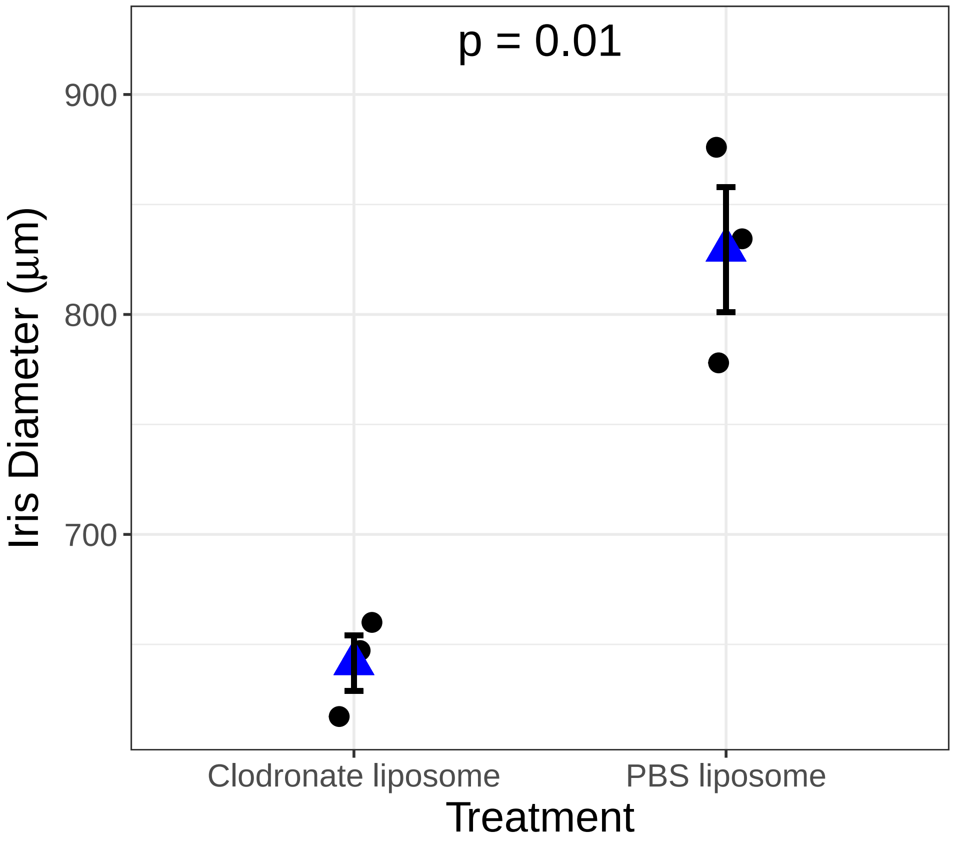

Supplement: Supplementary file 1 — Additional file 1: Statistical analysis. [file 13287_2024_3740_MOESM1_ESM.pdf]
