# Supplementary material for: Macrophages modulate fibrosis during newt lens regeneration
Source: Stem Cell Res Ther. 2024 May 14;15:141. doi: 10.1186/s13287-024-03740-1 (PMC11094960; doi:10.1186/s13287-024-03740-1)
Supplement: Supplementary file 2 — Additional file 2: Fig S1. mpeg1:GFP transgenic newts enable the in vivo labeling of macrophages. A, D, G, J, M Representative fluorescence images of sections of 5-month-old mpeg1:GFP newts showing presence of eGFP+ cells in the tail, trunk and head sections. B Anti-GFP immunofluorescence staining. C Merge of mpeg1:GFP endogenous fluorescence, anti-GFP and Hoechst. E, H F4/80 immunofluorescence staining. F, I Merge of mpeg1:GFP, F4/80 and Hoechst. K, N L-plastin immunofluorescence staining. L, O Merge of mpeg1:GFP, L-plastin and Hoechst. Arrows represent colocalization events. P Percentage of colocalization of endogenous eGFP (average from tail, trunk and head sections) with anti-GFP (97.5%), F4/80 (41.4%) and L-plastin (21.6%). Scale bar: 50 µm; n=3. Related to Fig. 1. Figure S2. Time-dependent regulation of KEGG pathways in the injured dorsal iris of Notophthalmus viridescens. A time course expression analysis was performed of the dorsal iris through 4 dpl. The shown pathways were overrepresented amongst transcripts that exhibited time-dependent regulatory patterns. The displayed expression values in each box represent the expression of homologous transcripts, ordered from left to right by time beginning with the intact iris. Color scale represents Z-score of expression values. Related to Fig. 2B. Figure S3. Clodronate treatment does not affect the survival of iPECs during the early stages of lens regeneration. (A) TUNEL assay was used to visualize apoptotic nuclei from control- and clodronate-treated animals at 1, 4, 10, 15, and 30 dpl (paraffin embedded tissue). Dashed lines were used to mark the iris epithelium. Inset images of the dorsal iPECs highlight the effects of macrophage depletion on cell survival. As expected, TUNEL+ nuclei were observed in the vitreous and aqueous chambers of clodronate-liposome treated eyes (arrows) but not in PBS-liposome treated eyes. At 15 (Stage IV-V) and 30 dpl (Stage VIII) TUNEL+ nuclei were found in the lens epithelial layer [file 13287_2024_3740_MOESM2_ESM.docx]

**Supplemental informatio**
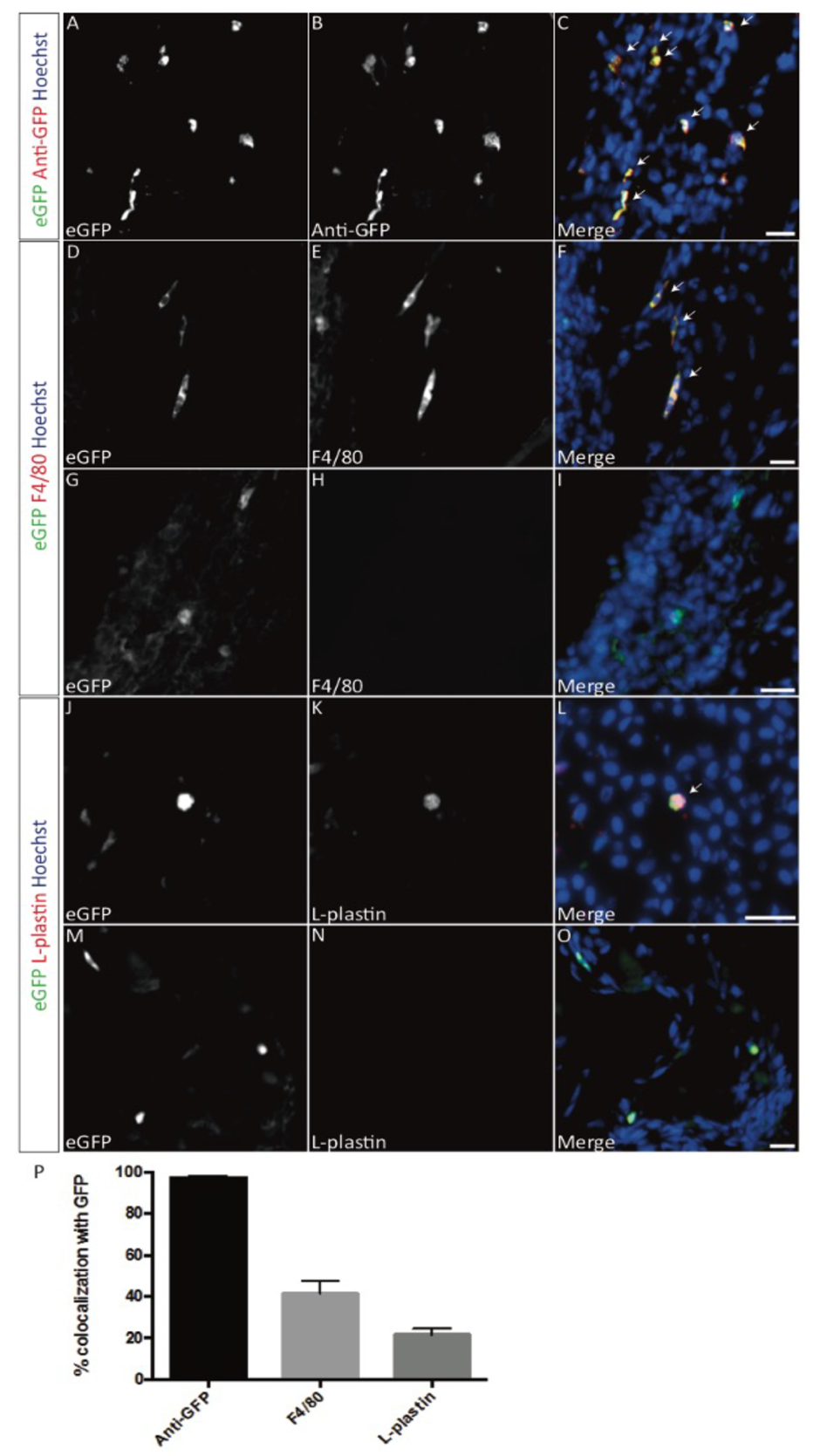

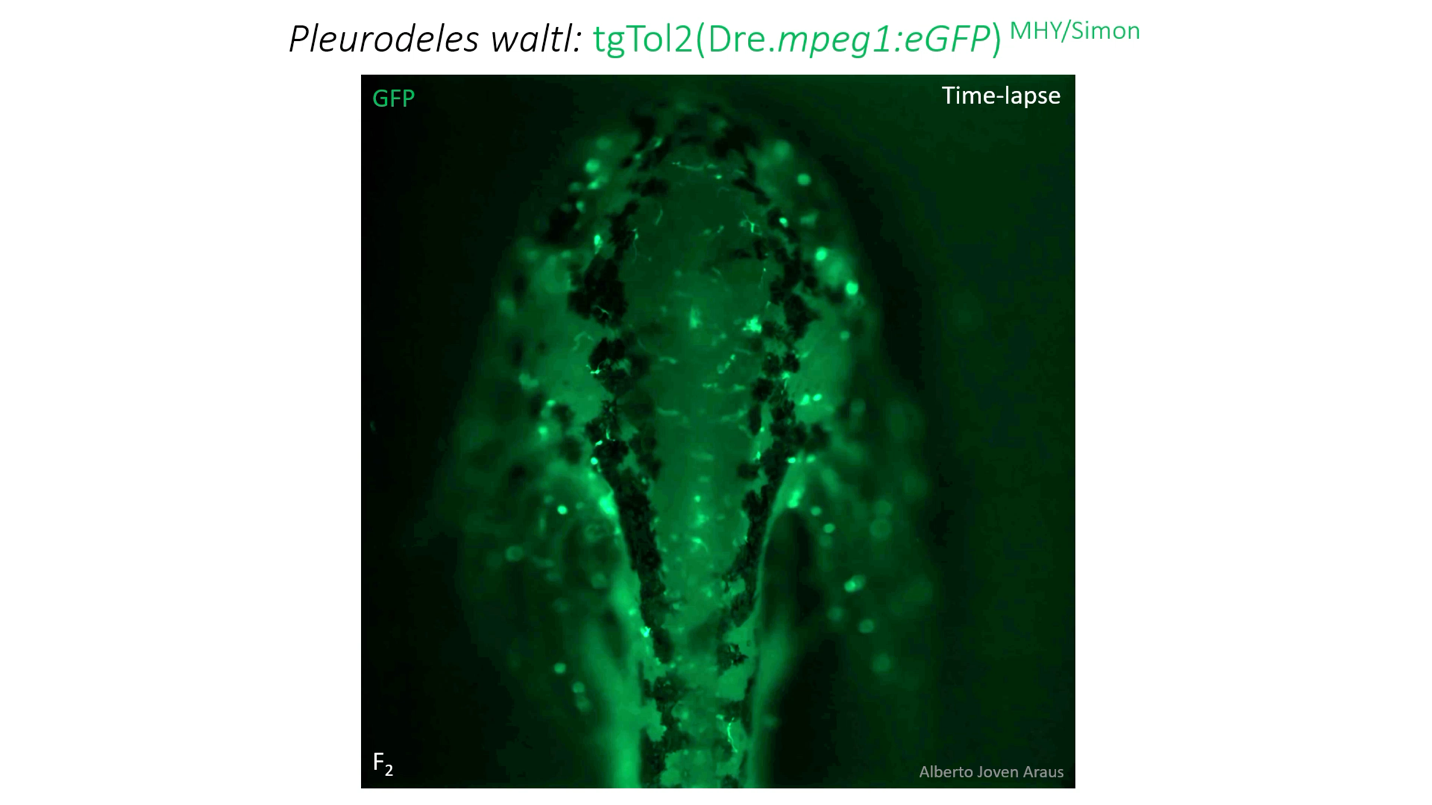
**n**

**Supplementary Figure S1.** ***mpeg1:GFP* transgenic newts enable the *in vivo* labeling of macrophages.** (**A, D, G, J, M)** Representative fluorescence images of sections of 5-month-old *mpeg1:eGFP* newts showing presence of eGFP+ cells in the tail, trunk and head sections (**B,**) Anti-GFP immunofluorescence staining. (**C**) Merge of *mpeg1:eGFP* endogenous fluorescence, anti-GFP and Hoechst. (**E, H**) F4/80 immunofluorescence staining. (**F, I**) Merge of *mpeg1:eGFP*, F4/80 and Hoechst. (**K, N**) L-plastin immunofluorescence staining. (**L, O**) Merge of *mpeg1:eGFP*, L-plastin and Hoechst. Arrows represent colocalization events. (**P**) Percentage of colocalization of endogenous eGFP (average from tail, trunk and head sections) with anti-GFP (97.5%), F4/80 (41.4%)
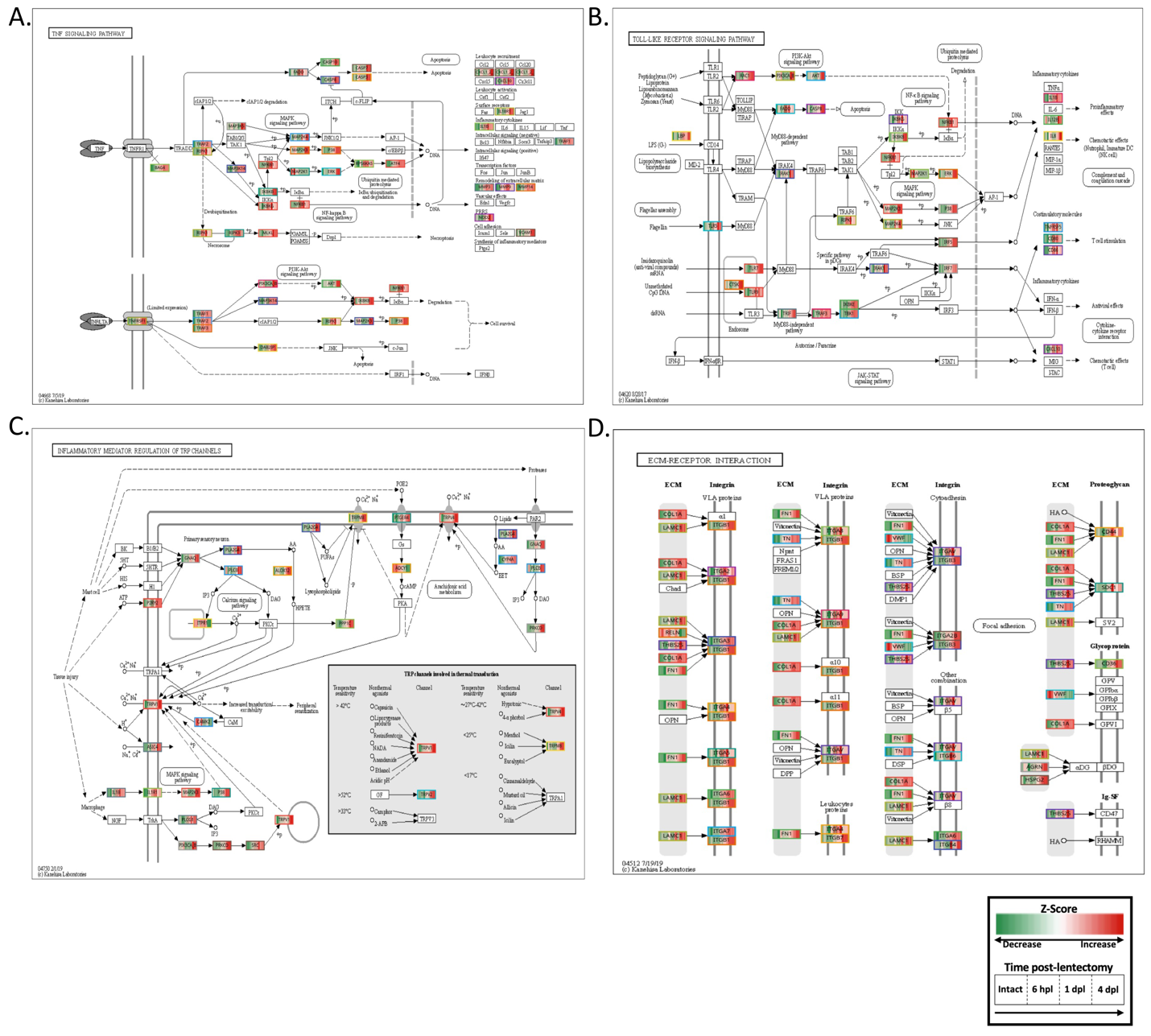
and L-plastin (21.6%). Scale bar: 50 µm; n=3. Related to Figure 1.

**Supplementary Figure S2. Time-dependent regulation of KEGG pathways in the injured dorsal iris of *Notophthalmus viridescens*.** A time course expression analysis was performed of the dorsal iris through 4 dpl. The shown pathways were overrepresented amongst transcripts that exhibited time-dependent regulatory patterns. The displayed expression values in each box represent the expression of homologous transcripts, ordered from left to right by time beginning with the intact iris. Color scale represents Z-score of expression values. Related to figure 2B.

**
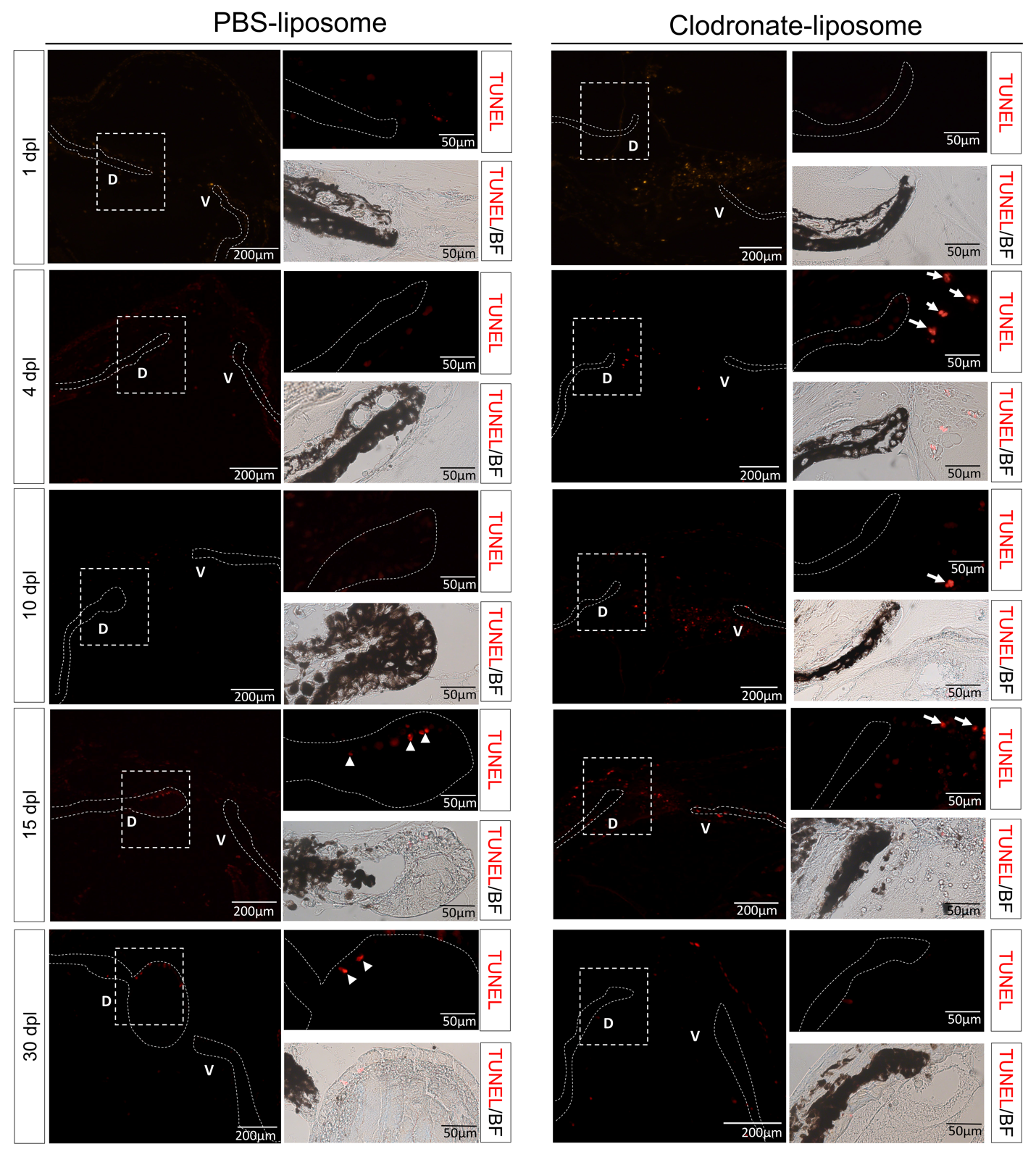
**

**Supplementary Figure S3. Clodronate treatment does not affect the survival of iPECs during the early stages of lens regeneration.** (A)TUNEL assay was used to visualize apoptotic nuclei from control- and clodronate-treated animals at 1, 4, 10, 15, and 30 dpl (paraffin embedded tissue). Dashed lines were used to mark the iris epithelium. Inset images of the dorsal iPECs highlight the effects of macrophage depletion on cell survival. As expected, TUNEL+ nuclei were observed in the vitreous and aqueous chambers of clodronate-liposome treated eyes (arrows) but not in PBS-liposome treated eyes. At 15 (Stage IV-V) and 30 dpl (Stage VIII) TUNEL+ nuclei were found in the lens epithelial layer of PBS-liposome treated eyes (arrowhead); n=6 per time point. Scale bars: 200µm (overviews, left) and 50µm (insets, right).


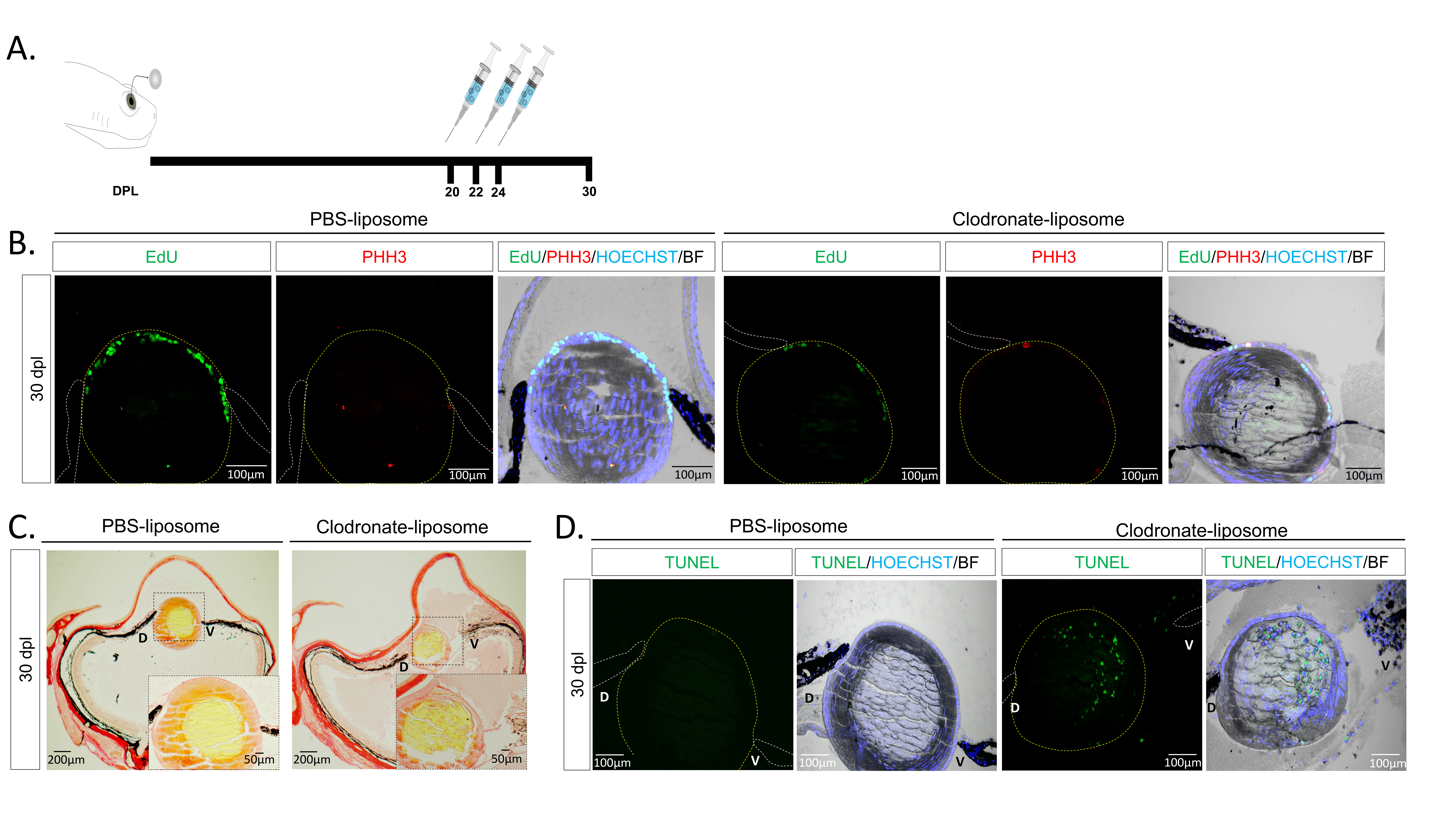


**Supplementary Figure S4. Late clodronate-liposome administration impairs lens growth by increasing apoptosis instead of affecting proliferation. (A)** Schematic representation of experimental design. Clodronate or PBS liposomes were injected intraocularly in the aqueous chamber at 20, 22, and 24 dpl in the presence of the regenerating lens. **(B)** Clodronate liposome administration at 20 dpl did not inhibit the proliferation and mitosis levels of lens epithelial cells; n=6. Scale bars: 100µm (paraffin embedded tissue). **(C)** Picrosirius red staining revealed a stronger collagen staining (red) in the vitreous and aqueous chamber of the clodronate liposome treated eyes; n=6. Scale bars: 100µm (paraffin embedded tissue). **(D)** Apoptotic cells were detected inside the lens fibers and at the surrounding area of the ventral iris following macrophage depletion at 20 dpl; n=6. Scale bars: 100µm. Related to figure 7 (paraffin embedded tissue).

| **Gene** | **Forward Primer** | **Reverse Primer** | **PCR product (bp)** | **Target Sequence** |
| --- | --- | --- | --- | --- |
| P53 | TATGGCACCACCACGCTATG | AATGATGGTCATGCTCCCCC | 107 | M1034543_PLEWA04 |
| CDK2 | CGGTATCCCTTTGCCACTCA | TCAGCAAGCTTGATAGCCCC | 144 | M0441300_PLEWA04 |
| E2F1 | TGCCGGCCAAAAGAAAGTTG | CTATTCCGGTCCGGCCTTTT | 90 | M0222145_PLEWA04 |
| SOCS3 | TACATGCCAAACAGCGGACT | GTGCCCGTTGACAGTTTTCC | 162 | M0047129_PLEWA02 |
| CSFR | TGGCACTGATAGTCGCAGTC | AAACACCGGTTCCCCTTTGT | 101 | M0373017_PLEWA04 |
| COX-2 | GGGAGCTTTGATTTTCGCCC | CGGTCATACACACTCCTCGG | 92 | M0026016_PLEWA04 |
| IL1b | CCGCAGGATAGTGGTGATCG | TGTCCCCAAACAGACACCTG | 77 | M0346665_PLEWA04 |
| TGFB2 | AAACCCAGAAGCATCAGCCT | GCTGCACTTGCAGGACTTTAC | 133 | M0018968_PLEWA04 |
| TGFB3 | AACGCTTCATCAGTGGGAGG | CACTCCCGCACATTCTCTGT | 85 | M0100785_PLEWA04 |
| MMP3/10a | CAGGCTGAGAGGGAGAGTCA | GCGCACACTAAGAGGAGTGA | 102 | M1347552_PLEWA04 |
| MMP9 | GAGGGGGTGCAGTACCTCTA | AGCTGTTACTGGGGTTGTCG | 111 | M0114547_PLEWA04 |
| TAII70 | CCTGGGAAGCATTTGGTAGA | TTCACGAGCTGTCTGTGGAG | 137 | M0451743_PLEWA04 |

Table S1. Oligos and target sequences used for RT-qPCR
